# Probing the Framework Metal Dependent Properties of Actinide-Centered Polyoxoalkoxide Sandwich-Type Complexes

**DOI:** 10.1021/acs.inorgchem.5c00780

**Published:** 2025-04-30

**Authors:** Dominic Shiels, Adriana C. Berlfein, Barbara M. T. C. Peluzo, Lauren M. Lopez, Andrew W. Mitchell, William W. Brennessel, Matthias Zeller, Matthew R. Crawley, Suzanne C. Bart, Michael T. Ruggiero, Ellen M. Matson

**Affiliations:** † Department of Chemistry, University of Rochester, Rochester, New York 14627, United States; ‡ H. C. Brown Laboratory, James Tarpo, Jr. and Margaret Tarpo, Department of Chemistry, Purdue University, West Lafayette, Indiana 47907, United States; § Department of Chemistry, University at Buffalo, The State University of New York, Buffalo, New York 14620, United States

## Abstract

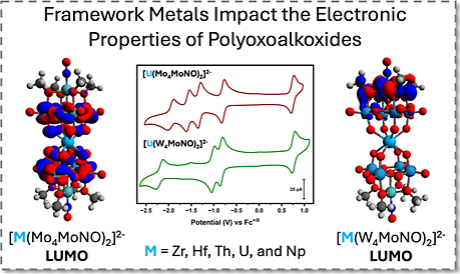

Development of a simple and scalable synthesis of (TBA)_3_[W_5_O_18_MoNO] provides for the formation of the mixed-metal lacunary polyoxoalkoxide, (TBA)_2_[W_4_O_13_(OMe)_4_MoNO][Na(MeOH)]. This complex was used to synthesize a series of polyoxoalkoxide sandwich-type complexes with the general formula (TBA)_2_[M{W_4_O_13_(OMe)_4_MoNO}_2_], where M = Zr(IV), Hf(IV), Th(IV), U(IV), and Np(IV). Compared to the analogous all-molybdenum complexes, the series have drastically different optical and redox properties. The results indicate that framework metal substitution acts as a tool for “orbital engineering”, with Density Functional Theory (DFT) calculations revealing that the major consequence of incorporation of tungsten into the complexes is localization of LUMO and LUMO+1 on the molybdenum centers remaining in the molecule. The change in the distribution of the frontier orbitals translates to discrepancies in the electronic properties of the series. Given the rarity of polyoxometalate complexes featuring a U(V) ion, one electron oxidation of (TBA)_2_[U(IV){W_4_O_13_(OMe)_4_MoNO}_2_] was pursued. Isolation of the corresponding U(V) centered sandwich-type complex is reported, only the second example of U(V)-polyoxometalate complex described to date.

## Abstract 2

Development of a simple and scalable synthesis of (TBA)_3_[W_5_O_18_MoNO] provides for the formation of the mixed-metal lacunary polyoxoalkoxide, (TBA)_2_[W_4_O_13_(OMe)_4_MoNO][Na(MeOH)].

## Introduction

Polyoxometalates (POMs), anionic molecular metal oxide clusters typically based on tungsten(VI), molybdenum(VI), or vanadium(V), are growing in popularity as versatile “ligands” in actinide research.^1−9^ This is largely a consequence of their high molecular weights (≈1000–20,000 g mol^–1^), which facilitates stoichiometric reactions between POMs and actinides requiring minimal quantities of the radiological precursor. This trait reduces challenges associated with both scarcity of some actinide ions, as well as safety concerns.^10−14^ Actinide-polyoxometalate (An-POM) complexes, mostly produced as alkali metal salts using aqueous methods, are readily isolated as high quality single crystals, allowing direct insight into the coordination preferences of the actinides using single crystal X-ray diffraction (SCXRD) experiments.^15^

The vast majority of reported An-POM complexes have been isolated using aqueous methods, with the bulk of these employing polyoxotungstates (POTs) as the “ligand” of choice in sandwich-type complexes (i.e., An(POT)_2_).^16−18^ This is attributed to the stability of lacunary POTs when compared to the corresponding polyoxomolybdate (POMo) derivatives.^17^ An–POMo complexes reported to date typically avoid the use of a lacunary precursor, with a set of An(IV) centered Silverton-type clusters (general formula [AnMo_12_O_42_]^8–^) representing one of the most widely studied systems.^16,19−22^ Consequently, there are few examples of sandwich-type complexes which feature POMos as ligands (i.e., An(POMo)_2_), and thus there are almost no examples of a pair of isostructural An-POM complexes, differing by only the framework metal. To our knowledge, the only example of such a pair is [Th(PW_11_O_39_)_2_]^10–^ and [Th(PMo_11_O_39_)_2_]^10–^, which were isolated by two independent research teams; a detailed study comparing their properties has not been performed.^15,23^ When this is paired with the fact that many studies in this field largely focus on solid state structural analysis, very little is known about how variation of the framework metal impacts the optical and electrochemical properties of the complexes.

A rare example of a stable and isolable lacunary POMo comes from Proust and co-workers. Treatment of (TBA)_4_[α-Mo_8_O_26_] (TBA = tetrabutylammonium) with hydroxylamine in methanol, in the presence of sodium cations, results in the formation of the lacunary Lindqvist-type polyoxoalkoxide (TBA)_2_[Mo_5_O_13_(OMe)_4_NO][Na(MeOH)] (**1-NaMo**_**5**_).^24^ This complex features a linear molybdenum nitrosyl unit, rarely observed in POM chemistry. The oxidation state distribution of **1-NaMo**_**5**_ is suggested to contain four Mo(VI) centers and a single Mo(II) center, which is bound to the nitrosyl group.^24−26^ The formal assignment of linear {Mo–NO}^3+^ groups as being composed of a Mo(II) ion and an {NO}^+^ ligand is in line with literature precedent.^27,28^ However, it is worth noting that formal oxidation state assignments of metals that form part of an M-NO unit is not trivial, with {NO}^+^, {NO}^−^, and {NO}^3–^ assignments being invoked depending on geometry and the relative localization of bonding electron density, as discussed by Klüfers and co-workers.^29^ Therefore, the alternative Enemark–Feltham nomenclature is often used, avoiding formal oxidation state assignments of the metal/ligand, and instead noting the number of metal d-electrons involved in bonding.^30,31^ Using this nomenclature, the {Mo–NO}^3+^ unit is instead described in this case as {Mo–NO}^4^.

Recently, our group utilized **1-NaMo**_**5**_ for the synthesis of a series of sandwich-type complexes with the general formula (TBA)_2_[M{Mo_5_O_13_(OMe)_4_NO}_2_] (where M = Zr(IV), Hf(IV), Th(IV), U(IV), and Np(IV)) under nonaqueous conditions.^32,33^ While the expected square-antiprismatic eight coordinate geometry of the central metal ion persists across the series, electronic properties vary with the identity of the central metal. Examination of the optical properties of the series by electronic absorption spectroscopy reveals the presence of only a weak absorption in the visible region of the spectrum (assigned to the {Mo–NO}^4^ unit of the polyoxoalkoxide ligand) when the central metal possessed a d^0^ (Zr(IV) or Hf(IV)) or f^0^ (Th(IV)) valence electronic configuration.^33^ This was contrasted by the presence of a much broader and more intense An(5f) → Mo(4d) metal-to-ligand charge transfer (MLCT) absorption spanning most of the visible region when the central metal featured partially occupied valence f-orbitals (U(IV), 5f^2^; Np(IV), 5f^3^).^32,33^ Similarly, the redox properties of the series were found to depend on the nature of the central metal (M). Analysis of the Zr/Hf centered complexes by cyclic voltammetry (CV) showed two reversible reduction events, attributed to reduction of the Mo(VI) centers of the polyoxoalkoxide units. However, incorporation of an actinide (Th, U, or Np) enhances the reducibility of the polyoxoalkoxide units, with the CV of these complexes possessing four reversible reduction events.^32,33^

The interesting electronic properties of these sandwich-type complexes are hypothesized to involve the empty 4d orbitals of the Mo(VI) centers in the polyoxoalkoxide ligands. Therefore, these properties are expected to be sensitive to the identity of the framework metal. For example, the MLCT energy and intensity should vary significantly with the nature of the LUMO, which can change in both energy and spatial distribution upon framework metal substitution.^34^ Similar variations in the reduction properties of the complexes would also be expected, with metals lower down the periodic table being more difficult to reduce (i.e., POTs are reduced at more cathodic potentials than the corresponding POMos).^34−37^ This may shift the reduction potentials substantially negative, meaning that being able to study this phenomenon in organic solvent is preferred; nonaqueous conditions provide access to a wider electrochemical window and can prevent unwanted side reactions upon changing the redox state of the investigated compound.^20,38,39^

In a previous report from Villanneau and co-workers, where **1-NaMo**_**5**_ was used to synthesize sandwich-type complexes centered with a number of large M(II) and M(III) cations, the authors also describe the formation of a Bi(III) centered sandwich-type complex, (TBA)_3_[Bi{W_4_O_13_(OMe)_4_MoNO}_2_].^25^ This complex was synthesized by first isolating (TBA)_2_[W_4_O_13_(OMe)_4_MoNO][Na(MeOH)] (**1-NaW**_**4**_**Mo**), and then treating that directly with BiCl_3_. Comparison of the crystallographic data and infrared spectrum of (TBA)_3_[Bi{W_4_O_13_(OMe)_4_MoNO}_2_] to its all-molybdenum analogue, (TBA)_3_[Bi{Mo_5_O_13_(OMe)_4_MoNO}_2_], shows minor variations in both average bond lengths and stretching frequencies. Unfortunately, no further characterization data was reported, and therefore it is difficult to verify how incorporation of W into the framework effects the electronic properties of the assembly.^25^ No additional studies have been reported utilizing **1-NaW**_**4**_**Mo**, likely due to the cumbersome and low yielding synthesis of the lacunary assembly.^25,26,40,41^

**Figure 1:**
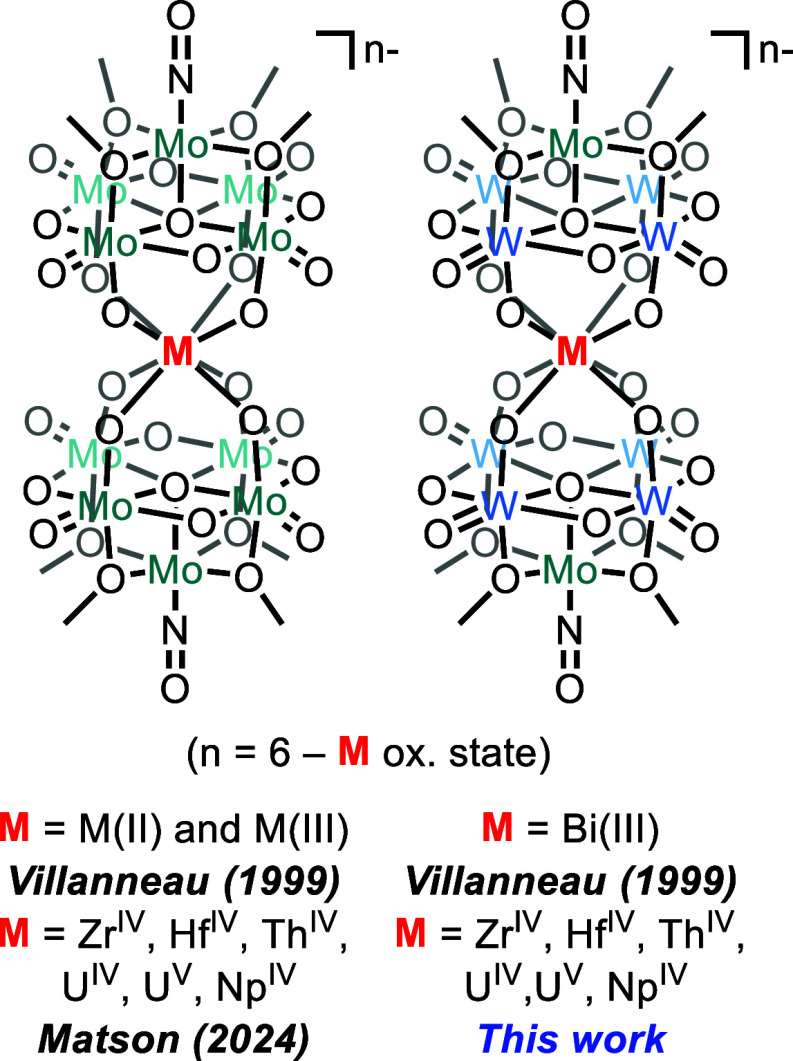
Overview of the previous work in this area and the complexes studied in this work.

Here, we describe an optimized synthetic approach for the large-scale synthesis of (TBA)_3_[W_5_O_18_MoNO] (ca. 8 g per synthesis), which acts as a precursor in the synthesis of **1-NaW**_**4**_**Mo**. With the mixed-metal lacunary polyoxoalkoxide in hand, we synthesize a series of sandwich-type complexes with the general formula (TBA)_2_[M{W_4_O_13_(OMe)_4_MoNO}_2_], where M = Zr(IV), Hf(IV), Th(IV), U(IV), and Np(IV) (Figure 1). The complexes are characterized using ^1^H NMR spectroscopy, ^17^O NMR spectroscopy, SCXRD, and elemental analysis. Attention was then turned to the optical properties of the series. While the Zr(IV), Hf(IV), and Th(IV) centered complexes behave similarly to their previously reported all-molybdenum analogues, the MLCT process between central U(IV) or Np(IV) metal and the polyoxoalkoxide ligand is found to be sensitive to the identity of the framework metal (i.e., Mo vs W). Further deviations from the behavior of the all-molybdenum derivatives are also noted in the electrochemical properties of the complexes. The reduction chemistry of the series is found to be much less sensitive to the nature of the central M(IV) ion present, while the small potential difference between the first and second reduction events indicates minimal electronic communication between the two polyoxoalkoxide ligands of the sandwich-type complexes. In order to rationalize the causes of the framework dependent optical and redox properties of the series, density functional theory (DFT) calculations are used to give insights into the electronic structure.

Finally, we describe the formation of the second U(V)-centered sandwich-type polyoxometalate complex. Oxidation of (TBA)_2_[U{W_4_O_13_(OMe)_4_MoNO}_2_] affords isolation of a rare example of a U(V) containing POM sandwich-type complex, (TBA)[U{W_4_O_13_(OMe)_4_MoNO}_2_]. Characterization of this complex is described (multinuclear NMR spectroscopy, SCXRD, and electronic absorption spectroscopy).

## Experimental Section

### General Considerations

All air- and moisture-sensitive manipulations with neptunium were performed in an MBraun negative pressure UHP argon atmosphere Drybox. Air- and moisture-sensitive manipulations with all other complexes were carried out using a standard high-vacuum line, Schlenk techniques, or an MBraun inert atmosphere Drybox containing an atmosphere of purified dinitrogen. The MBraun glovebox was equipped with a cold well designed for freezing samples in liquid nitrogen, as well as a −35 °C freezer for cooling samples and crystallizations. Solvents for sensitive manipulations were dried and deoxygenated using literature procedures with a Seca solvent purification system or a glass contour solvent purification system (Pure Process Technology, LLC) and stored over activated 4 Å molecular sieves (Fisher Scientific) prior to use. Deuterated solvents were purchased from Cambridge Isotope Laboratories, dried with molecular sieves and degassed by three freeze–pump–thaw cycles. 40% ^17^O enriched H_2_O was purchased from CortecNet and used as received. (TBA)_4_[Mo_8_O_26_],^42^ (TBA)_2_[WO_4_],^41^ (TBA)_2_[W_4_O_13_(OMe)_4_MoNO][Na(MeOH)] (**1-NaW**_**4**_**Mo**),^25^ UCl_4_,^43^ ThCl_4_.2DME,^44^ and NpCl_4_(DME)_2_^45^ were synthesized according to literature procedures.

### Safety Considerations

Caution! ^237^Np represents a health risk due to its α and γ emission and its decay to the short-lived ^233^Pa isotope (*t*_1/2_ = 27.0 days), which is a strong β and γ emitter. All studies with Np were conducted in a laboratory equipped for radioactive materials. All studies were modeled on depleted uranium prior to working with ^237^Np. Depleted uranium (primary isotope ^238^U) is a weak α-emitter (4.197 MeV) with a half-life of 4.47 × 10^9^ years, and ^232^Th is a weak α-emitter (4.082 MeV) with a half-life of 1.41 × 10^10^ years; manipulations and reactions should be carried out in monitored fume hoods or in an inert atmosphere Drybox in a radiation laboratory equipped with α and β counting equipment.

The scarcity of neptunium in combination with the relatively high specific radioactivity of ^237^Np requires syntheses to be performed on small scales (<15 mg Np). Fortunately, the high molecular weight of polyoxometalates allows for stoichiometric reactions to be performed with exceptionally small quantities of actinide starting materials, making extension of this chemistry to neptunium favorable.

### Synthesis of (TBA)_3_[W_5_O_18_MoNO]

In a 250 mL glass pressure vessel, (TBA)_2_[WO_4_] (25 g, 34.1 mmol, 40 equiv) was dissolved in MeCN (70 mL) (note: a round-bottom flask and condenser can be used as an alternative). After complete dissolution, (TBA)_4_[α-Mo_8_O_26_] (1.84 g, 0.85 mmol, 1 equiv) and NH_3_OHCl (2.37 g, 34.1 mmol, 40 equiv) were added and the mixture was heated to reflux. The mixture was heated for 4 h during which time it changed from colorless to yellow and finally to brown. The heating was stopped, and the solution was gravity filtered through fluted filter while hot to remove any precipitate (in some cases no precipitate was observed and this step can be skipped). The reaction mixture was then allowed to cool to room temperature which led to the immediate formation of green crystals. The solution was further cooled to −30 °C overnight by storing in a freezer. The crystals were isolated by vacuum filtration and then washed with MeOH (2 × 10 mL) and Et_2_O (2 × 20 mL). The crystals were then dried under vacuum (8.32 g, 59% yield). ^1^H NMR (500 MHz, CD_3_CN): δ 0.98 (t, *J* = 7.4 Hz, 36 H), 1.40 (h, *J* = 7.4 Hz, 24 H), 1.64 (m, 24 H), 3.17 (m, 24 H). λ_max_ (MeCN) = 676 nm (ε = 18 mol^–1^ dm^3^ cm^–1^). FTIR (neat) cm^–1^: 2960 (m), 2874 (m), 1578 (s), 1482 (m), 1381 (w), 1152 (w), 1028 (w), 954 (s), 884 (w), 779 (s), 613 (w), 568 (m). Anal. Calcd for C_48_H_108_N_4_O_19_W_5_Mo (mol. wt. 2060.551 g mol^–1^): C, 27.98%; H 5.28%; N, 2.72%. Found: C, 28.08%; H, 5.189%; N, 2.864%.

### Synthesis of ^17^O Enriched (TBA)_3_[W_5_O_18_MoNO]

In a 15 mL glass pressure vessel, (TBA)_2_[WO_4_] (0.5 g, 0.68 mmol, 40 equiv) was dissolved in MeCN (5 mL) (note: a round-bottom flask and condenser can be used as an alternative). After complete dissolution, H_2_^17^O (25 μL, 1.36 mmol, 80 equiv, 40% enriched) was added and the solution was stirred at room temperature for 15 minutes. (TBA)_4_[α-Mo_8_O_26_] (37 mg, 0.017 mmol, 1 equiv) and NH_3_OHCl (47 mg, 0.68 mmol, 40 equiv) were then added and the mixture was heated to reflux. The mixture was heated for 4 h during which time it changed from colorless to yellow to green and finally to brown. The heating was stopped, and the solution was passed through a PTFE syringe filter while hot. The reaction mixture was then allowed to cool to room temperature and then concentrated to ca. 3 mL, during which time green crystals begin to form. The solution was then stored at −30 °C overnight. The yellow/brown mother liquor was decanted and the crystals were washed with MeOH (2 mL) and Et_2_O (2 × 5 mL). The crystals were then dried under vacuum (0.16 g, 57% yield). ^17^O NMR (67.8 MHz, CD_3_CN): δ −89.5 (μ_6_-O, 1 O), 395.0 (W–O–W, 8 O), 510.5 (Mo–O–W, 4 O), 724.4 (W = O, 1 O), 727.7 (W = O, 4 O). Additional characterization was in line with that given above.

### Synthesis of (TBA)_2_[W_4_O_13_(OMe)_4_MoNO][Na(MeOH)] (**1-NaW**_**4**_**Mo**)

This procedure is based on the method originally described by Villanneau and Proust.^25^ In a glovebox, (TBA)_3_[W_5_O_18_MoNO] (3 g, 1.46 mmol, 1 equiv) was suspended in MeOH (25 mL) in a 75 mL glass pressure vessel (note: an appropriately sized round-bottom flask and condenser can be used as an alternative, though the reaction should be performed under an inert atmosphere). NaOEt (0.40 g, 5.82 mmol, 4 equiv) was added, the vessel was sealed, and it was removed from the glovebox (note: NaOEt was preferred over NaOMe due to higher solubility though the final yield was unchanged). The mixture was refluxed for 24 h leading to the formation of a red/purple suspension. The mixture was allowed to cool to room temperature before filtering through a medium porosity glass frit in air (occasionally a fine precipitate would pass through the frit and in this case, the solution was separated from the fine solid by passing through a PTFE syringe filter or by centrifugation). The red/purple solution was then concentrated to ca. 7 mL. Some purple solid may form during this process. The solution was warmed to redissolve any precipitate before slowly cooling to room temperature and then to −30 °C to induce crystal formation. After storing at −30 °C overnight, the red mother liquor was decanted, and the remaining purple crystals were washed with Et_2_O (4 × 10 mL). The purple powder was then dried under vacuum (0.61 g, 24% yield). Single crystals were obtained by vapor diffusion of Et_2_O into a saturated solution of the compound in MeOH. ^1^H NMR (500 MHz, CD_3_CN): δ 0.97 (t, *J* = 7.3 Hz, 24 H), 1.36 (h, *J* = 7.4 Hz, 16 H), 1.61 (m, 16 H), 3.11 (m, 16 H), 4.59 (–OMe). λ_max_ (MeCN) = 544 nm (ε = 72 mol^–1^ dm^3^ cm^–1^). FTIR (neat) cm^–1^: 3418 (br), 2957 (m), 2874 (w), 2816 (w), 1639 (s), 1483 (m), 1381 (w), 1151 (w), 1034 (s) 946 (s), 887 (s), 798 (w), 712 (s), 631 (m), 560 (m). Anal. Calcd for C_37_H_88_N_3_O_19_W_4_MoNa (mol. wt. 1733.413 g mol^–1^): C, 24.64%; H 5.12%; N, 2.42%. Found: C, 24.610%; H, 4.897%; N, 2.252%.

### ^17^O Enrichment of (TBA)_2_[W_4_O_13_(OMe)_4_MoNO][Na(MeOH)] (**1′-NaW**_**4**_**Mo**)

(TBA)_2_[W_4_O_13_(OMe)_4_MoNO][Na(MeOH)] (100 mg, 0.07 mmol, 1 equiv) was dissolved in anhydrous MeOH (5 mL) forming a purple solution. 40% ^17^O enriched H_2_O (6.5 μL, 0.36 mmol, 5 equiv) was added and the solution was stirred at 50 °C for 3 h. The solution was allowed to cool temperature and then the volatiles were removed under vacuum. ^17^O NMR spectroscopy of the crude material showed successful ^17^O enrichment but also revealed the presence of a significant amount of residual water. Therefore, the material was purified by recrystallization. Vapor diffusion of Et_2_O into a saturated solution of the crude material dissolved in anhydrous MeOH led to the formation of large purple crystals. The mother liquor was decanted, and the crystals were washed with Et_2_O (can lead to loss of crystallinity) and dried under vacuum overnight (66 mg, 66% yield). ^17^O NMR (67.8 MHz, CD_3_CN): δ −2.5 (μ_5_-O, 1 O), 334.17 (W–O–W, 4 O), 636.3 (W–O–Na, 4 O), 684.0 (W = O, 4 O). Additional characterization was in line with that given above.

It was noted that the material also readily loses ^17^O enrichment upon dissolution in wet MeOH, presumably due to O atom exchange with the H_2_O present in the solvent. Therefore, to avoid loss of enrichment, the reaction, purification, and subsequent reactions should all be performed in anhydrous solvents where possible.

### General Method for Synthesis of (TBA)_2_[M(IV){W_4_O_13_(OMe)_4_MoNO}_2_] Compounds (M = Zr, Hf, Th, U)

In a 20 mL scintillation vial, (TBA)_2_[W_4_O_13_(OMe)_4_MoNO][Na(MeOH)] (0.3 g, 0.22 mmol, 2 equiv) was dissolved in MeOH (7 mL) forming a dark purple solution. The appropriate metal(IV) chloride (0.12 mmol, 1.1 equiv) was dissolved in MeOH (3 mL) and was slowly added to the mixture. A precipitate formed immediately upon addition. The suspension was stirred for a further 15 min before filtering through a bed of Celite (approximately 1 cm) and washing with MeOH (5 mL). The product was extracted with DCM until washings ran clear (approximately 10–15 mL). The solvent was removed under reduced pressure. The products were found to be suitably pure at this point by ^1^H NMR spectroscopy and elemental analysis for most applications, however recrystallization by slow evaporation of saturated solutions of the product dissolved in either MeCN or THF can be performed if desired. Single crystals were obtained by vapor diffusion of Et_2_O into a saturated solution of the products in MeCN. ^17^O enriched materials were obtained by using ^17^O enriched **1′-NaW**_**4**_**Mo**, as prepared above.

### (TBA)_2_[Zr(IV){W_4_O_13_(OMe)_4_MoNO}_2_] (**2-Zr(W**_**4**_**Mo)**_**2**_)

Blue solid (0.19 g, 74% yield). ^1^H NMR (500 MHz, CD_2_Cl_2_): δ 1.10 (s, 24 H), 1.56 (s, 16 H), 1.81 (s, 16 H), 3.33 (s, 16 H), 4.86 (–OMe, s, 24 H). ^17^O NMR (67.8 MHz, CD_2_Cl_2_): δ −8.5 (μ_5_-O, 1 O), 394.3 (W–O–W, 4 O), 567.4 (W–O–Zr, 4 O), 751.4 (W = O, 4 O). λ_max_ (MeCN) = 588 nm (ε = 102 mol^–1^ dm^3^ cm^–1^). Anal. Calcd for C_40_H_96_N_4_O_36_W_8_Mo_2_Zr (mol. wt. 2963.044 g mol^–1^): C, 16.21%; H, 3.27%; N, 1.89%. Found: C, 15.819%; H, 3.083%; N, 1.906%.

### (TBA)_2_[Hf(IV){W_4_O_13_(OMe)_4_MoNO}_2_] (**3-Hf(W**_**4**_**Mo)**_**2**_)

Blue solid (0.18 g, 68% yield). ^1^H NMR (500 MHz, CD_2_Cl_2_): δ 1.09 (t, *J* = 7.3 Hz, 24 H), 1.55 (h, *J* = 7.4 Hz, 16 H), 1.79 (m, 16 H), 3.31 (m, 16 H), 4.85 (–OMe, s, 24 H). ^17^O NMR (67.8 MHz, CD_2_Cl_2_): δ −8.3 (μ_5_-O, 1 O), 394.1 (W–O–W, 4 O), 552.9 (W–O–Hf, 4 O), 750.0 (W = O, 4 O). λ_max_ (MeCN) = 590 nm (ε = 92 mol^–1^ dm^3^ cm^–1^). Anal. Calcd for C_40_H_96_N_4_O_36_W_8_Mo_2_Hf (mol. wt. 3050.310 g mol^–1^): C, 15.75%; H, 3.17%; N, 1.84%. Found: C, 15.971%; H, 2.998%; N, 1.722%.

### (TBA)_2_[Th(IV){W_4_O_13_(OMe)_4_MoNO}_2_] (**4-Th(W**_**4**_**Mo)**_**2**_)

Blue solid (0.20 g, 74% yield). ^1^H NMR (500 MHz, CD_2_Cl_2_): δ 1.09 (t, *J* = 7.3 Hz, 24 H), 1.55 (h, *J* = 7.4 Hz, 16 H), 1.80 (m, 16 H), 3.31 (m, 16 H), 4.86 (–OMe, s, 24 H). ^17^O NMR (67.8 MHz, CD_2_Cl_2_): δ −13.8 (μ_5_-O, 1 O), 390.9 (W–O–W, 4 O), 595.4 (W–O–Th, 4 O), 751.9 (W = O, 4 O). λ_max_ (MeCN) = 578 (ε = 134 mol^–1^ dm^3^ cm^–1^). Anal. Calcd for C_40_H_96_N_4_O_36_W_8_Mo_2_Th.2CH_3_OH (mol. wt. 3103.858 g mol^–1^, with solvent 3167.942 g mol^–1^): C, 15.92%; H, 3.31%; N, 1.77%. Found: C, 16.242%; H, 3.188%; N, 1.731%.

### (TBA)_2_[U(IV){W_4_O_13_(OMe)_4_MoNO}_2_] (**5-U(W**_**4**_**Mo)**_**2**_)

Green solid (0.22 g, 82% yield). ^1^H NMR (500 MHz, CD_2_Cl_2_): δ −4.80 (s, 16 H), −4.22 (s, 16 H), −3.58 (s, 16 H), −2.57 (t, *J* = 7.1 Hz, 24 H), 10.16 (–OMe, s, 24 H). ^17^O NMR (67.8 MHz, CD_2_Cl_2_): δ −40.6 (μ_5_-O, 1 O), 384.7 (W–O–W, 4 O), 712.7 (W = O, 4 O), 992.2 (W–O–U, 4 O). λ_max_ (MeCN) = 564 nm (ε = 205 mol^–1^ dm^3^ cm^–1^), 564–624 nm (broad peak ε = ca. 170–200 mol^–1^ dm^3^ cm^–1^), 660 nm (ε = 193 mol^–1^ dm^3^ cm^–1^), 688 nm (ε = 193 mol^–1^ dm^3^ cm^–1^), 502 nm (ε = 223 mol^–1^ dm^3^ cm^–1^), 1080 nm (ε = 83 mol^–1^ dm^3^ cm^–1^), 1130 nm (ε = 188 mol^–1^ dm^3^ cm^–1^). Anal. Calcd for C_40_H_96_N_4_O_36_W_8_Mo_2_U·2CH_3_OH (mol. wt. 3109.849 g mol^–1^, with solvent 3173.933 g mol^–1^): C, 15.89%; H, 3.30%; N, 1.77%. Found: C, 16.252%; H, 3.172%; N, 1.718%.

### Synthesis of (TBA)_2_[Np(IV){W_4_O_13_(OMe)_4_MoNO}_2_] (**6-Np(W**_**4**_**Mo)**_**2**_)

NpCl_4_(DME)_2_ (5 mg, 0.009 mmol, 1 equiv; DME = dimethoxyethane) was weighed into a 5 mL vial and sealed with a septum cap before being taken out of the glovebox. In a separate 20 mL vial, (TBA)_2_[W_4_O_13_(OMe)_4_MoNO][Na(MeOH)] (31.8 mg, 0.018 mmol, 2 equiv) was dissolved in MeOH (3 mL) in air and added dropwise to the solid NpCl_4_(DME)_2_ with a syringe. A light blue precipitate formed immediately upon addition. The suspension was gently shaken for 5 min before being filtered over Celite in a glass pipet plugged with a microfiber glass filter. A fine blue powder was collected on the Celite bed and washed with MeOH (5 mL). The product was extracted with dichloromethane (DCM) until washings ran clear (ca. 5 mL). The solvent was removed under a N_2_ flow and the product was subsequently dried under reduced pressure overnight before being brought into the glovebox. Blue solid (18.2 mg, 54% yield). ^1^H NMR (400 MHz, CDCl_3_): δ 1.05 (m, 24 H), 1.29 (s, 16 H), 1.60 (m, 16 H), 3.07 (m, 16 H), 4.89 (–OMe, s, 24 H). ^17^O NMR (54.2 MHz, CDCl_3_): δ −116.2 (μ_5_-O, 1 O), 378.0 (W–O–W, 4 O), 702.6 (W = O, 4 O), 796.1 (W–O–Np, 4 O). λ_max_ (MeCN) = 440 nm (ε = 53 mol^–1^ dm^3^ cm^–1^), 583 nm (ε = 117 mol^–1^ dm^3^ cm^–1^), 708 nm (ε = 65 mol^–1^ dm^3^ cm^–1^), 730 nm (ε = 60 mol^–1^ dm^3^ cm^–1^), 752 nm (ε = 187 mol^–1^ dm^3^ cm^–1^), 826 nm (ε = 40 mol^–1^ dm^3^ cm^–1^), 867 nm (ε = 52 mol^–1^ dm^3^ cm^–1^), 898 nm (ε = 62 mol^–1^ dm^3^ cm^–1^), 978 nm (ε = 40 mol^–1^ dm^3^ cm^–1^), 1218 nm (ε = 8 mol^–1^ dm^3^ cm^–1^), 1529 nm (ε = 30 mol^–1^ dm^3^ cm^–1^). Light blue single crystals were obtained by vapor diffusion of Et_2_O into a saturated solution of the product in acetonitrile (MeCN) at room temperature.

### Synthesis of (TBA)[U(V){W_4_O_13_(OMe)_4_MoNO}_2_] (**7-U(V)(W**_**4**_**Mo)**_**2**_)

#### Method A

In a 20 mL scintillation vial, (TBA)_2_[U{W_4_O_13_(OMe)_4_MoNO}_2_] (50 mg, 0.016 mmol, 1 equiv) was dissolved in MeCN (3 mL). This solution was then addition to a separate 20 mL scintillation vial containing [NO][PF_6_] (28 mg, 0.16 mmol, 10 equiv). The mixture was stirred vigorously, with a black/brown suspension quickly forming. The suspension was stirred for 3 min before it was passed through a bed of Celite (approximately 1 cm). The solid was washed with a small amount of MeCN (2 × 1 mL) before extracting with DCM (ca. 5 mL). The DCM was removed under reduced pressure to afford crude **7-U(V)(W**_**4**_**Mo)**_**2**_ (32 mg, 69% yield). Brown single crystals were obtained by vapor diffusion of pentane into a saturated solution of the product in DCM at −30 °C. ^1^H NMR (500 MHz, CD_2_Cl_2_): δ 1.42 (t, *J* = 7.5 Hz, 24 H), 1.86 (m, 16 H), 2.09 (s, 16 H), 3.47 (m, 16 H), 4.22 (–OMe, s, 24 H). ^17^O NMR (67.8 MHz, CD_2_Cl_2_): δ −118.6 (μ_5_-O, 1 O), 427.7 (W–O–W, 4 O), 725.6 (W–O–U, 4 O), 780.0 (W = O, 4 O). λ_max_ (DCM) = 1010 nm (ε = 41 mol^–1^ dm^3^ cm^–1^), 1182 nm (ε = 25 mol^–1^ dm^3^ cm^–1^), 1546 nm (ε = 90 mol^–1^ dm^3^ cm^–1^).

#### Method B

In a 20 mL scintillation vial, (TBA)_2_[W_4_O_13_(OMe)_4_MoNO][Na(MeOH)] (100 mg, 0.058 mmol, 2 equiv) was dissolved in MeCN (5 mL). The purple solution was added to solid UCl_4_ (11 mg, 0.029 mmol, 1 equiv) in a separate vial with stirring. This led to an immediate formation of a dark brown solution. The mixture was stirred for 10 min before adding to a separate vial containing solid [NO][PF_6_] (50 mg, 0.29 mmol, 10 equiv). A brown suspension formed immediately which was stirred for 3 min before passing through a bed of Celite (approximately 1 cm). The solid was washed with a small amount of MeCN (2 × 1 mL) and then extracted with DCM until the washing ran clear (approximately 10 mL). The volatiles were removed under vacuum to leave a dark brown solid (44 mg, 53% yield). The obtained characterization data matched that given above.

### Physical Measurements

^1^H NMR spectra for neptunium compounds were recorded at room temperature on a Bruker AV–III–HD-400 spectrometer operating at 400.13 MHz. ^1^H NMR spectra for all other compounds were recorded at room temperature on a 400 MHz Bruker AVANCE spectrometer or a 500 MHz Bruker AVANCE spectrometer locked on the signal of deuterated solvents. All chemical shifts are reported relative to tetramethylsilane using the chosen deuterated solvent as a standard. ^17^O NMR spectra were collected at room temperature on a Bruker AV–III–HD-400 spectrometer (at 54.2 MHz) or a 500 MHz Bruker AVANCE spectrometer (at 67.8 MHz), with the spectrometer locked on the signal of the deuterated solvents and all chemical shifts given relative to an external standard of D_2_O. Cyclic voltammetry (CV) was performed using a three-electrode setup inside a negative-pressure glovebox (MBraun UniLab, USA) using a CH Instrument 620E potentiostat or a Bio-Logic SP 150 potentiostat/galvanostat. The concentration of the cluster and the supporting electrolyte (TBAPF_6_) were kept at 1 mM and 100 mM respectively throughout all measurements. CVs were recorded using a 3 mm diameter glassy carbon working electrode (CH Instruments, USA), a Pt wire auxiliary electrode (CH Instruments, USA), a silver wire quasi-reference electrode for **6-Np(W**_**4**_**Mo)**_**2**_ and a Ag/Ag^+^ nonaqueous reference electrode with 0.01 M AgNO_3_ in 0.1 M TBAPF_6_ in acetonitrile (BASi, USA) for all other compounds. Ferrocene was used as an internal standard after completion of the measurements, and potentials were referenced versus the Fc^+/0^ couple. For **6-Np(W**_**4**_**Mo)**_**2**_, electronic absorption measurements were recorded inside a negative pressure argon Drybox at room temperature in anhydrous MeCN in a sealed 1 cm quartz cuvette using a JASCO V-770 UV–vis–NIR spectrophotometer equipped with a fiber optic stage and sample holder. For all other compounds, electronic absorption measurements were recorded at room temperature in anhydrous MeCN or DCM in sealed 1 cm quartz cuvettes using an Agilent Cary 6000i UV–vis–NIR spectrophotometer. Elemental analysis data were obtained from the Elemental Analysis Facility at the University of Rochester. Microanalysis samples were weighed with a PerkinElmer model AD6000 autobalance, and their compositions were determined with a PerkinElmer 2400 series II analyzer. Air-sensitive samples were handled in a VAC Atmospheres glovebox.

### X-ray Crystallography

For **6-Np(W**_**4**_**Mo)**_**2**_, single crystals suitable for X-ray diffraction were coated with poly(isobutylene) oil in the glovebox and quickly transferred to the goniometer head of a Bruker Quest diffractometer with a fixed chi angle, a sealed tube fine focus X-ray tube, single crystal curved graphite incident beam monochromator, a Photon II area detector and an Oxford Cryosystems low temperature device. Examination and data collection were performed with Mo Kα radiation (λ = 0.71073 Å) at 150 K. For all other compounds, crystals were placed onto a nylon loop and mounted on a Rigaku XtaLAB Synergy-S DualFlex diffractometer equipped with a HyPix-6000HE HPC area detector for data collection at 100.00(10) K. A preliminary set of cell constants and an orientation matrix were calculated from a small sampling of reflections.^46^ A short pre-experiment was run, from which an optimal data collection strategy was determined. The full data collection was carried out using a PhotonJet (Mo) X-ray source. After the intensity data were corrected for absorption, the final cell constants were calculated from the xyz centroids of the strong reflections from the actual data collection after integration.^46^ The structure was solved using SHELXT or isomorphic replacement and refined using SHELXL.^47,48^ Most or all non-hydrogen atoms were assigned from the solution. Full-matrix least-squares/difference Fourier cycles were performed which located any remaining non-hydrogen atoms. All non-hydrogen atoms were refined with anisotropic displacement parameters. All hydrogen atoms were placed in ideal positions and refined as riding atoms with relative isotropic displacement parameters.

### Computational Methods

In order to investigate the electronic structure and origins of the experimentally observed electronic transitions, quantum mechanical simulations were carried using the Gaussian16 software package for the closed-shell, experimentally reported materials described both previously and in this work.^32,33,49^ The starting molecular structures used to initialize the isolated-molecule simulations were obtained from the experimental single-crystal X-ray diffraction experiments. The calculations reported in the main text made use of the all-electron 6-311G(2d,2f) basis set to model the atomic wave functions for the light elements (C, H, O, N, Na),^50−52^ while the heavy elements used the Stuttgart relativistic small core (RSC) pseudopotentials^53−55^ for the core electrons (28 electrons for Zr and Mo, and 60 electrons for Hf, W, and Th), coupled with its associated basis set proposed by Martin^56^ for the remaining valence electrons. Calculations utilized the B3LYP^57−59^ level of the theory and numerical integrations were evaluated in the ultrafine grid defined in Gaussian16 using convergence criteria of d*E* < 10^–6^ hartree in the density matrix. The absolute energy change is not used to evaluate convergence; however, our choice of parameters leads to a change in the energy d*E* no larger than 10^–9^ hartree. The experimental electronic absorption spectra were obtained in solution (acetonitrile) and therefore, the influence of the solvent on the electronic structure was modeled through the Polarizable Continuum Model (PCM).^60^

In addition to the production simulations reported here, a wide range of basis sets and functionals were explored to investigate the best set of theoretical parameters for these calculations—those results are available in the Supporting Information. Once fully optimized structures were obtained, vibrational analyses were performed to ensure that the structures were located at a minimum on the potential energy surface; none of the optimized structures reported in this work generated negative-frequency modes.

To simulate the electronic transitions, and their absorption coefficients, time-domain DFT (TD-DFT)^61−64^ simulations were subsequently performed on the fully optimized structures. The calculations utilized 200 roots, with 100 for singlet excited states, and 100 for the triplet excited states. Visualization of the molecular orbitals was performed using the cubegen utility in the Gaussian16 code, using an isosurface value of 0.2 and 12 points/bohr.

Reduction potentials (*E*°_1/2_) were calculated using the differences in Gibbs free energy between the oxidized and reduced forms of **1-NaMo**_**5**_ and **1-NaW**_**4**_**Mo**, as shown in the equations below; where *G*(Ox) is the Gibbs free energy of the oxidized species, *G*(red) is the Gibbs free energy of the 1e^–^ reduced species and *F* is Faraday’s constant.^65−68^
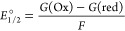

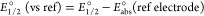

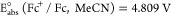


## Results & Discussion

### Synthesis of Sandwich-Type Complexes

Villanneau and co-workers have previously reported the synthesis of the tungsten-containing sandwich complex, (TBA)_3_[Bi{W_4_O_13_(OMe)_4_MoNO}_2_], following reaction of (TBA)_2_[W_4_O_13_(OMe)_4_MoNO][Na(MeOH)] (**1-NaW**_**4**_**Mo**) with BiCl_3_ in methanol.^25^ However, compared to the synthesis of the all molybdenum species, (TBA)_2_[Mo_5_O_13_(OMe)_4_NO][Na(MeOH)] (**1-NaMo**_**5**_),^24^ the preparation of **1-NaW**_**4**_**Mo** is long and produces only small amounts of the lacunary assembly. The formation of **1-NaW**_**4**_**Mo** first requires synthesis of (TBA)_3_[W_5_O_18_MoNO] from (TBA)_2_[WO_4_] and Mo(acac)_2_{CH_3_C(NH_2_)NO}NO.^26,40,41^ Additionally, the formation of Mo(acac)_2_{CH_3_C(NH_2_)NO}NO acts as a bottleneck for the overall reaction, isolated in only a 30% yield after 3 days. This limits the overall yield of (TBA)_3_[W_5_O_18_MoNO] to ca. 17%, which is then treated with four equivalents of NaOMe in methanol to give **1-NaW**_**4**_**Mo** in an overall yield of ca. 5%.^26,40,41^

**Scheme 1:**
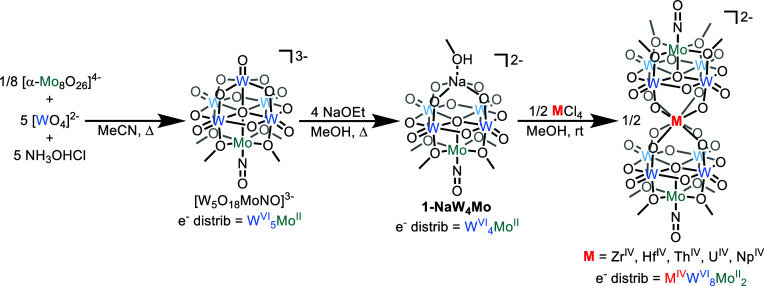
New Synthetic Route for the Synthesis of **1-NaW**_**4**_**Mo** Which Can Be Directly Converted to Sandwich-Type Complexes by Treatment with Metal Chlorides

In an attempt to improve the overall efficiency of the preparation of **1-NaW**_**4**_**Mo**, we set out to find an alternative, simpler and/or higher yielding, synthesis of (TBA)_3_[W_5_O_18_MoNO]. The corresponding all molybdenum complex, (TBA)_3_[Mo_6_O_18_NO], has been synthesized by acidification of acetonitrile solutions of (TBA)_2_[Mo_2_O_7_] with hydroxylamine hydrochloride (NH_3_OHCl), simultaneously acting as a source of protons and hydroxylamine required for the formation of the molybdenum nitrosyl unit.^26^ Inspired by this approach, we reasoned that acidification of a mixture of the appropriate ratio of soluble tungstate and molybdate building blocks would allow direct assembly of (TBA)_3_[W_5_O_18_MoNO]. A mixture of (TBA)_4_[α-Mo_8_O_26_], (TBA)_2_[WO_4_], and NH_3_OHCl were combined in a 1:40:40 ratio in acetonitrile (Scheme 1). Refluxing for 4 h leads to the formation of a brown solution. Following workup, direct crystallization of green product, identified as (TBA)_3_[W_5_O_18_MoNO], from the reaction solution is possible (yield = 59%). Infrared (IR) spectroscopy confirmed the formation of the mixed-metal nitrosyl complex, which features a characteristic ν(NO) stretch of the {Mo–NO}^4^ unit at around 1580 cm^–1^ (observed at 1530 cm^–1^ for (TBA)_3_[W_6_O_18_NO]).^26^ Performing the same reaction on a smaller scale in the presence of ^17^O enriched water allows direct isotopic labeling of the product; analysis of the ^17^O NMR spectrum of the product further confirmed the formation of (TBA)_3_[W_5_O_18_MoNO] (Figure S10). This approach represents a significant improvement over that reported previously, not only resulting in a higher overall yield, but rendering the preparation of the assembly amenable to large-scale production (>8 g per synthesis).

With (TBA)_3_[W_5_O_18_MoNO] in hand, attention was turned to the synthesis of **1-NaW**_**4**_**Mo**. Unfortunately, it proved difficult to make similar improvements to the conversion of (TBA)_3_[W_5_O_18_MoNO] to **1-NaW**_**4**_**Mo**. The instability of **1-NaW**_**4**_**Mo** in solvents other than methanol removes the option to vary solvent. Replacement of NaOMe with other bases (e.g., NaOEt, NaO^*t*^Bu or NaOH) led to no noticeable improvement in yield. We therefore opted to employ methods similar to those originally used by Villanneau and co-workers for the conversion of (TBA)_3_[W_5_O_18_MoNO] to **1-NaW**_**4**_**Mo** (Scheme 1, see Experimental Section for details).

Previously we have reported efficient formation of metal(IV) centered sandwich-type complexes with the general formula (TBA)_2_[M{Mo_5_O_13_(OMe)_4_NO}_2_] (M = Zr, Hf, Th, U, and Np) by reaction of **1-NaMo**_**5**_ with the corresponding metal(IV) chloride in methanol.^32,33^ In order to extend the series to the corresponding tungsten-containing compounds, **1-NaW**_**4**_**Mo** was treated with just over half an equivalent of a metal(IV) chloride (M = Zr, Hf, Th, U, and Np) in methanol (Scheme 1). Immediate precipitation of the product is observed, with the color depending on the identity of the metal(IV) salt employed (see Experimental Section). Following workup, analysis of both the chemical shifts and integrals of the major signals present in the ^1^H NMR spectra of the obtained materials supports successful formation of sandwich-type complexes with the general formula (TBA)_2_[M{W_4_O_13_(OMe)_4_MoNO}_2_] (M = Zr, Hf, Th, U, and Np), referred to as **2-Zr(W**_**4**_**Mo)**_**2**_, **3-Hf(W**_**4**_**Mo)**_**2**_, **4-Th(W**_**4**_**Mo)**_**2**_, **5-U(W**_**4**_**Mo)**_**2**_, and **6-Np(W**_**4**_**Mo)**_**2**_, respectively (Figures S4–S8). In general, the spectra feature five resonances, four of which are assigned to the TBA cations and one that is attributed to the -OMe groups of the polyoxoalkoxide unit. The chemical shifts of the major resonances in the ^1^H NMR spectrum of paramagnetic uranium-containing derivative, **5-U(IV)(W**_**4**_**Mo)**_**2**_, are significantly different to those observed for any of the other complexes synthesized. The signals assigned to the TBA cations are shifted upfield, occurring between −2 and −5 ppm, while the resonance assigned to the –OMe groups is observed at 10.02 ppm, shifted downfield by ∼6 ppm from its diamagnetic congeners (Figure S7). This is consistent with the behavior previously observed for the all-molybdenum derivative, (TBA)_2_[U(IV){Mo_5_O_13_(OMe)_4_NO}_2_]. Conversely, analysis of the paramagnetic Np(IV) (5f^3^) centered complex, **6-Np(W**_**4**_**Mo)**_**2**_, reveals an ^1^H NMR spectrum almost identical to its diamagnetic congeners (Figure S8), exemplifying how the chemical environment of the protons present in the -OMe groups can vary drastically as the electron occupancy of the 5f orbitals is changed. This behavior was interrogated further using ^17^O NMR spectroscopy. Details of the synthetic methodology used to produce ^17^O enriched analogues of all the complexes discussed above is given in the Experimental Section, while the obtained spectra and a discussion of the assignments is given in the Supporting Information (Section S2).

**Figure 2:**
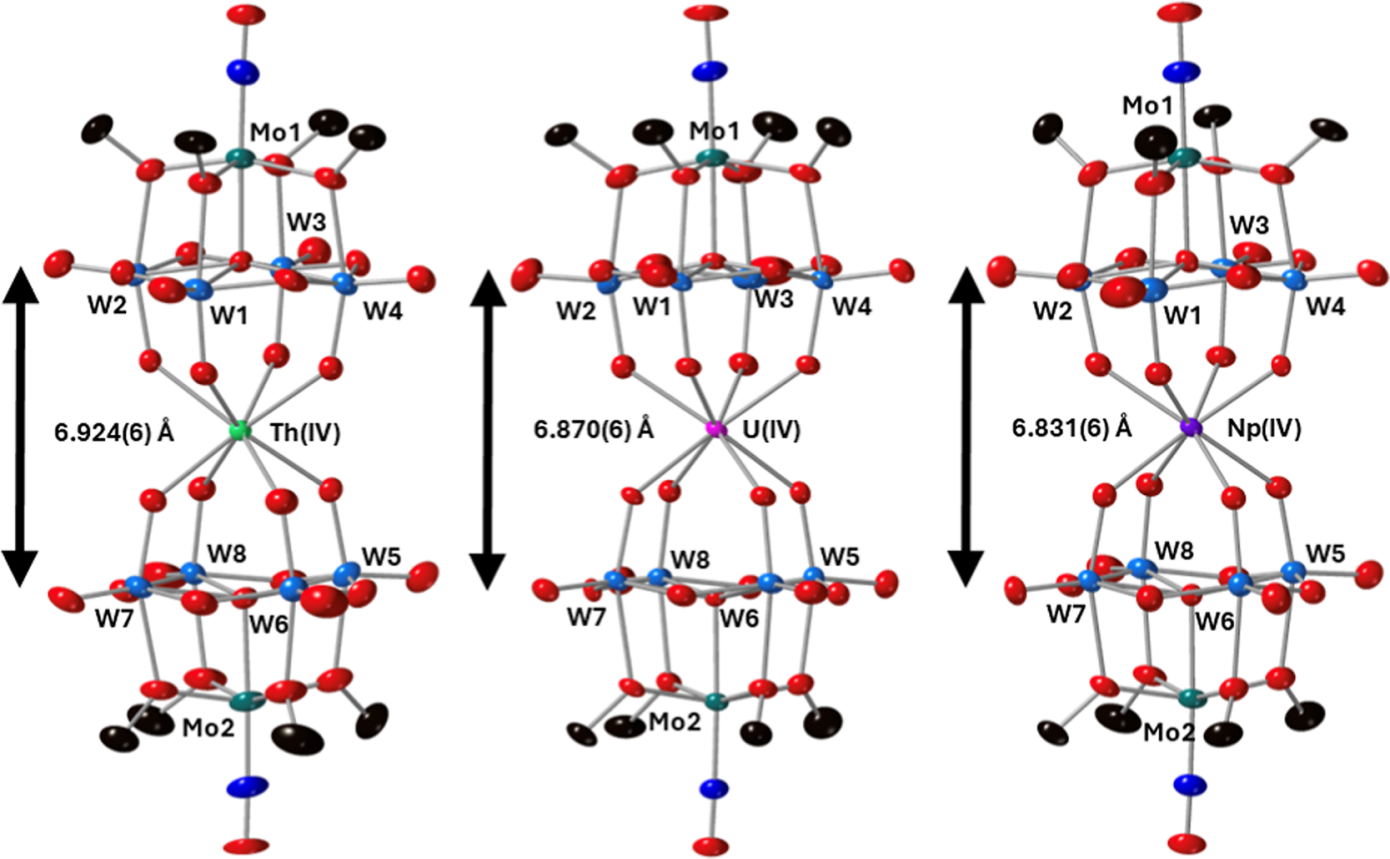
SCXRD structures of **4-Th(W**_**4**_**Mo)**_**2**_, **5-U(W**_**4**_**Mo)**_**2**_, and **6-Np(W**_**4**_**Mo)**_**2**_ with probability ellipsoids set at 50%. The terabutylammonium cations, protons, and some disorder has been removed for clarity.

Solid state structural characterization of the actinide centered derivatives was pursued to gain insights into how incorporation of tungsten into the polyoxoalkoxide ligands affects the local coordination environment of the complexes. Single crystals of **4-Th(W**_**4**_**Mo)**_**2**_, **5-U(W**_**4**_**Mo)**_**2**_ and **6-Np(W**_**4**_**Mo)**_**2**_ were grown by vapor diffusion of diethyl ether into saturated solutions of the complexes dissolved in acetonitrile. Analysis of these crystals by SCXRD gave, after refinement of the data, the structures shown in Figure 2.

Overall, the structures confirm the formation of sandwich-type complexes featuring two {W_4_Mo} units either side of a central eight coordinate actinide center. The actinide center has an approximately square antiprismatic coordination geometry which is consistent with previously reported examples of An(IV)(L)_2_ complexes, where L = [Mo_5_O_13_(OMe)_4_NO]^3–^, [W_5_O_18_]^6–^, [XW_11_O_39_]^*n*−^, [PMo_11_O_39_]^7–^ and [P_2_W_17_O_61_]^11–^.^1,6,23,32,33,69,70^ Examining the lengths of the An-O bonds reveals average Th–O bond lengths of ca. 2.41 Å, U–O bond lengths of ca. 2.36 Å, and average Np–O bond lengths of ca. 2.35 Å. The decreasing bond length can be rationalized by the decreasing effective ionic radius of the An(IV) center as you move across the f-block: Th (1.05 Å) to U (1.00 Å) and finally to Np (0.98 Å).^71^ The An–O bond lengths are close to identical to those reported for the corresponding all-molybdenum complexes (i.e., (TBA)_2_[An{Mo_5_O_13_(OMe)_4_NO}_2_]), indicating that swapping the high-valent framework metals in the polyoxoalkoxide ligands has almost no impact on the local coordination environment of the actinide. This is in line with the literature where An–O bond lengths change very little regardless of the structure, charge, or framework metal of the POM used to form the sandwich-type complex.^15^ The spacing between the two-halves of the sandwich-type complex, approximated by the μ_5_-O-μ_5_-O distance, shown in Figure 2, decreases in the order Th > U > Np. This, again, follows the decrease in the ionic radius of the actinide center, and the decrease in An–O bond lengths, as we move across the f-block.^71^

### Optical Properties and Electronic Structure

**Figure 3:**
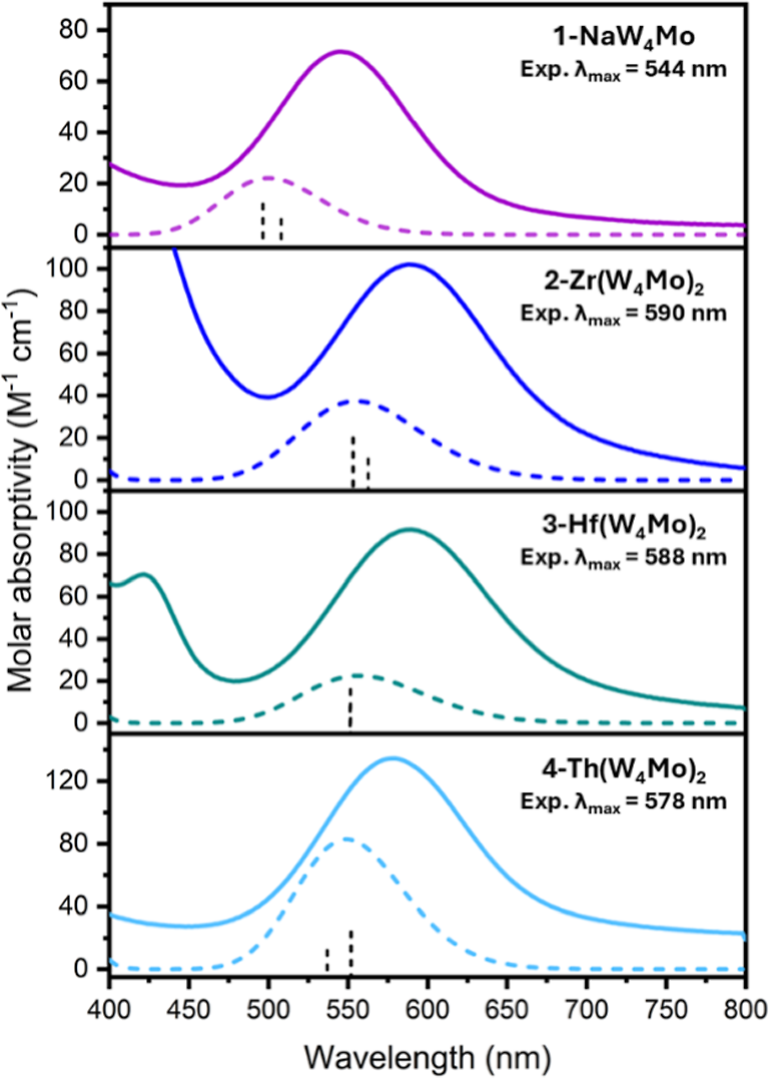
Experimental electronic absorption spectra (bold lines) of **1-NaW**_**4**_**Mo**, **2-Zr(W**_**4**_**Mo)**_**2**_, **3-Hf(W**_**4**_**Mo)**_**2**_, and **4-Th(W**_**4**_**Mo)**_**2**_ collected in MeCN at 21 °C. Simulated spectra obtained from TD-DFT calculations (dashed lines) are also shown. The vertical dashed bars under the simulated spectra correspond to the dimensionless oscillator strength from which the spectra were convoluted, assuming a Gaussian shape.

With a complete series of tungsten-containing M(IV) centered sandwich-type complexes in hand, we next investigated how variation of the framework metal impacts the electronic structure of the assemblies. We first examined the optical properties of the series using electronic absorption spectroscopy. The UV–vis spectra of the diamagnetic compounds (i.e., **1-NaW**_**4**_**Mo**, **2-Zr(W**_**4**_**Mo)**_**2**_, **3-Hf(W**_**4**_**Mo)**_**2**_, and **4-Th(W**_**4**_**Mo)**_**2**_) are shown in Figure 3. The obtained spectrum of **1-NaW**_**4**_**Mo** features a single broad absorption at 544 nm (ε = 72 mol^–1^ dm^3^ cm^–1^), matching closely to the spectrum previously reported.^25^ This spectrum is also similar to that of the all-molybdenum analogue, **1-NaMo**_**5**_.^24^ The broad absorption in these spectra was attributed to a d_*xy*_ ← d_*xz*_,d_*yz*_ transition of the {Mo–NO}^4^ unit.^24,25^ The spectra of the sandwich complexes **2-Zr(W**_**4**_**Mo)**_**2**_, **3-Hf(W**_**4**_**Mo)**_**2**_, and **4-Th(W**_**4**_**Mo)**_**2**_ are very similar to that of **1-NaW**_**4**_**Mo** suggesting the oxidation state distribution within the {W_4_Mo} units (i.e., 4 x W(VI) and 1 x Mo(II)) is retained upon binding. The spectra feature the same major absorption, though it is red-shifted (λ_max_ = 578–590 nm). This mirrors the behavior that was previously observed upon formation of the corresponding all molybdenum sandwich complexes (i.e., (TBA)_2_[M{Mo_5_O_13_(OMe)_4_NO}_2_], where M = Zr, Hf, and Th referred to as **2-Zr(Mo**_**5**_**)**_**2**_, **3-Hf(Mo**_**5**_**)**_**2**_, and **4-Th(Mo**_**5**_**)**_**2**_), where the λ_max_ value appeared to increase with the Lewis acidity of the central heterometal.^33^

**Figure 4:**
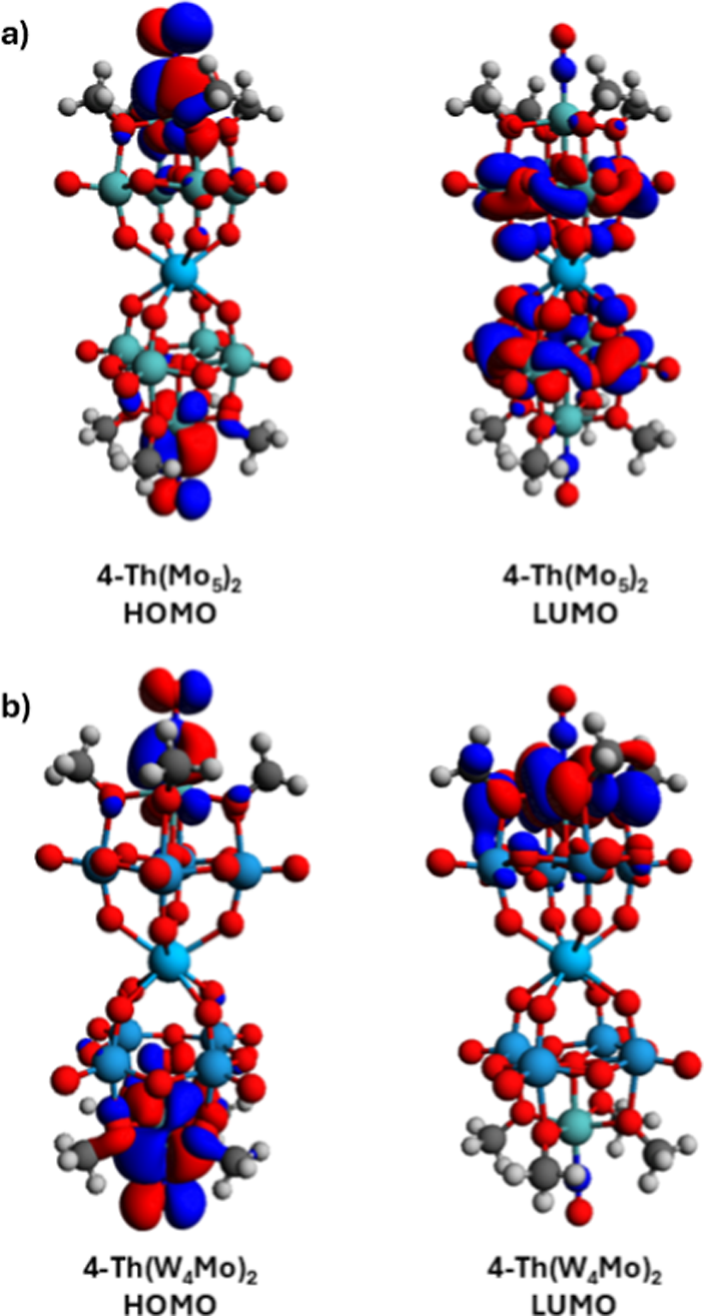
HOMO and LUMO orbitals of (a) **4-Th(Mo**_**5**_**)**_**2**_ and (b) **4-Th(W**_**4**_**Mo)**_**2**_ obtained from DFT calculations as a representative example of the frontier orbitals of the diamagnetic sandwich-type complexes discussed in this work.

To gain insights into the electronic structure of the complexes and confirm the assignment of the transition responsible for the major absorption present in the UV–vis spectra shown in Figure 3, DFT calculations were performed on **1-NaW**_**4**_**Mo**, **2-Zr(W**_**4**_**Mo)**_**2**_, **3-Hf(W**_**4**_**Mo)**_**2**_, **4-Th(W**_**4**_**Mo)**_**2**_, and their all-molybdenum analogues. First, the atomic positions were allowed to fully relax with no symmetry-related constraints, followed by a frequency calculation to ensure that the optimized structures represented a minimum on the potential energy surface. The wave functions obtained from the fully optimized structure were used to identify the energies and positions of the frontier molecular orbitals. As a representative example, the HOMOs and LUMOs of **4-Th(Mo**_**5**_**)**_**2**_ and **4-Th(W**_**4**_**Mo)**_**2**_ are shown in Figure 4, while the frontier orbitals of **1-NaW**_**4**_**Mo**, **1-NaMo**_**5**_, **2-Zr(W**_**4**_**Mo)**_**2**_, **2-Zr(Mo**_**5**_**)**_**2**_, **3-Hf(W**_**4**_**Mo)**_**2**_, and **3-Hf(Mo**_**5**_**)**_**2**_ are given in Figures S37–S48. The HOMO, HOMO–1, HOMO–2, and HOMO–3 were localized on the {Mo–NO}^4^ moieties of the polyoxoalkoxide units, regardless of the identity of the central M(IV) ion present or the framework metal (i.e., Mo vs W; Figures S37–S50). This supports the assignment of {Mo–NO}^4^ for each metal-nitrosyl unit, with the eight metal electrons involved in bonding populating the four highest energy filled molecular orbitals. This differs from typical POM systems, where the HOMO is localized on the bridging oxo moieties and thus primarily has O 2p character.^34^ Similar orbitals, which incorporate O 2p character, are instead found at lower energies in the sandwich-type complexes studied here (HOMO–4 and below). These results are consistent with previous DFT calculations on the two-electron reduced polyoxoalkoxide [Mo_10_O_25_(OMe)_6_(NO)]^6–^, and demonstrate the drastic change that incorporation of the {Mo–NO}^4^ unit has on the electronic structure of the assembly.^72^

Unlike the HOMOs, which are consistent across all of the complexes studied, there are striking differences in the nature of the LUMO and LUMO+1 upon substitution of the equatorial Mo(VI) centers of the polyoxoalkoxide ligands for W(VI). The LUMO and LUMO+1 of **2-Zr(Mo**_**5**_**)**_**2**_, **3-Hf(Mo**_**5**_**)**_**2**_, and **4-Th(Mo**_**5**_**)**_**2**_ are primarily (de)localized on the equatorial planes of the polyoxolkoxide units (Figures 4a, S39, S41, and S43). This is typical for POMs, and suggests that reduction of these complexes would lead to the addition of electrons delocalized across the Mo(VI) centers.^72^ However, upon substitution of Mo for W, the LUMO and LUMO+1 of **2-Zr(W**_**4**_**Mo)**_**2**_, **3-Hf(W**_**4**_**Mo)**_**2**_, and **4-Th(W**_**4**_**Mo)**_**2**_ are pushed toward the tips of the sandwich-type complexes, essentially localized on the axial Mo centers (Figures 4b, S46, S48, and S50). This is likely to maximize the Mo 4d contribution to the LUMO/LUMO+1 which are lower in energy than the W 5d orbitals. The variation in the nature of the LUMO and LUMO+1 is likely to be a factor in any divergent properties between the M(Mo_5_)_2_ and M(W_4_Mo)_2_ sandwich-type complexes.

After obtaining optimized structures, TD-DFT calculations were used to simulate the electronic absorption spectra of the complexes (Figures 3, S53–S61). The TD-DFT simulations show excellent agreement with the experimental UV–vis spectra, indicating that the simulations are appropriately modeling the electronic structure of these complexes. In **4-Th(Mo**_**5**_**)**_**2,**_ the electronic transitions associated with the experimentally observed feature at 575 nm originate from occupied frontier orbitals (e.g., for Th, HOMO–3 through HOMO) primarily to two unoccupied orbitals, LUMO+6 and LUMO+7. The occupied orbitals involve electron density located on the {Mo–NO}^4^ moiety, while the unoccupied orbitals are similarly localized on the Mo–N interaction.^24^ This is similarly the case with transitions observed in the tungsten derivative, **4-Th(W**_**4**_**Mo)**_**2**_, which is not surprising given the similar experimental UV–vis spectra between the related compounds. This behavior is consistent across the full series of complexes for which TD-DFT calculations were performed with the only differences being the exact LUMOs involved in the transitions (see Supporting Information, Section S7).

**Figure 5:**
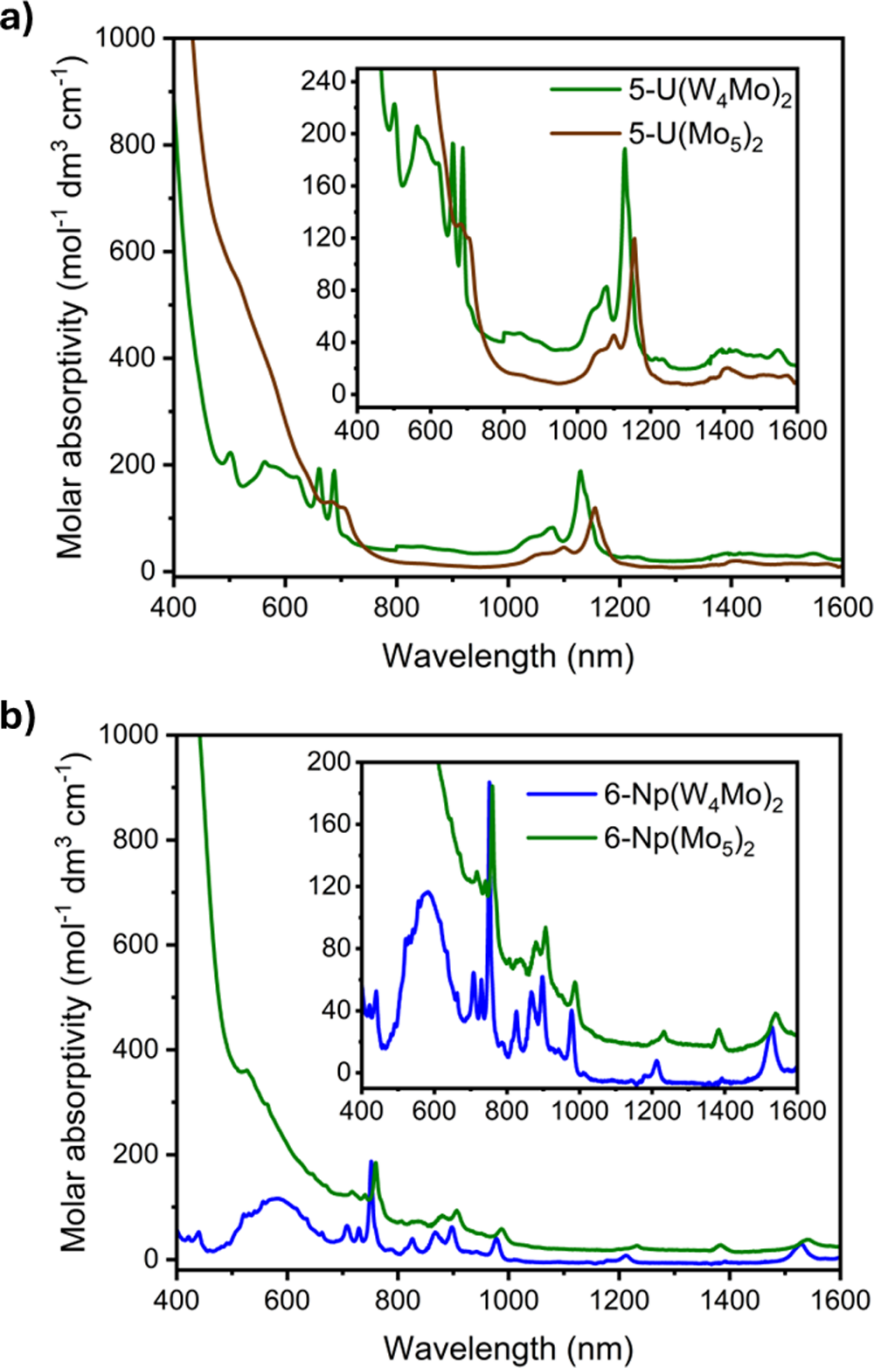
UV–vis–NIR spectra of (a) **5-U(W**_**4**_**Mo)**_**2**_ (green) and **5-U(Mo**_**5**_**)**_**2**_ (brown) and (b) **6-Np(W**_**4**_**Mo)**_**2**_ (blue) and **6-Np(Mo**_**5**_**)**_**2**_ (green). The spectra were collected in MeCN at 21 °C.

Analysis of the electronic absorption spectra of the sandwich-type complexes incorporating paramagnetic actinide centers, **5-U(W**_**4**_**Mo)**_**2**_ and **6-Np(W**_**4**_**Mo)**_**2**_, reveals drastic changes. Previously, we have reported UV–vis–NIR spectra of (TBA)_2_[U{Mo_5_O_13_(OMe)_4_NO}_2_] (**5-U(Mo**_**5**_**)**_**2**_) and (TBA)_2_[Np{Mo_5_O_13_(OMe)_4_NO}_2_] (**6-Np(Mo**_**5**_**)**_**2**_). These spectra are shown in Figure 5a (brown) and 5b (green). Alongside several low intensity peaks attributed to transitions between the partially filled f-orbitals, a broad, more intense, absorption was observed across the visible region. This was attributed to a MLCT from the actinide (U(IV) or Np(IV)) to the polyoxoalkoxide ligands (i.e., An(5f) → Mo(4d)), based on previous reports showing similar behavior in U(IV)/Np(IV) containing polyoxometalate complexes.^2,6,7,73,74^

As part of this study, the UV–vis–NIR spectra of **5-U(W**_**4**_**Mo)**_**2**_ and **6-Np(W**_**4**_**Mo)**_**2**_ were recorded and are shown in Figure 5a (green) and 5b (blue). Focusing on the spectrum of **5-U(W**_**4**_**Mo)**_**2**_ (Figure 5a), it is apparent that the intensity of the absorption associated with MLCT is reduced, suggesting that substitution of Mo for W in the polyoxoalkoxide ligands limits MLCT in **5-U(W**_**4**_**Mo)**_**2**_. This allows the visualization of additional peaks in the UV–vis–NIR spectrum of **5-U(W**_**4**_**Mo)**_**2**_. On top of the easily visible f–f transitions observed at 1130 nm (ε = 188 mol^–1^ dm^3^ cm^–1^) and 1080 nm (ε = 83 mol^–1^ dm^3^ cm^–1^), which were found at similar positions for **5-U(Mo**_**5**_**)**_**2**_, additional sharp f–f transitions are observed at 688 nm (ε = 189 mol^–1^ dm^3^ cm^–1^), 662 nm (ε = 192 mol^–1^ dm^3^ cm^–1^), and 502 nm (ε = 223 mol^–1^ dm^3^ cm^–1^). These absorptions are also accompanied by an additional broad feature at around 580–600 nm. This absorption is assigned to the same transition between the bonding orbitals of the{Mo–NO}^4^ unit and unoccupied orbitals of the polyoxoalkoxide groups.

Examining the spectrum of **6-Np(W**_**4**_**Mo)**_**2**_ (Figure 5b) reveals comparable behavior. In this case, virtually no Np → POM MLCT is observed in the visible region. This is in line with previous observations showing that intensity of Np(5f) → POM MLCT is consistently found to be lower than the intensity of U(5f) → POM MLCT in these complexes.^32^ This may be attributed to the reduction in the radial extension of the 5f orbitals when moving from U(IV) to Np(IV), which limits the interaction between the partially filled 5f orbitals and the polyoxoalkoxide LUMO.^75^ Inspecting shorter wavelengths (Figure S28) does show intense absorption at less than 360 nm in the UV–vis–NIR spectrum of **6-Np(W**_**4**_**Mo)**_**2**_. This theoretically could be assigned to Np → POM MLCT, meaning that the impact of incorporation of tungsten into the framework was to shift absorption associated with Np → POM MLCT to higher energies. However, intense absorption is also observed at less than 360 nm in the UV–vis spectra of **2-Zr(W**_**4**_**Mo)**_**2**_, **3-Hf(W**_**4**_**Mo)**_**2**_, and **4-Th(W**_**4**_**Mo)**_**2**_ (Figure S28). Since M(IV) → POM MLCT is not possible in these systems, it is more likely that these transitions are associated with O(2p) → Mo(4d) LMCT.^76,77^ This was confirmed for **2-Zr(W**_**4**_**Mo)**_**2**_, **3-Hf(W**_**4**_**Mo)**_**2**_, and **4-Th(W**_**4**_**Mo)**_**2**_ using TD-DFT (Figure S61), which showed that the intense absorptions observed at <360 nm (with the peak maxima at ca. 310 nm) are largely caused by transitions between lower lying HOMOs (with primarily O(2p) character) and the LUMO or LUMO+1 (Tables S14–S16). It is therefore sensible to assign the intense absorption observed at <360 nm in the spectrum **6-Np(W**_**4**_**Mo)**_**2**_ to the same process.

The lack of MLCT absorption at 400–700 nm in the spectrum of **6-Np(W**_**4**_**Mo)**_**2**_ allows observation of the broad peak assigned to the {Mo–NO}^4^ unit at 583 nm (ε = 117 mol^–1^ dm^3^ cm^–1^), occurring at an intermediate wavelength between that of **2-Zr(W**_**4**_**Mo)**_**2**_ (588 nm) and **4-Th(W**_**4**_**Mo)**_**2**_ (578 nm). The ionic radius of Np(IV) (0.98 Å) is slightly lower than that of Th(IV) (1.05 Å), and therefore Np(IV) is slightly more Lewis acidic. This means the intermediate position of the λ_max_ value of the absorption associated with the {Mo–NO}^4^ unit is consistent with the previously reported trend. The UV–vis-NIR spectrum of **6-Np(W**_**4**_**Mo)**_**2**_ also contains several sharp absorptions spanning from 700 to 1600 nm (see the Experimental Section for details on the exact wavelengths of these absorptions). These absorptions are in very similar positions to those previously observed in the UV–vis–NIR spectrum of **6-Np(Mo**_**5**_**)**_**2**_, and are assigned to Np(IV) f–f transitions, most of which have also been seen in the spectra of [Np(W_5_O_18_)_2_]^8–^ and [Np(BW_11_O_39_)(W_5_O_18_)]^11–^.^6^

The exact reason for the drastic reduction of MLCT in **5-U(W**_**4**_**Mo)**_**2**_ and **6-Np(W**_**4**_**Mo)**_**2**_ when compared to **5-U(Mo**_**5**_**)**_**2**_ and **6-Np(Mo**_**5**_**)**_**2**_ is not immediately apparent, given that most known examples of U(IV) and Np(IV) containing POTs that report UV–vis–NIR data show the presence of broad absorptions across the visible region (assigned to MLCT).^2,6,7,74^ As such, this is likely to be a more unique characteristic associated with the specific polyoxoalkoxides employed in this study. One possible explanation could be that the variation in the nature LUMOs discussed above for the diamagnetic sandwich-type complexes is responsible for the change in MLCT behavior upon replacement of Mo(VI) with W(VI). If LUMO/LUMO+1 of **5-U(Mo**_**5**_**)**_**2**_ and **6-Np(Mo**_**5**_**)**_**2**_ are localized around the equatorial plane of the polyoxoalkoxide ligands (as was seen for **2-Zr(Mo**_**5**_**)**_**2**_, **3-Hf(Mo**_**5**_**)**_**2**_, and **4-Th(Mo**_**5**_**)**_**2**_) then they are likely to be spatially close to the An 5f orbitals. This would serve to maximize orbital overlap and favor MLCT. However, if the LUMO/LUMO+1 are localized at the tips of the sandwich-type complexes in **5-U(W**_**4**_**Mo)**_**2**_ and **6-Np(W**_**4**_**Mo)**_**2**_, then this would maximize the distance between the An 5f orbitals and LUMO/LUMO+1, which would limit orbital overlap and disfavor MLCT.

### Examining Redox Properties

**Figure 6:**
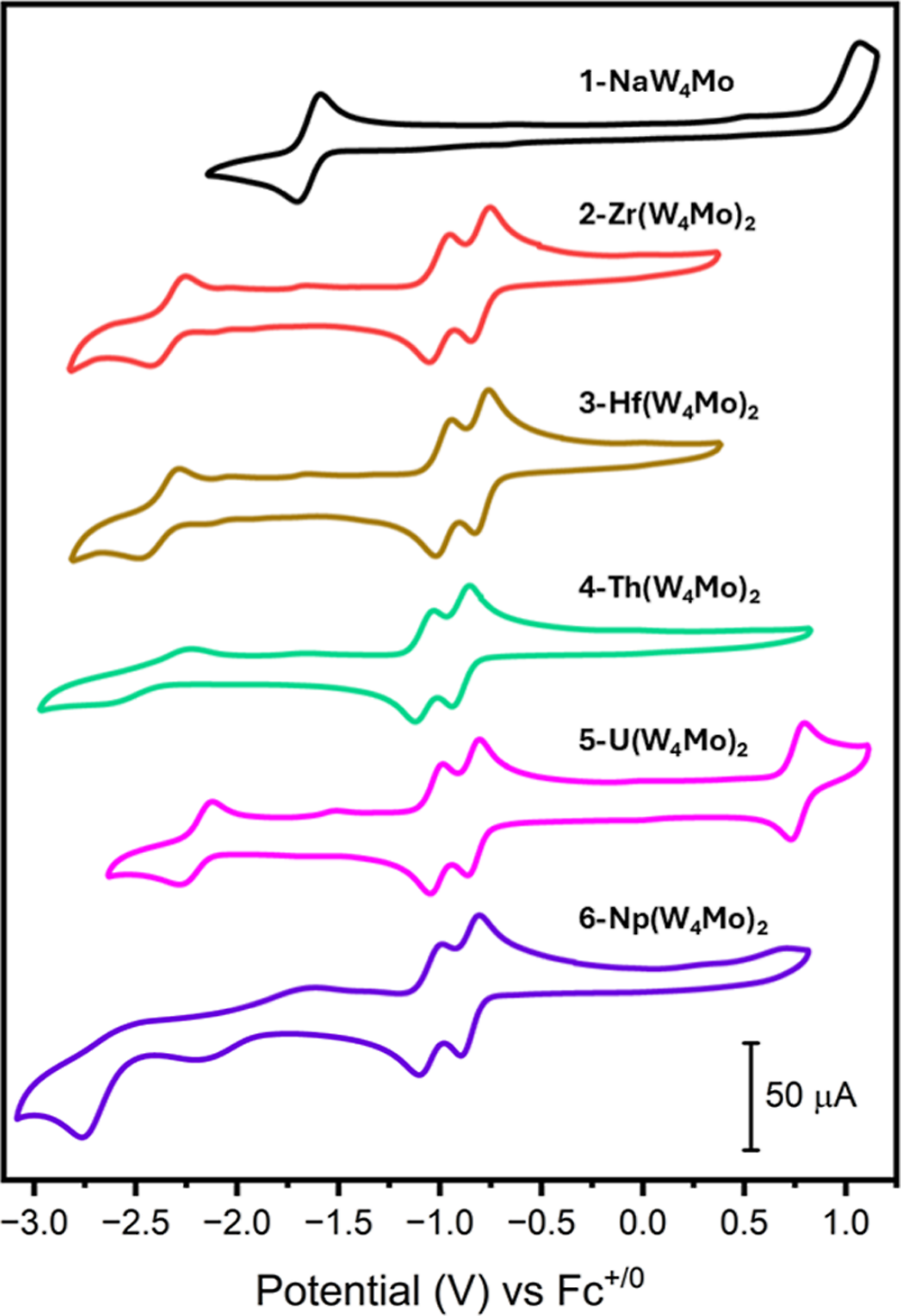
Cyclic voltammograms of **1-NaW**_**4**_**Mo** (black), **2-Zr(W**_**4**_**Mo)**_**2**_ (red), **3-Hf(W**_**4**_**Mo)**_**2**_ (brown), **4-Th(W**_**4**_**Mo)**_**2**_ (green), **5-U(W**_**4**_**Mo)**_**2**_ (pink), and **6-Np(W**_**4**_**Mo)**_**2**_ (purple). The data was acquired in MeCN with 0.1 M TBA(PF_6_) supporting electrolyte, 1 mM of cluster, and a scan rate of 200 mV s^–1^.

Following analysis of the optical properties, we next characterized the redox properties of the series via cyclic voltammetry (CV). The obtained CVs, recorded in 0.1 M TBA(PF_6_) in MeCN, are shown in Figure 6. Examining the voltammogram of **1-NaW**_**4**_**Mo** reveals the presence of a single reversible one electron reduction event (*E*_1/2_ = −1.65 V vs Fc^+/0^) and an irreversible oxidation (*E*_p_ = 1.16 V vs Fc^+/0^) event. The reversible reduction event is shifted anodically from that of **1-NaMo**_**5**_ (*E*_1/2_ = −1.88 V vs Fc^+/0^), implying that **1-NaW**_**4**_**Mo** is slightly easier to reduce than **1-NaMo**_**5**_. This observation is supported by DFT calculations where the calculated redox potentials, obtained from the difference in energy between the fully oxidized and one-electron reduced forms of the complexes (see Experimental Section for details), of **1-NaW**_**4**_**Mo** and **1-NaMo**_**5**_ were found to be −1.69 V and −1.94 V respectively. The irreversible oxidation of **1-NaW**_**4**_**Mo** has previously been assigned to oxidation of the {Mo–NO}^4^ unit, which is in line with DFT calculations (Figure S44) that show that the HOMO is localized on the Mo–NO unit. The corresponding oxidation of **1-NaMo**_**5**_ occurs at 0.76 V vs Fc^+/0^, indicating that the all-molybdenum derivative is more readily oxidized. This suggests that incorporation of tungsten into the framework stabilizes the HOMO, making loss of an electron from the {Mo–NO}^4^ unit more difficult. This is again supported by DFT calculations which show that the HOMO of **1-NaW**_**4**_**Mo** is lower in energy (−6.124 eV) than the HOMO of **1-NaMo**_**5**_ (−5.877 eV).

Examining the CVs of the sandwich-type complexes reveals that their redox properties are similar regardless of the identity of the M(IV) center. All the voltammograms feature two reversible one electron reductions between −0.75 V and −1.25 V vs Fc^+/0^. These reduction events can be attributed to the addition of a single electron to each {W_4_Mo} unit. The redox events are shifted anodically from the 1e^–^ reduction of **1-NaW**_**4**_**Mo**, which can be rationalized by the fact that the effective charge per {W_4_Mo} unit drops from 2– in **1-NaW**_**4**_**Mo** to 1– in the sandwich-type complexes. This reduction in charge should allow reduction to occur more readily.

The electrochemical behavior of the all-molybdenum analogues of the sandwich-type complexes (i.e., (TBA)_2_[M{Mo_5_O_13_(OMe)_4_NO}_2_], where M = Zr, Hf, Th, U, and Np) is very different to that shown in Figure 6 (Figure S36). First, the potential difference between the first two reduction events is larger, ranging from 0.29 to 0.56 V compared to an average of 0.19 V for the sandwich-type complexes shown in Figure 6. Second, the electrochemical behavior of the compounds is much more sensitive to the identity of the M(IV) ion present at the center of the sandwich-type complex, with the actinide centered derivatives (i.e., **4-Th(Mo**_**5**_**)**_**2**_, **5-U(Mo**_**5**_**)**_**2**_, and **6-Np(Mo**_**5**_**)**_**2**_) showing four reversible reduction events, compared to only two for the transition metal-centered derivatives (i.e., **2-Zr(Mo**_**5**_**)**_**2**_ and 3**-Hf(Mo**_**5**_**)**_**2**_). The change in redox behavior upon moving from sandwich-type complexes bearing [Mo_5_O_13_(OMe)_4_NO]^3–^ ligands to those bearing [W_4_O_13_(OMe)_4_MoNO]^3–^ ligands can be rationalized by considering the change in the nature of the LUMOs (and LUMO+1s) of the series of complexes. The LUMO and LUMO+1 are very close in energy, with DFT calculations showing these orbitals to be practically degenerate in **2-Zr(W**_**4**_**Mo)**_**2**_ and **3-Hf(W**_**4**_**Mo)**_**2**_ and separated by ca. 0.5 kcal/mol in **4-Th(W**_**4**_**Mo)**_**2**_. This means that the first two reduction events can be assigned to addition of a single electron to each polyoxoalkoxide ligand of the sandwich-type complex. As was previously discussed, the LUMO and LUMO+1 of the M(W_4_Mo)_2_ systems are pushed toward the tips of the complex (Figures S44–S50), likely to maximize the Mo 4d contribution to the LUMO.^78^ This maximizes the spatial separation between the LUMO and LUMO+1, meaning that adding electron density to the LUMO (i.e., one electron reduction) should have a limited impact on the energy of the LUMO+1. This serves to electronically decouple the two orbitals and explains the very small difference of the potentials of the first and second reduction events. This contrasts the picture in the M(Mo_5_)_2_ systems, where the LUMO and LUMO+1 are largely localized around the equatorial planes of the {Mo_5_} units and spread toward the central heterometal. It therefore makes sense that populating the LUMO, which is spatially very close to the LUMO+1, will have more of an impact on the energy of the LUMO+1 and therefore the difference in potential between the first and second reduction events is larger for these complexes. Furthermore, localization of the LUMO and LUMO+1 closer to the central heterometal in the M(Mo_5_)_2_ systems explains the increased sensitivity of the reduction chemistry to the nature of the heterometal.

The sandwich-type complexes also show an additional reduction event at much lower potentials; however, the exact position and reversibility of this process changes depending on the specific M(IV) cation present in the sandwich complex. It was also noted that this wave was often difficult to resolve in crude samples but was typically more pronounced after recrystallization. For **2-Zr(W**_**4**_**Mo)**_**2**_, **3-Hf(W**_**4**_**Mo)**_**2**_, **4-Th(W**_**4**_**Mo)**_**2**_, and **5-U(W**_**4**_**Mo)**_**2**_, this process appears to be reversible, though the wave for **4-Th(W**_**4**_**Mo)**_**2**_ is significantly broader suggesting more sluggish electron transfer kinetics, and is likely caused by the addition of a second electron to one of the {W_4_Mo} units of the sandwich-type complexes. Given that the LUMO+2 and above are primarily localized on the W(VI) centers of the {W_4_Mo} units, it is likely that this additional reduction event is associated with a W(VI) → W(V) reduction. This assignment was supported by DFT calculations. The structure of three electron reduced **4-Th(W**_**4**_**Mo)**_**2**_ (i.e., [Th{W_4_O_13_(OMe)_4_MoNO}_2_]^5–^) was optimized as both a doublet and quartet. The two spin multiplicities were found to have remarkably similar energies and in both cases the singly occupied HOMO (HOMO_α_), which is representative of where the added electron density gained upon the third reduction is localized, was found to have primarily W(5d) character (Figures S51 and S52). The large difference in energy between this reduction event and the previous two is consistent with the relatively large difference in energy between the HOMO_α_ and the next two highest energy singly occupied orbitals (which are localized on the molybdenum centers).

The third reduction event is less well resolved in the CV of **6-Np(W**_**4**_**Mo)**_**2**_ (Figure 6, purple), which may be because it was not possible to use recrystallized material for electrochemical experiments as syntheses could only be performed on small scales. The CV of crude **6-Np(W**_**4**_**Mo)**_**2**_ shows a broad quasi-reversible wave centered at approximately −1.91 V vs Fc^+/0^ (with a maximum at −1.61 V and minimum at −2.21 V). This wave has a relatively low intensity compared to the third reduction events in **Zr(W**_**4**_**Mo)**_**2**_, **3-Hf(W**_**4**_**Mo)**_**2**_, and **5-U(W**_**4**_**Mo)**_**2**_, but it may be that the broadness of the wave is the major contributor to lack of intensity, with a similar effect present in the voltammogram of **4-Th(W**_**4**_**Mo)**_**2**_. The combination of (1) the poor reversibility of this wave, (2) the fact that it is observed at more positive potentials than for all the other sandwich-type complexes, and (3) the presence of an additional irreversible event at −2.77 V vs Fc^+/0^, shows the uniqueness of the redox properties of **6-Np(W**_**4**_**Mo)**_**2**_. Interestingly, the redox properties of the previously reported all-molybdenum analogue, **6-Np(Mo**_**5**_**)**_**2**_, were also different from the other actinide centered complexes studied (Figure S36). During that study, our group found that the waves in the CV corresponding third and fourth reduction events appear much broader in **6-Np(Mo**_**5**_**)**_**2**_ than in **5-U(Mo**_**5**_**)**_**2**_. One possible cause of the consistent deviation in the behavior of the Np(IV)-containing compounds may be that we can start to “build up” Np(III) character under these highly reducing conditions. The ability to delocalize the added electrons across both the {W_4_Mo} and the An(IV) center should stabilize these highly reduced states, which may explain the anodic shift in the potential of the third reduction event for **6-Np(W**_**4**_**Mo)**_**2**_ (and to a lesser extent **5-U(W**_**4**_**Mo)**_**2**_). However, our group has previously shown that when a M(III) is present at the center of the all-molybdenum sandwich complex (i.e., [M(III){Mo_5_O_13_(OMe)_4_NO}_2_]^3–^) the redox properties are very different. In this case, the highly reversible electrochemistry displayed by the M(IV) sandwich complexes is instead replaced by largely irreversible reduction events.^33^ Therefore, if upon addition of three electrons to **6-Np(W**_**4**_**Mo)**_**2**_, a considerable portion of that additional spin density is localized at the relatively more reducible neptunium center^39,79−82^ we would expect subsequent reduction processes, accessed by scanning to more negative potentials, to be irreversible (Figure 6). The inability for the other M(IV) centers studied (which are all more difficult to reduce than neptunium) to support the additional electron density gained upon reduction would prevent the formation of any M(III) character and explain the absence of the irreversible reduction event.

### One Electron Oxidation of **5-U(W**_**4**_**Mo)**_**2**_

The CV of **5-U(W**_**4**_**Mo)**_**2**_ (Figure 6, pink) shows an additional reversible oxidation process at +0.76 V vs Fc^+/0^. A similar event was observed in CV of **5-U(Mo**_**5**_**)**_**2**_ (+0.74 V vs Fc^+/0^) and was found to correspond to reversible oxidation of U(IV) to U(V).^33^ Previously, chemical oxidation of **5-U(Mo**_**5**_**)**_**2**_ by [NO][PF_6_] was used to access the U(V) centered species, which represented the first example of a U(V) centered POM complex to be fully characterized.^33^ Given the U(V)/U(IV) redox couple of **5-U(W**_**4**_**Mo)**_**2**_ was observed at a close to identical potential to that of **5-U(Mo**_**5**_**)**_**2**_, it was reasoned that the oxidation of **5-U(W**_**4**_**Mo)**_**2**_ with [NO][PF_6_] would similarly allow isolation of the U(V)-containing species.

A green solution of **5-U(W**_**4**_**Mo)**_**2**_ in acetonitrile was treated with an excess (10 equiv) of [NO][PF_6_], resulting in the immediate formation a brown suspension. Following workup (see Experimental Section for details), the crude product was analyzed electrochemically (0.1 M TBA(PF_6_) in dichloromethane), which showed a very similar voltammogram to that of **5-U(W**_**4**_**Mo)**_**2**_ (Figure S35). Notably, the OCP of the product is observed at 0.71 V vs Fc^+/0^, consistent with formation of (TBA)[U(V){W_4_O_13_(OMe)_4_MoNO}_2_] (**7-U(V)(W**_**4**_**Mo)**_**2**_). Further analysis by ^1^H NMR spectroscopy (Figure S9) shows the presence of four resonances associated with the TBA cation and an additional signal at 4.26 ppm assigned to the –OMe groups of the [W_4_O_13_(OMe)_4_MoNO]^3–^ ligands. The shift of the signal corresponding to the bridging alkoxide moieties from that of **5-U(W**_**4**_**Mo)**_**2**_ (10.04 ppm) mirrors that reported previously for the all-molybdenum analogue.^33^ It was also possible to isolate **7-U(V)(W**_**4**_**Mo)**_**2**_ more directly using a “one-pot” method by treating **1-NaW**_**4**_**Mo** with UCl_4_ and excess [NO][PF_6_] in acetonitrile (see Experimental Section for details).^32^ Performing either synthesis with ^17^O enriched **1′-NaW**_**4**_**Mo** allows access to isotopically enriched **7-U(V)(W**_**4**_**Mo)**_**2**_; as such, characterization of **7-U(V)(W**_**4**_**Mo)**_**2**_ by ^17^O NMR spectroscopy was performed, with the results discussed in the Supporting Information (Section S2).

**Figure 7:**
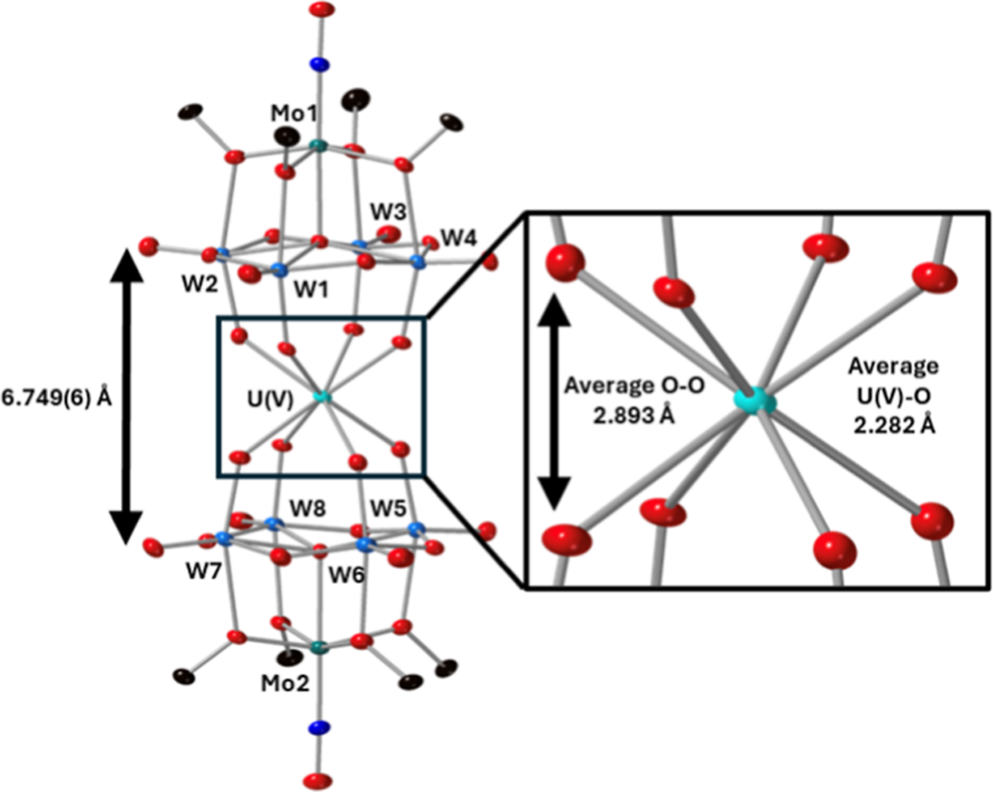
SCXRD structure of **7-U(V)(W**_**4**_**Mo)**_**2**_ with probability ellipsoids set at 50%. The tetrabutylammonium cation, solvent molecules and some disorder has been masked for clarity.

To verify the impact of increasing the oxidation state of the uranium center on the local coordination geometry, single crystals of **7-U(V)(W**_**4**_**Mo)**_**2**_ were grown by vapor diffusion of diethyl ether into a saturated solution of the crude material dissolved in dichloromethane at −30 °C. The brown single crystals were analyzed by SCXRD, resulting in the structure shown in Figure 7. The local eight coordinate square-antiprismatic geometry at the actinide center is retained, however the average U–O bond length is shorter, decreasing from ca. 2.36 Å in **5-U(W**_**4**_**Mo)**_**2**_ to ca. 2.28 Å in **7-U(V)(W**_**4**_**Mo)**_**2**_. This is consistent with the expected decrease in ionic radius, and increase in charge density, driving stronger electrostatic interactions between the U(V) center and the anionic {W_4_Mo} ligands. This mirrors exactly the behavior observed in the all-molybdenum analogues of these compounds, further exemplifying the lack of impact framework metal substitution has on the actinide–oxygen bond distances.^33^ A consequence of the decreased U–O bond length in **7-U(V)(W**_**4**_**Mo)**_**2**_ is a drop in the distance between the two-halves of the sandwich complex (approximated by the μ_5_-O-μ_5_-O distance) from 6.870(6) Å in **5-U(W**_**4**_**Mo)**_**2**_ to 6.749(6) Å in **7-U(V)(W**_**4**_**Mo)**_**2**_.^33^

**Figure 8:**
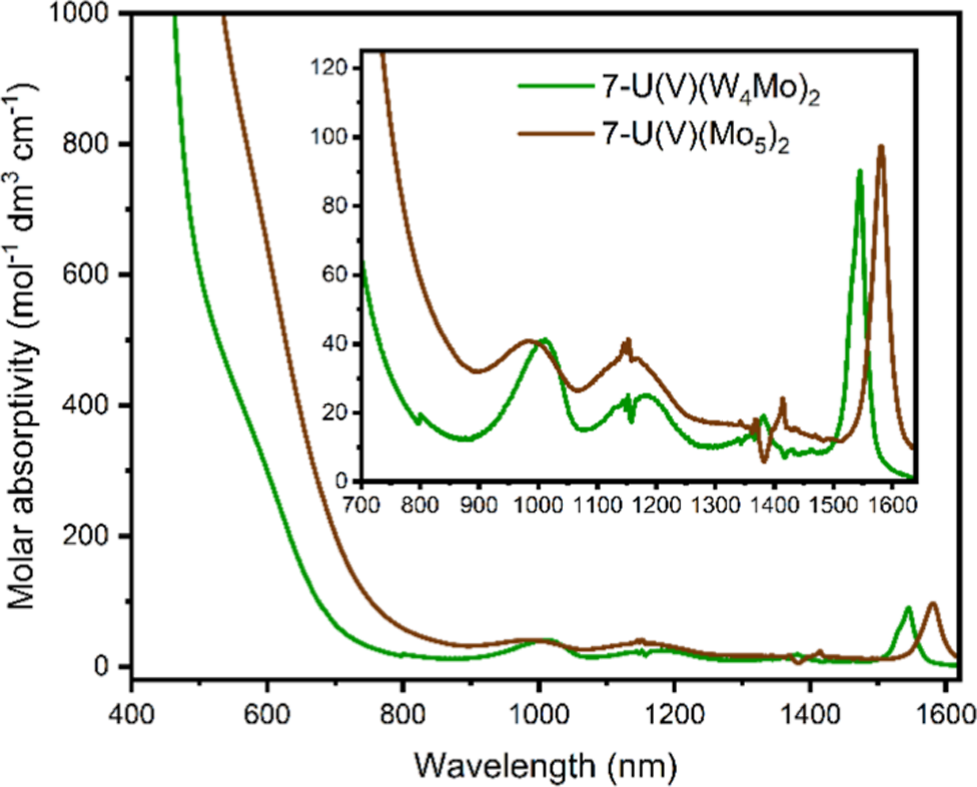
UV–vis–NIR spectra of **7-U(V)(W**_**4**_**Mo)**_**2**_ (green) and **7-U(V)(Mo**_**5**_**)**_**2**_ (brown). The spectra were collected in DCM at 21 °C.

The color change from green to brown upon oxidation from U(IV) to U(V) was interesting in the context of the variable MLCT behavior of the actinide centered sandwich complexes discussed in this work. We therefore pursued characterization of **7-U(V)(W**_**4**_**Mo)**_**2**_ by UV–vis–NIR spectroscopy. The UV–vis–NIR spectrum obtained from a solution of **7-U(V)(W**_**4**_**Mo)**_**2**_ in dichloromethane is shown in Figure 8 along with the spectrum of the all-molybdenum analogue ((TBA)[U(V){Mo_5_O_13_(OMe)_4_NO}_2_], **7-U(V)(Mo**_**5**_**)**_**2**_) for comparison.^33^ Both compounds show characteristic f–f transitions at approximately 1000, 1170, and 1560 nm. These transitions support oxidation of the uranium to U(V).^20,83,84^ Examining the shorter wavelengths of the spectra shown in Figure 8 shows the presence of a broad absorption spanning most of the visible region (400–800 nm). This can be assigned to MLCT between the U(V) center and the polyoxoalkoxide ligands (i.e., U(5f) → Mo(4d)). Regardless of the framework metal present, the MLCT absorption is more intense for the U(V) centered derivatives (i.e., **7-U(V)(W**_**4**_**Mo)**_**2**_ and **7-U(V)(Mo**_**5**_**)**_**2**_) when compared to their reduced analogues (i.e., **5-U(W**_**4**_**Mo)**_**2**_ and **5-U(Mo**_**5**_**)**_**2**_), implying that the MLCT process is favored following oxidation of the uranium (Figure S27). This is likely caused by the decrease in the U–O bond length, which pulls the polyoxoalkoxide ligands closer to the uranium center increasing orbital overlap between the uranium 5f orbitals and the LUMO. This should serve to increase the efficiency of MLCT and hence explains the relative increase in the intensity of this absorption for **7-U(V)(W**_**4**_**Mo)**_**2**_ and **7-U(V)(Mo**_**5**_**)**_**2**_. The relative drop in the intensity of the broad MLCT absorption peak between **7-U(W**_**4**_**Mo)**_**2**_ and **7-U(Mo**_**5**_**)**_**2**_ is consistent with the behavior displayed by both the U(IV) and Np(IV) centered systems. Therefore, as above, this can be attributed to the localization of the LUMO/LUMO+1 of **7-U(V)(W**_**4**_**Mo)**_**2**_ at the tips of the sandwich complex which decreases the overlap between the uranium 5f orbitals and the LUMO/LUMO+1.

## Conclusions

In conclusion, we report the extension of our series of actinide-polyoxoalkoxide sandwich-type complexes to those with the general formula (TBA)_2_[An{W_4_O_13_(OMe)_4_MoNO}_2_] where An = Th(IV), U(IV), and Np(IV). This extension was made possible by the development of an efficient and scalable synthesis of (TBA)_3_[W_5_O_18_MoNO], which is the direct precursor of the lacunary polyoxoalkoxide (**1-NaW**_**4**_**Mo**) that is employed in the synthesis of sandwich-type complexes. Oxidation of **5-U(W**_**4**_**Mo)**_**2**_ with [NO][PF_6_] allowed successful isolation of the U(V) centered sandwich complex. The new complexes were fully characterized, with both electronic absorption spectroscopy and CV, showing that the electronic properties of the series differ significantly from their isostructural all-molybdenum analogues. Specifically, charge transfer between the actinide center and the polyoxoalkoxide ligands is much weaker for the tungsten-containing compounds, while the redox chemistry is much less sensitive to the nature of the M(IV) ion present at the center of the sandwich-type complex. DFT calculations reveal both of these effects can be attributed to the change in the nature of the LUMOs when moving from sandwich-type complexes bearing [Mo_5_O_13_(OMe)_4_NO]^3–^ groups to those with [W_4_O_13_(OMe)_4_MoNO]^3–^. Framework substitution (i.e., swapping Mo for W) acts as a method of orbital engineering, driving localization of the LUMO and LUMO+1 on to the remaining Mo centers present at the tips of the sandwich-type complexes. This maximizes the spatial separation between the LUMO and LUMO+1, and between both orbitals and the central M(IV) ion, manifesting in the observed changes in both the UV–vis–NIR spectra and CVs. When combined with our previous work, these results show how both the framework metal and heterometal variation can be used to fine-tune the electronic properties of metal oxide materials.

## Supporting Information Available

Additional spectroscopic, crystallographic, voltametric data, and computational details (PDF)
XYZ coordinates of optimized structures discussed in this work (XYZ)

## Supplementary Material

ic5c00780_si_001.pdf

ic5c00780_si_002.xyz
