# Supplementary material for: Probing the Framework Metal Dependent Properties of Actinide-Centered Polyoxoalkoxide Sandwich-Type Complexes
Source: Inorg Chem. 2025 Apr 30;64(18):9180–94. doi: 10.1021/acs.inorgchem.5c00780 (PMC12076555; doi:10.1021/acs.inorgchem.5c00780)
Supplement: Supplementary file 1 — ic5c00780_si_001.pdf [file ic5c00780_si_001.pdf]

Electronic Supporting Information

Probing the framework metal dependent properties of actinide centered polyoxoalkoxide sandwich-type complexes

Dominic Shiels[1], Adriana C. Berlfein[1], Barbara M. T. C. Peluzo[1], Lauren M. Lopez[2†], Andrew W. Mitchell[2†], William W. Brennessel[1], Matthias Zeller[2], Matthew R. Crawley[3], Suzanne C. Bart[2*], Michael T. Ruggiero[1*], and Ellen M. Matson[1*]

[1] Department of Chemistry, University of Rochester, Rochester NY 14627 USA

[2] H. C. Brown Laboratory, James Tarpo Jr. and Margaret Tarpo, Department of Chemistry, Purdue University, West Lafayette, IN 47907, USA

[3] Department of Chemistry, University at Buffalo, The State University of New York, Buffalo NY 14620, USA.

[†]These authors contributed equally

Corresponding Author Contact Information:

Suzanne C. Bart: sbart@purdue.edu

Michael T. Ruggiero: michael.ruggiero@rochester.edu

Ellen M. Matson: matson@chem.rochester.edu

S1. ^{1}H NMR spectra S3

S2. ^{17}O NMR spectra S8

S3. UV-Vis-NIR spectra S16

S4. Cyclic voltammetry S21

S6 Single Crystal X-ray Diffraction S25

S7. Computational calculations S28

S8. References S66

S1. ^{1}H NMR spectra

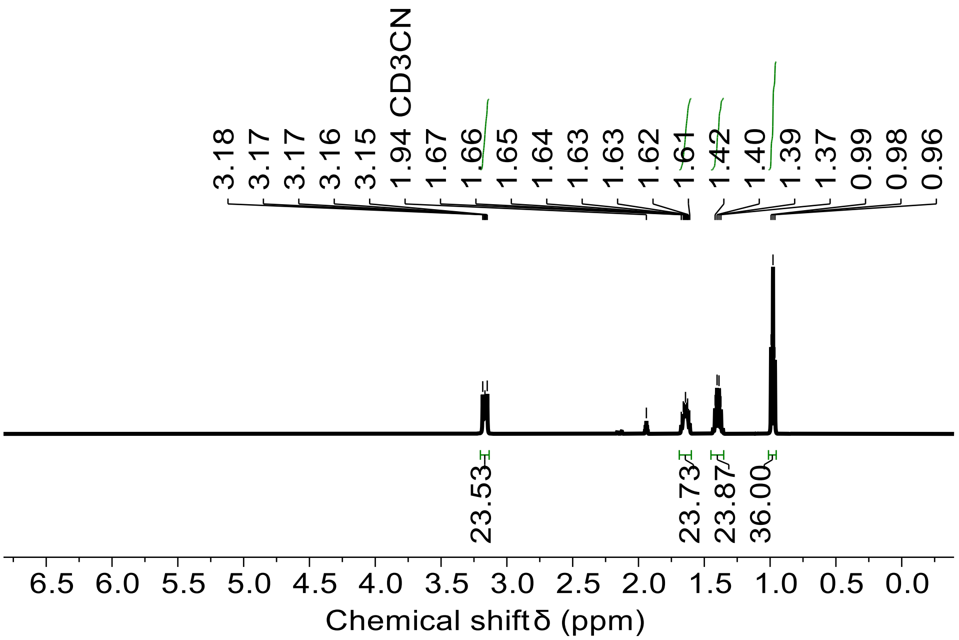

Figure S1. ^{1}H NMR spectrum (500 MHz) of $(TBA)_3[W_5O_{18}MoNO]$ in CD_3CN.

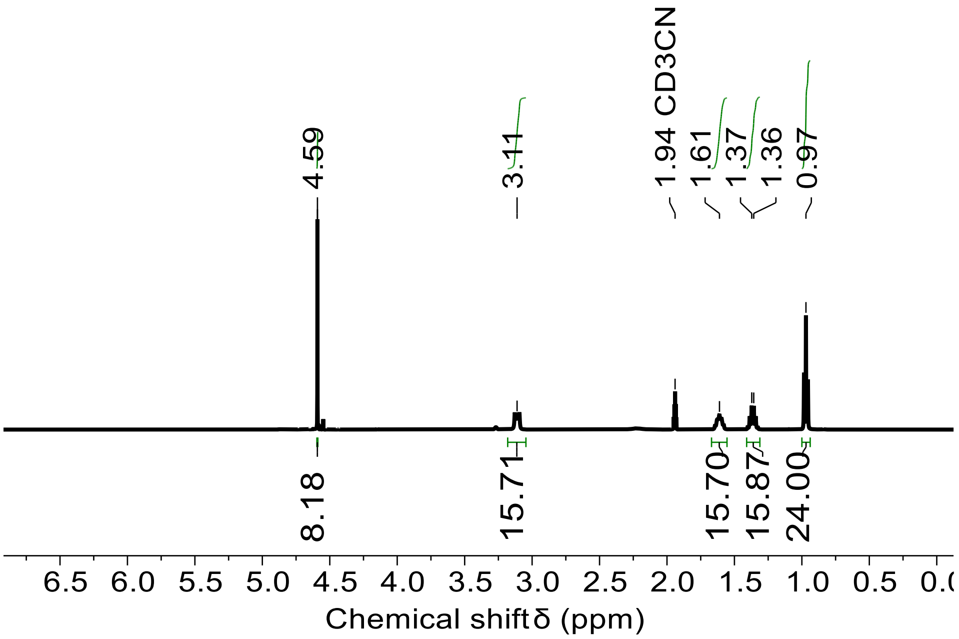

Figure S2. ^{1}H NMR spectrum (500 MHz) of $(TBA)_2[W_4O_{13}(OMe)_4MoNO][Na(MeOH)]$ (**1-NaW$_4$Mo**) in CD_3CN.

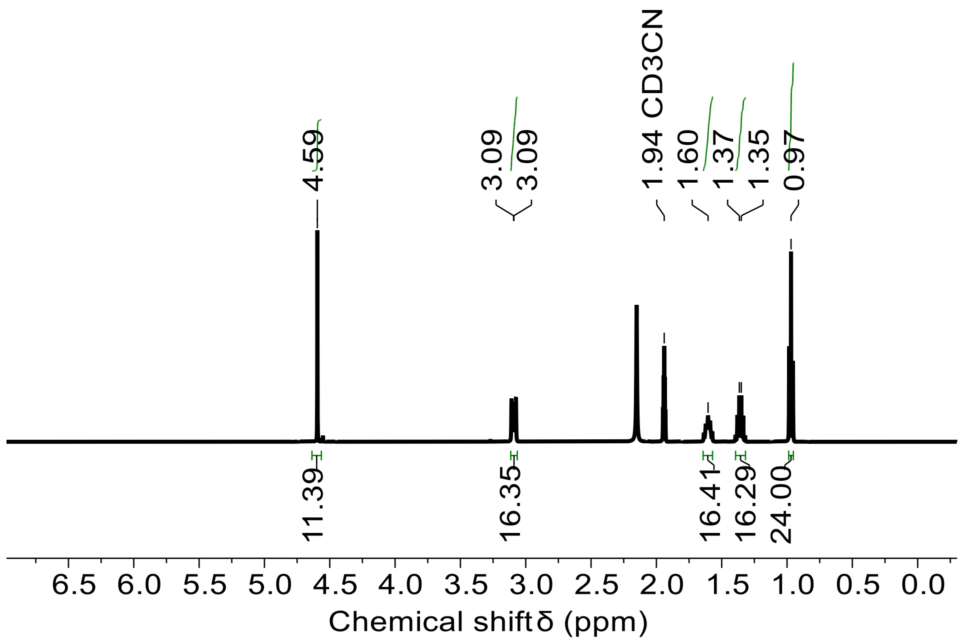

Figure S3. ^{1}H NMR spectrum (500 MHz) of ^{17}O enriched $(TBA)_2[W_4O_{13}(OMe)_4MoNO][Na(MeOH)]$ (**1'-NaW$_4$Mo**) in CD_3CN. The peak at ca. 2.2 ppm is attributed to water.

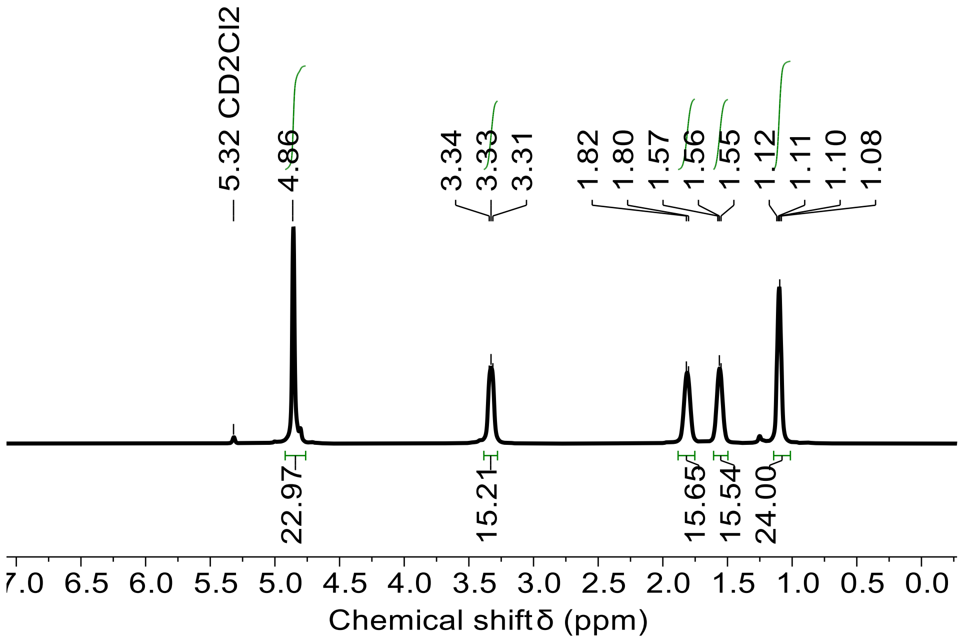

Figure S4. ^{1}H NMR spectrum (500 MHz) of $(TBA)_2[Zr\{W_4O_{13}(OMe)_4MoNO\}_2]$ (**2-Zr(W$_4$Mo)$_2$**) in CD_2Cl_2.

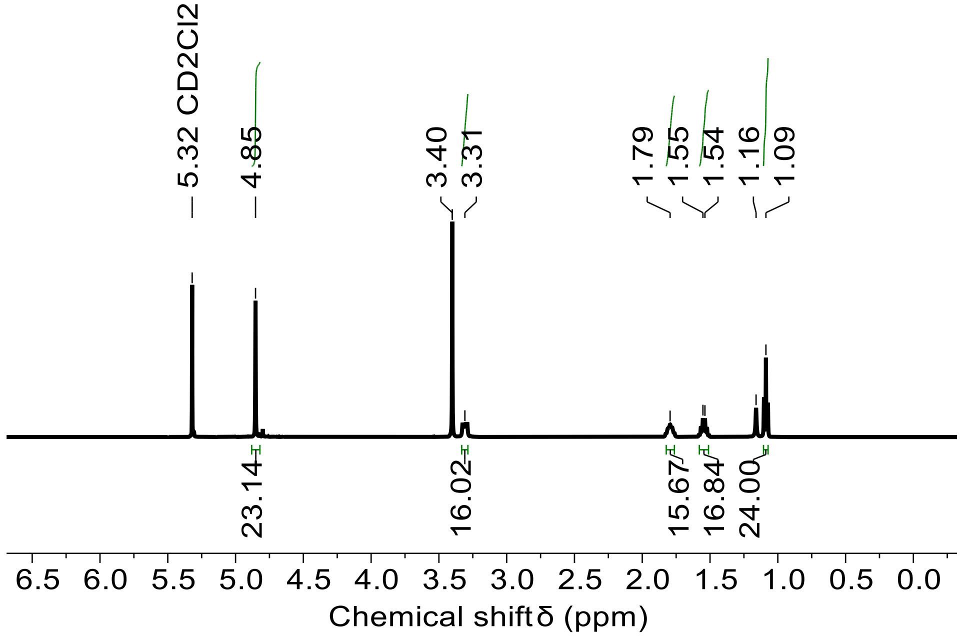

Figure S5. ^{1}H NMR spectrum (500 MHz) of $(TBA)_2[Hf\{W_4O_{13}(OMe)_4MoNO\}_2]$ (**3-Hf(W$_4$Mo)$_2$**) in CD_2Cl_2. Impurity peaks at 1.16 ppm and 3.40 ppm are attributed to MeOH.

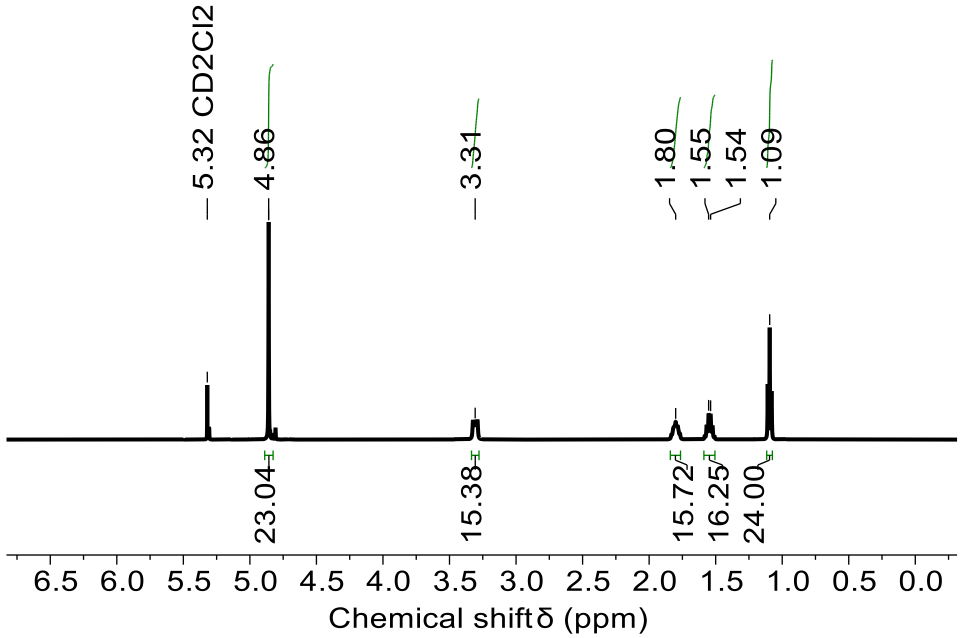

Figure S6. ^{1}H NMR spectrum (500 MHz) of $(TBA)_2[Th\{W_4O_{13}(OMe)_4MoNO\}_2]$ (**4-Th(W$_4$Mo)$_2$**) in CD_2Cl_2.

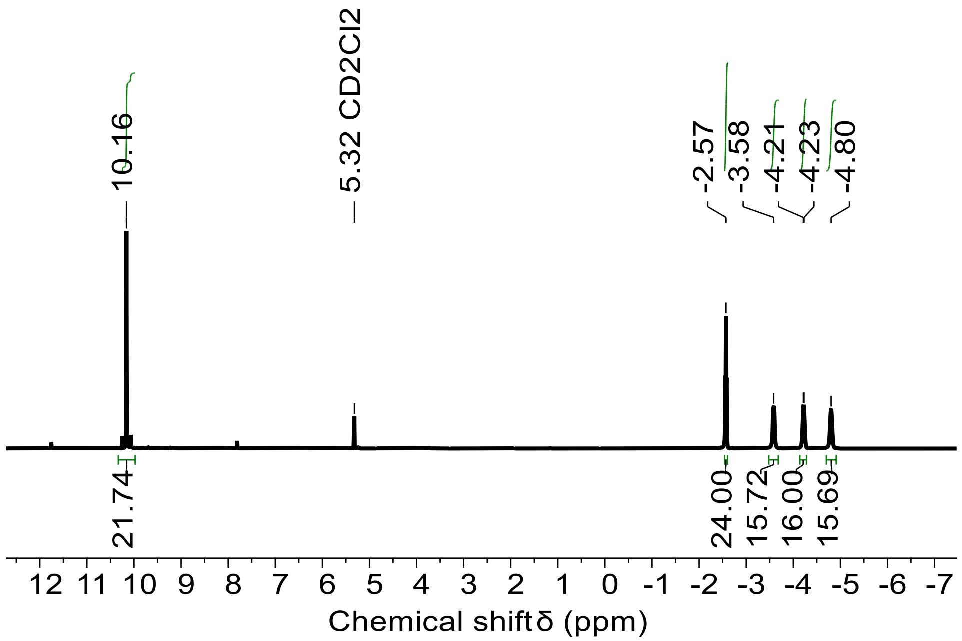

Figure S7. ^{1}H NMR spectrum (500 MHz) of $(TBA)_2[U\{W_4O_{13}(OMe)_4MoNO\}_2]$ (**5-U(W$_4$Mo)$_2$**) in CD_2Cl_2. Minor peaks at 7,81 ppm, 10.07 ppm, 10.24 ppm, and 11.76 ppm are consistently observed and are not assignable to common impurities. They may be caused by isotopologues of **5-U(W$_4$Mo)$_2$** (^{183}W has a natural abundance of 14.3% and spin ½) which have lower symmetry and therefore multiple inequivalent -OMe environments. These peaks are not observed in other ^{1}H NMR spectra of the other $M(W_4Mo)_2$ complexes studied, likely because the chemical environments of the various -OMe groups are extremely similar (and/or small values for $^{3}J_{HW}$ coupling constants), and therefore these peaks occur at essentially the same place as the major peak. However, the paramagnetic influence of U(IV) (f^2) could theoretically lead to larger differences in the chemical environments of the respective -OMe groups (or larger $^{3}J_{HW}$ coupling constants) and therefore allows observation of satellite peaks. However, additional experiments investigating this effect were deemed beyond the scope of this work and therefore this assignment is tentative at most.

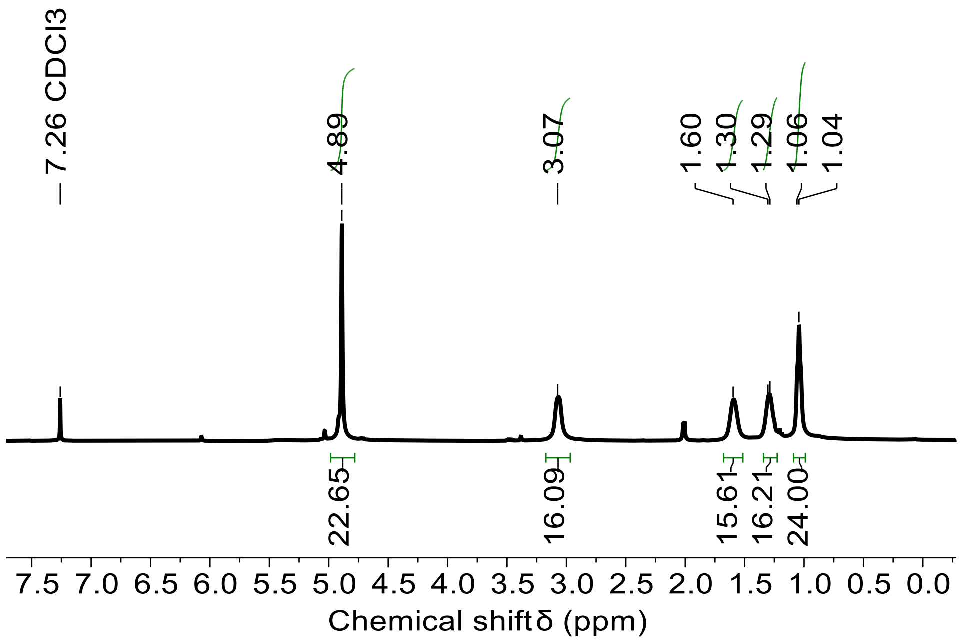

Figure S8. ^{1}H NMR spectrum (400 MHz) of $(TBA)_2[Np\{W_4O_{13}(OMe)_4MoNO\}_2]$ (**6-Np(W$_4$Mo)$_2$**) in $CDCl_3$. The peak at 3.38 ppm is attributed to dimethoxyethane, while peaks at 2.02 ppm, 5.03 ppm and 6.07 ppm are unknown impurities.

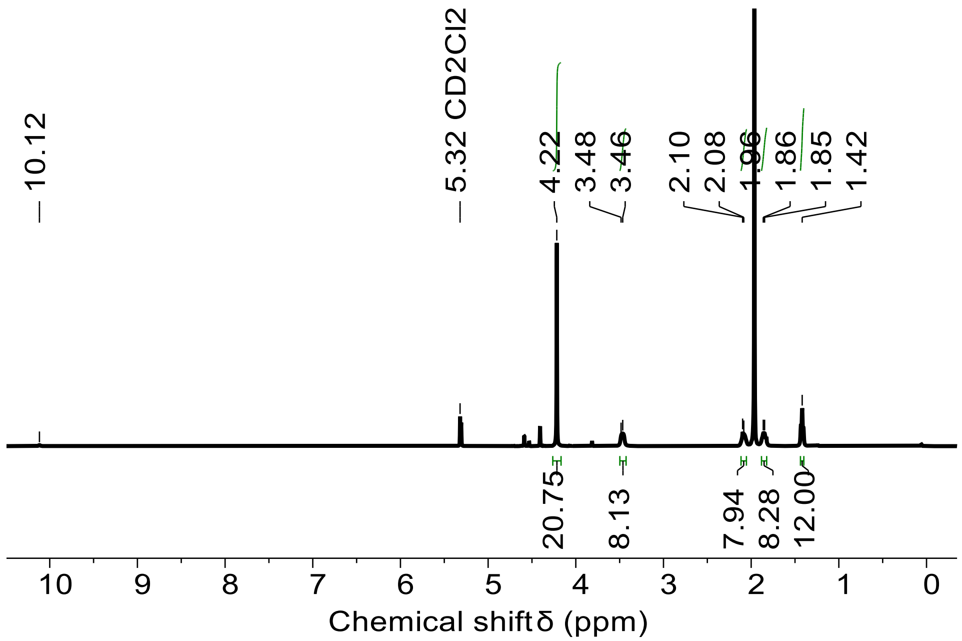

Figure S9. ^{1}H NMR spectrum (500 MHz) of $(TBA)[U\{W_4O_{13}(OMe)_4MoNO\}_2]$ (**7-U(V)(W$_4$Mo)$_2$**) in CD_2Cl_2. Large impurity peak at 1.96 ppm is attributed to MeCN. Minor impurity peaks at 3.82 ppm and 4.4-4.6 ppm are attributed to unknown decomposition products.

S2.^{17}O NMR spectra

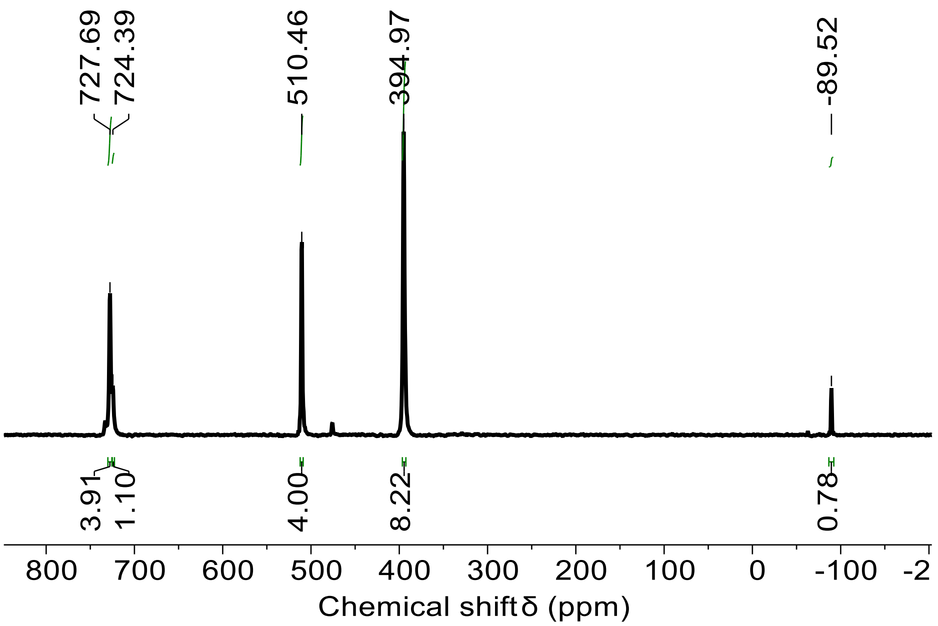

Figure S10. ^{17}O NMR spectrum (67.8 MHz) of ^{17}O enriched $(TBA)_3[W_5O_{18}MoNO]$ in CD_3CN.

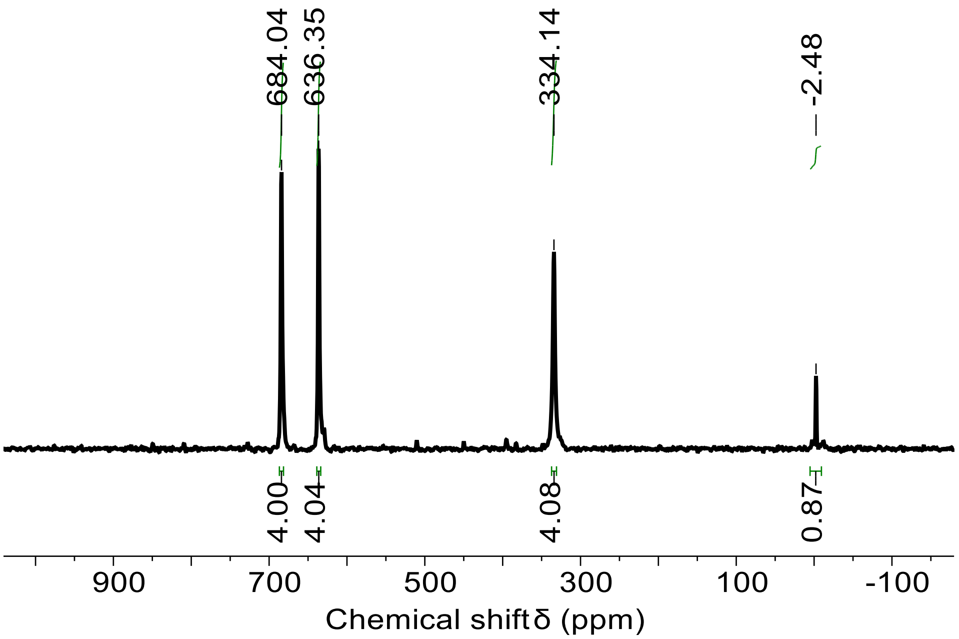

Figure S11. ^{17}O NMR spectrum (67.8 MHz) of ^{17}O enriched $(TBA)_2[W_4O_{13}(OMe)_4MoNO][Na(MeOH)]$ (**1-NaW$_4$Mo**) in CD_3CN.

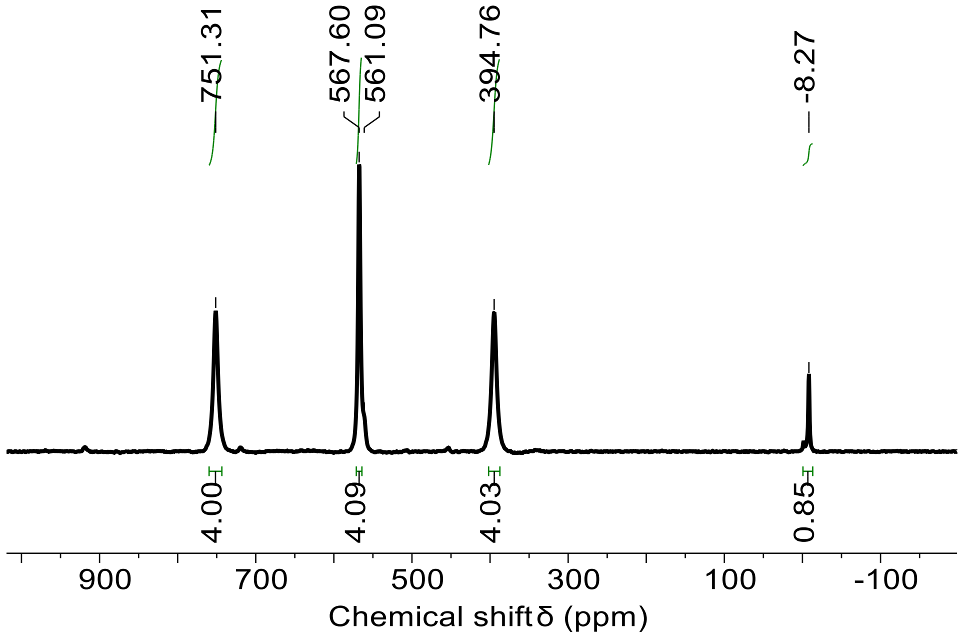

Figure S12. ^{17}O NMR spectrum (67.8 MHz) of ^{17}O enriched $(TBA)_2[Zr\{W_4O_{13}(OMe)_4MoNO\}_2]$ (**2-Zr(W$_4$Mo)$_2$**) in CD_2Cl_2.

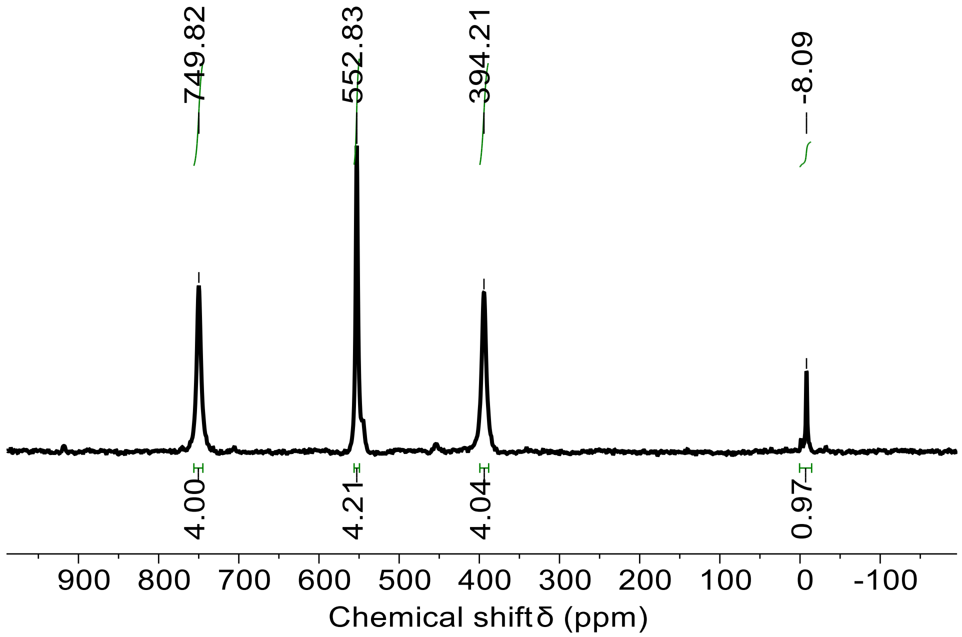

Figure S13. ^{17}O NMR spectrum (67.8 MHz) of ^{17}O enriched $(TBA)_2[Hf\{W_4O_{13}(OMe)_4MoNO\}_2]$ (**3-Hf(W$_4$Mo)$_2$**) in CD_2Cl_2.

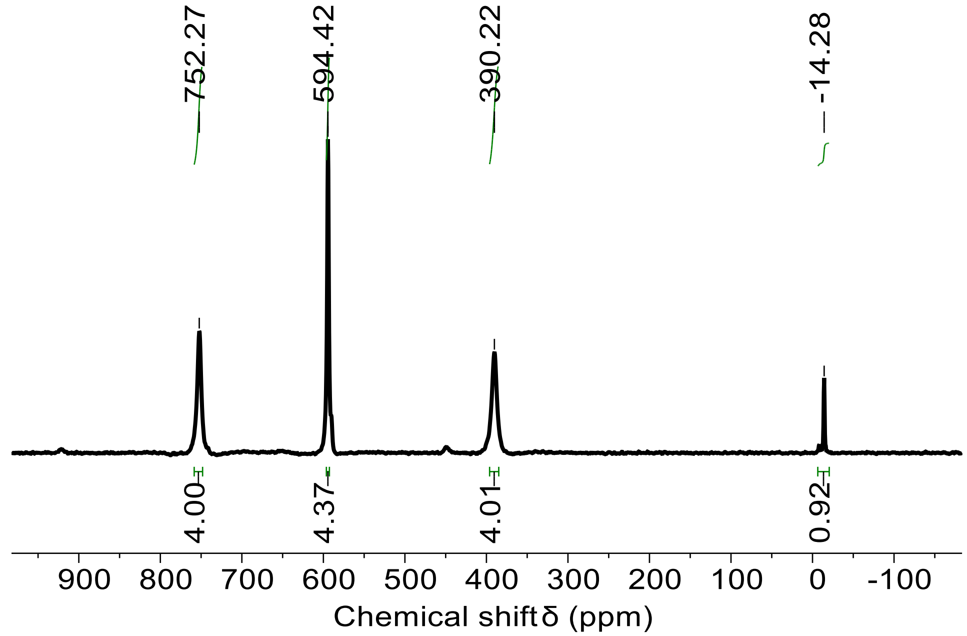

Figure S14. ^{17}O NMR spectrum (67.8 MHz) of ^{17}O enriched $(TBA)_2[Th\{W_4O_{13}(OMe)_4MoNO\}_2]$ (**4-Th(W$_4$Mo)$_2$**) in CD_2Cl_2.

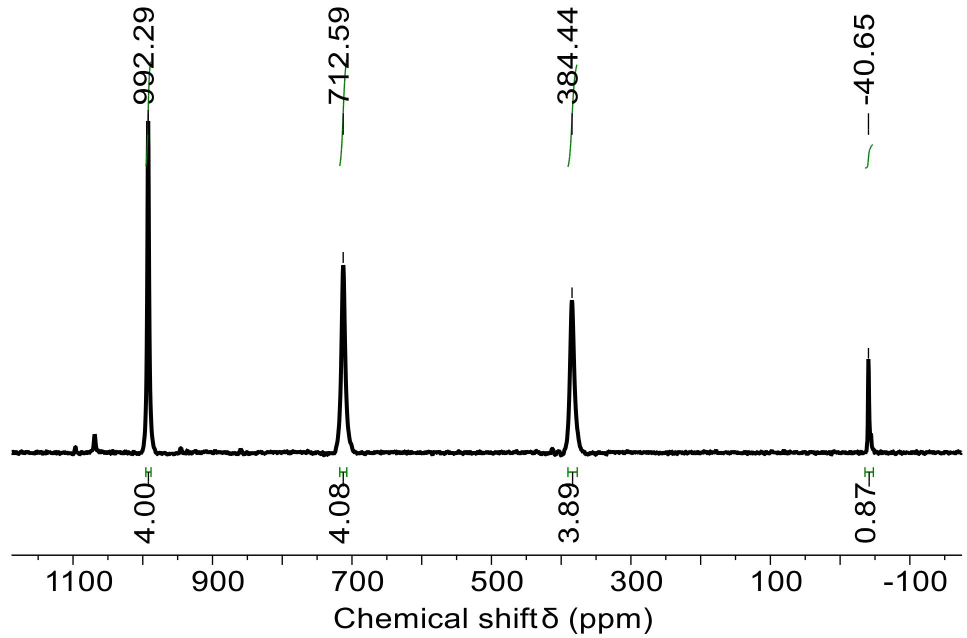

Figure S15. ^{17}O NMR spectrum (67.8 MHz) of ^{17}O enriched $(TBA)_2[U\{W_4O_{13}(OMe)_4MoNO\}_2]$ (**5-U(W$_4$Mo)$_2$**) in CD_2Cl_2.

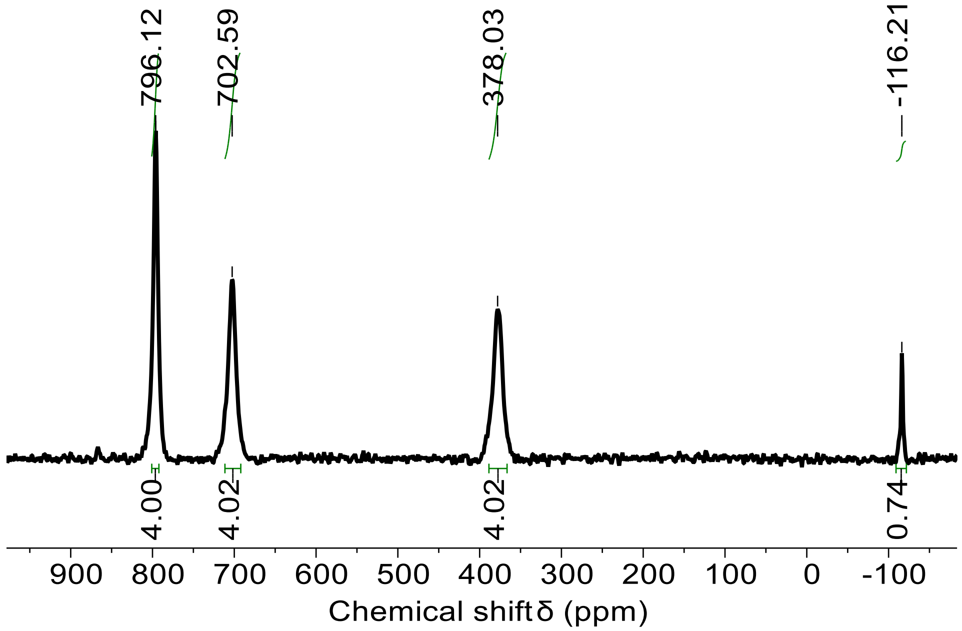

Figure S16. ^{17}O NMR spectrum (54.2 MHz) of ^{17}O enriched $(TBA)_2[Np\{W_4O_{13}(OMe)_4MoNO\}_2]$ (**6-Np(W$_4$Mo)$_2$**) in $CDCl_3$.

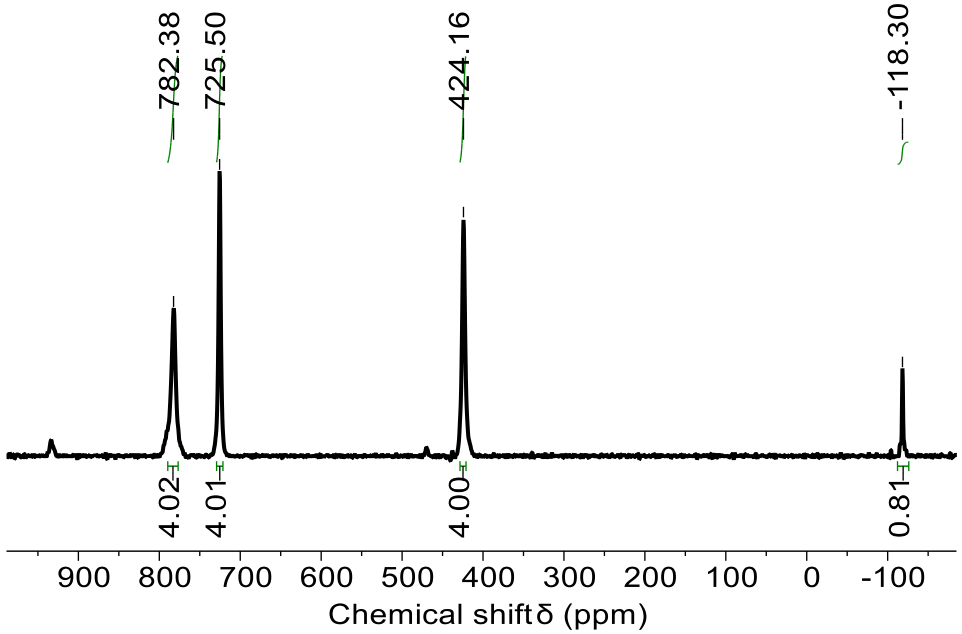

Figure S17. ^{17}O NMR spectrum (67.8 MHz) of ^{17}O enriched $(TBA)[U\{W_4O_{13}(OMe)_4MoNO\}_2]$ (**7-U(V)(W$_4$Mo)$_2$**) in CD_2Cl_2.

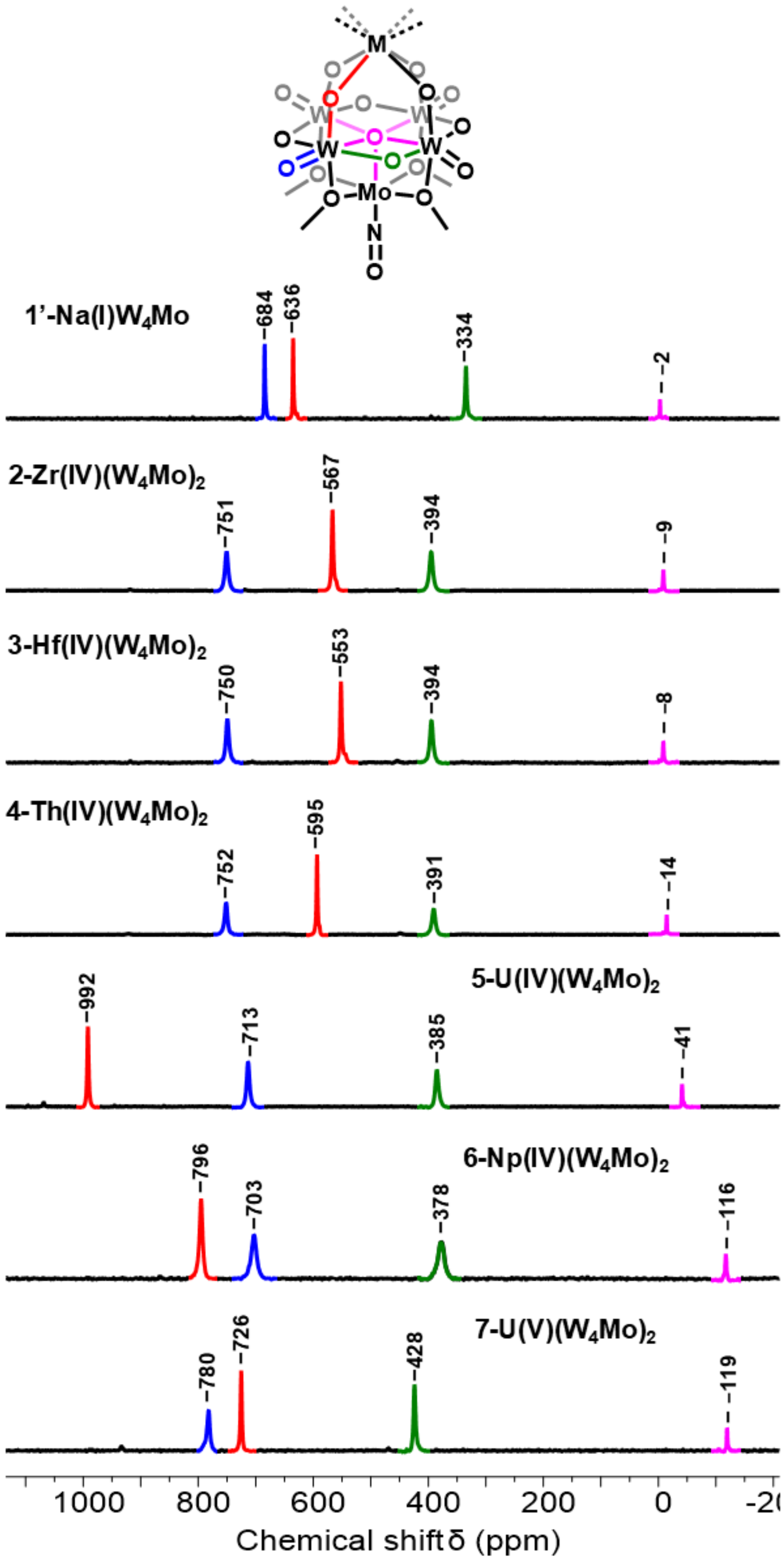

Figure S18. Stack of the ^{17}O NMR spectra **1'-NaW$_4$Mo**, **2-Zr(W$_4$Mo)$_2$**, **3-Hf(W$_4$Mo)$_2$**, **4-Th(W$_4$Mo)$_2$**, **5-U(W$_4$Mo)$_2$**, **6-Np(W$_4$Mo)$_2$**, and **7-U(V)(W$_4$Mo)$_2$**,

2.1 Discussion and assignment of ^{17}O NMR spectra

Recently, we showed that treatment of **1-NaMo$_5$** with ^{17}O labelled water allows efficient ^{17}O enrichment of the metal-oxo groups of the polyoxoalkoxide. This enriched material can then be treated with metal(IV) chlorides to yield ^{17}O labelled sandwich-type complexes.[1] This allowed characterization of the series by ^{17}O NMR spectroscopy, which is an insightful technique that provides detailed information about structure, bonding, and dynamic processes present in solution.[2, 3] The chemical shifts of the peaks in the ^{17}O NMR spectrum can be correlated with the degree of M–O π-bonding (M being the framework metal), with higher π-bond order (or decreased M–O bond length) resulting in more positive chemical shifts.[2, 3] In our previous work, focused complexes with the general formula $(TBA)_2[M\{Mo_5O_{13}(OMe)_4NO\}_2]$ (M = Zr, Hf, Th, U, and Np), ^{17}O NMR spectroscopy revealed subtle differences in the extent of Mo-O π-bonding in the Mo-O-M bridges depending on the heterometal present, with the transition metal centered complexes (i.e. M = Zr and Hf) competing for oxygen π-electron density (and therefore limiting Mo←O backbonding) and therefore lowering the Mo-O bond order.

It was hypothesized that similar levels of ^{17}O enrichment of **1-NaW$_4$Mo** (and the sandwich-type complexes formed from this material) should be obtainable by treatment of **1-NaW$_4$Mo** with five equivalents of 40% ^{17}O enriched water at 50 °C in methanol.[1] The ^{17}O NMR spectrum of the material obtained after drying and recrystallization is shown at the top of **Figure S11**. The spectrum, recorded in CD_3CN, shows successful ^{17}O enrichment, giving **1'-NaW$_4$Mo**, and features four resonances corresponding to the four different types of metal-oxo groups present in the structure of **1'-NaW$_4$Mo**. The central μ_5-oxo (magenta) and bridging W-O-W groups (green) are in regions typical for central and bridging metal-oxo groups respectively, and therefore their assignment was straightforward. The two remaining peaks, found at 636 and 684 ppm respectively, have chemical shifts closer to what is typical for terminal W=O groups (though these are often reported at >700 ppm).[2-4] Exact assignment of these peaks is more difficult, however the peak at 636 ppm is tentatively assigned to the bridging W-O-Na groups as it is assumed that interactions between the oxygen nuclei and sodium cation will slightly weaken the W-O π-bonding interaction, leading to a longer W-O bond length and a lower chemical shift. The remaining peak, observed at 684 ppm, is assigned to the terminal W=O groups.

Employing ^{17}O enriched **1'-NaW$_4$Mo** in the synthesis of the sandwich-type complexes described in the main text allows for successful isotopic labelling of the clusters, with the obtained ^{17}O NMR spectra of **2-Zr(W$_4$Mo)$_2$**, **3-Hf(W$_4$Mo)$_2$**, **4-Th(W$_4$Mo)$_2$**, **5-U(W$_4$Mo)$_2$**, **6-Np(W$_4$Mo)$_2$**, and **7-U(W$_4$Mo)$_2$** shown in **Figures S12-S18**. The spectra of the diamagnetic sandwich-type complexes **2-Zr(W$_4$Mo)$_2$**, **3-Hf(W$_4$Mo)$_2$**, and **4-Th(W$_4$Mo)$_2$** are similar in many regards, with the peaks assigned to the central μ_5-O, the bridging W-O-W groups, and the W=O groups all observed at very similar positions (± 6 ppm). Relative to **1'-NaW$_4$Mo**, the peaks assigned to the W-O-W groups and the W=O groups in **2-Zr(W$_4$Mo)$_2$**, **3-Hf(W$_4$Mo)$_2$**, and **4-Th(W$_4$Mo)$_2$** appear around 60-70 ppm more downfield. This can be attributed to lower effective negative charge per $\{W_4MO\}$ unit, moving from 2− in **1'-NaW$_4$Mo** to 1− in the sandwich-type complexes, which serves to effectively "deshield" the oxygen nuclei in the sandwich-type complexes when compared **1'-NaW$_4$Mo**.

These same peaks are observed 200-250 ppm further downfield in the corresponding all molybdenum compounds (i.e. $(TBA)_2[M\{Mo_5O_{13}(OMe)_4NO\}_2]$), which is a consistent feature when comparing the ^{17}O NMR spectra of isostructural polyoxometalates which only differ by framework metal (Mo vs. W). It is caused by the presence of longer, more ionic, M-O bonds in polyoxotungstates compared to polyoxomolybdates.[3] Much less variation is seen in the position of the peak assigned to the central μ_5-oxo center (appearing between 25 to −25 ppm in all the polyoxoalkoxides studied), showing that this chemical environment is less impacted by the changes in structure or framework metal.[1] This speaks to the fact that this nucleus can be approximated as an isolated O^{2-} trapped in the center of these polyoxoalkoxides.

As may be expected, there is a more significant variation in the position of the peak assigned to the M-O-W groups (highlighted in red in **Figure S18**) in the diamagnetic polyoxoalkoxides, appearing between 636 ppm in **1'-NaW$_4$Mo** to 553 ppm in **3-Hf(W$_4$Mo)$_2$**. This variation is caused by the change in the π-character of the W-O bond as a function of the heterometal.[2, 3] The second and third row transitions metals (Zr and Hf) have access to relatively low energy empty d-orbitals which are available for O(2p)→M(nd) (n = 4 or 5) π-back bonding. These heterometals can therefore compete with tungsten for oxygen π-electron density, leading to W-O bonds with lower π-character, and consequently lower chemical shifts. Conversely, Na and Th are less able to accept π-electron density from oxygen and therefore tend to form more ionic bonds. This removes the competition for π-electron density and therefore allows the formation of W-O bonds with more π-character, which appear at higher chemical shifts (closer to the typical region for W=O multiple bonds).

The ^{17}O NMR spectra of **5-U(W$_4$Mo)$_2$** and **6-Np(W$_4$Mo)$_2$** feature more drastic variations in the position of the peak associated with the oxygen nuclei of the An-O-W groups (highlighted in red). This can be attributed to the influence of the unpaired electrons of the paramagnetic An(IV) centers present on the chemical environment of the adjacent oxygen nuclei. Interestingly, for **5-U(W$_4$Mo)$_2$** (U(IV), f^2), this peak is around 150 ppm further downfield than the corresponding peak in the spectrum of **6-Np(W$_4$Mo)$_2$** (Np(IV), f^3), which occurs much closer to the where this peaks is observed in the diamagnetic systems discussed above. This appears to mirror the behavior observed in the ^1H NMR spectra of the compounds, where the U(IV) centered complex has a very different spectrum to any of the other compounds present, including the Np(IV) centered complex. This clearly displays that this behavior is specific to the actinide present, and the specific electronic configuration of that actinide, rather than simply being a function of incorporation of a paramagnetic metal center.

The majority of the remaining peaks in the ^{17}O NMR spectra of **5-U(W$_4$Mo)$_2$** and **6-Np(W$_4$Mo)$_2$** are much less affected by the paramagnetic metal center, with the peaks corresponding to the oxygen nuclei of the bridging W-O-W groups (highlighted in green) and terminal W=O groups (highlighted in blue) appearing much closer to the corresponding peaks in the ^{17}O NMR spectra of the complexes that are centered by diamagnetic heterometals. The peak assigned to the central μ_5-O nuclei (highlighted in magenta) is found more upfield in the ^{17}O NMR spectrum of **5-U(W$_4$Mo)$_2$**, appearing at −41 ppm, and even more so in the ^{17}O NMR spectrum of **6-Np(W$_4$Mo)$_2$**, appearing at −116 ppm. These are significantly different

to the corresponding peaks in the spectra of **1'-NaW$_4$Mo**, **2-Zr(W$_4$Mo)$_2$**, **3-Hf(W$_4$Mo)$_2$**, **4-Th(W$_4$Mo)$_2$**, which may be a consequence of the fact the central μ_5-O nuclei are spatially much closer to the paramagnetic actinides in **5-U(W$_4$Mo)$_2$** and **6-Np(W$_4$Mo)$_2$** than the bridging W-O-W groups and terminal W=O groups.

The spectrum of **7-U(V)(W$_4$Mo)$_2$**, obtained after oxidation of the U(IV) center of **5-U(W$_4$Mo)$_2$** to U(V), is shown at the bottom of **Figure S18**. The peaks assigned to the bridging W-O-W groups (green) and terminal W=O groups are observed further downfield in the ^{17}O NMR spectrum of **7-U(V)(W$_4$Mo)$_2$** than for any other complex studied. This is most likely a result of the decrease in the overall negative charge present in the system after oxidation of U(IV) to U(V) (now only 1− per two {Mo$_5$} units) which serves to "deshield" these nuclei. A similar effect was previously seen upon oxidation of the corresponding all-molybdenum derivative.[1] The remaining peaks, assigned to the bridging W-O-U groups and the central μ_5-oxo group are observed at 726 ppm and −119 ppm respectively. These peaks appear closer in position to the corresponding peaks in the ^{17}O NMR spectrum of **6-Np(W$_4$Mo)$_2$** (Np(IV), f^3) than in the spectrum of **5-U(W$_4$Mo)$_2$** (Np(IV), f^2). The reason for this is not entirely known, however the fact that the NMR behavior of the complexes containing an odd f-electrons (i.e. **6-Np(W$_4$Mo)$_2$** and **7-U(V)(W$_4$Mo)$_2$**) is very similar and contrasts the behavior of **5-U(W$_4$Mo)$_2$** (which possesses an even number of f-electrons), is intriguing. We are therefore actively looking to extend is series of actinide-containing sandwich-type complexes to attempt to see if this behavior persists.

S3. UV-Vis-NIR spectra

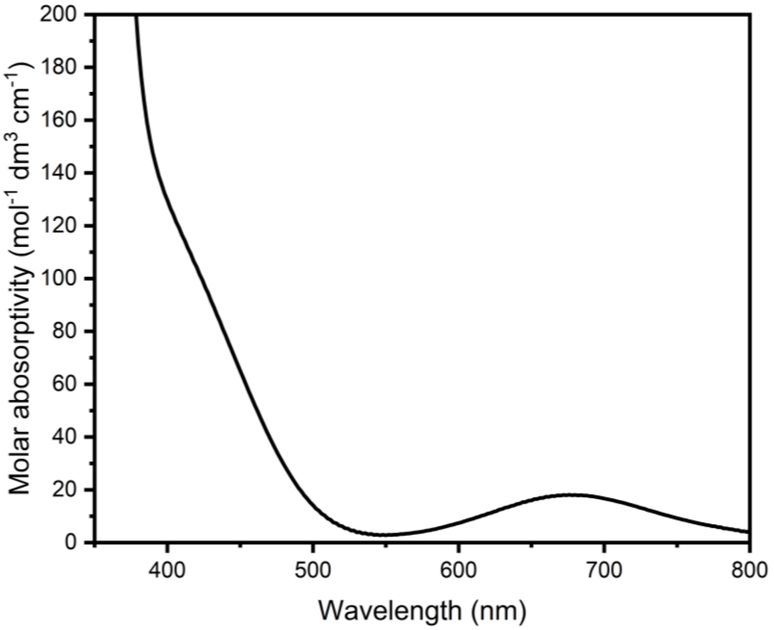

Figure S19. UV-Vis spectrum of 1 mM $(TBA)_3[W_5O_{18}MoNO]$ in MeCN. Spectrum recorded at 21 °C.

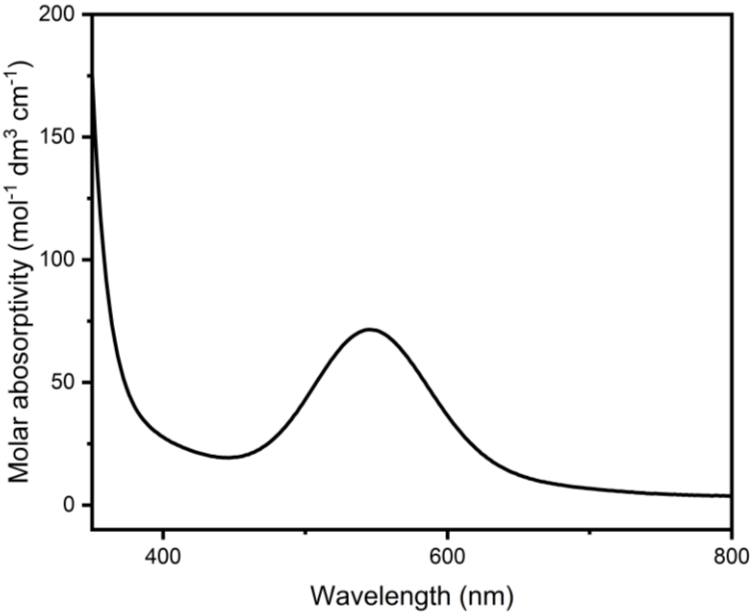

Figure S20. UV-Vis spectrum of 1 mM $(TBA)_2[W_4O_{13}(OMe)_4MoNO][Na(MeOH)]$ (**1-NaW$_4$Mo**) in MeCN. Spectrum recorded at 21 °C.

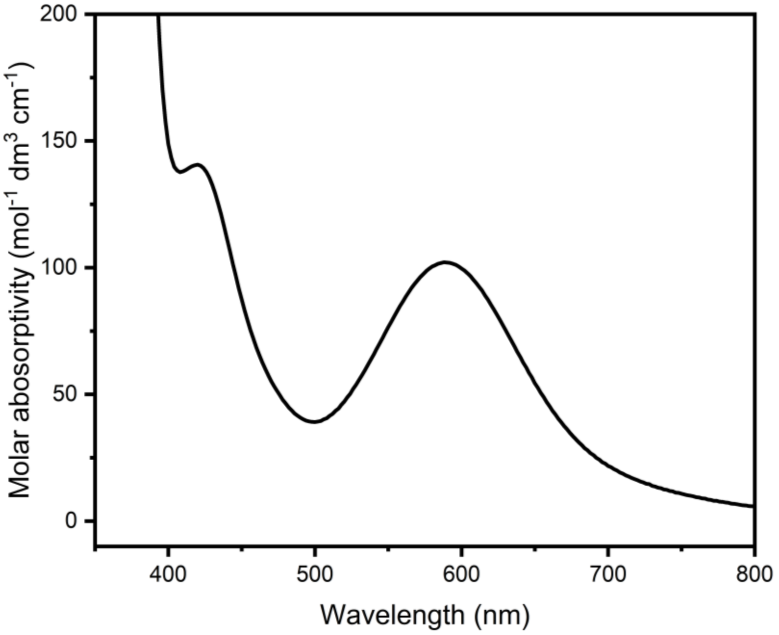

Figure S21. UV-Vis spectrum of 1 mM $(TBA)_2[Zr\{W_4O_{13}(OMe)_4MoNO\}_2]$ (**2-Zr(W$_4$Mo)$_2$**) in MeCN. Spectrum recorded at 21 °C.

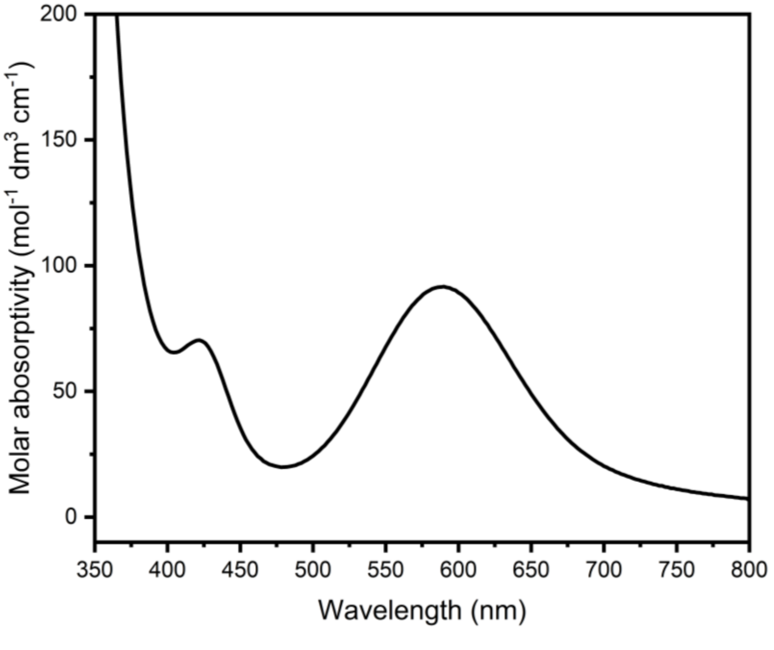

Figure S22. UV-Vis spectrum of 1 mM $(TBA)_2[Hf\{W_4O_{13}(OMe)_4MoNO\}_2]$ (**3-Hf(W$_4$Mo)$_2$**) in MeCN. Spectrum recorded at 21 °C.

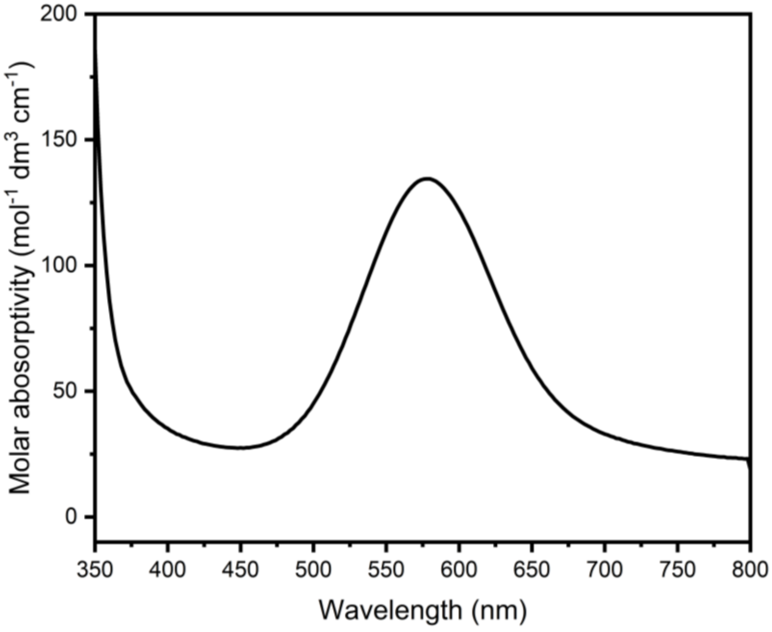

Figure S23. UV-Vis spectrum of 1 mM $(TBA)_2[Th\{W_4O_{13}(OMe)_4MoNO\}_2]$ (**4-Th(W$_4$Mo)$_2$**) in MeCN. Spectrum recorded at 21 °C.

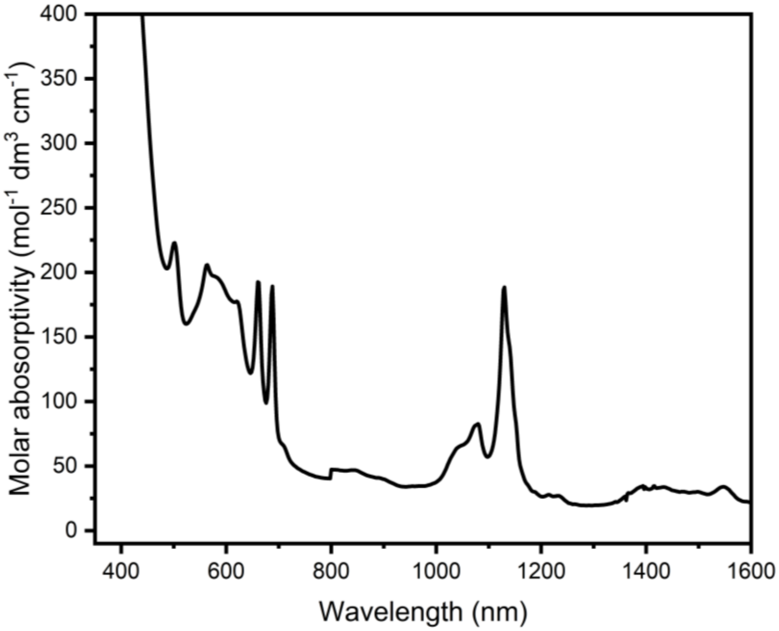

Figure S24. UV-Vis-NIR spectrum of 1 mM $(TBA)_2[U\{W_4O_{13}(OMe)_4MoNO\}_2]$ (**5-U(W$_4$Mo)$_2$**) in MeCN. Spectrum recorded at 21 °C.

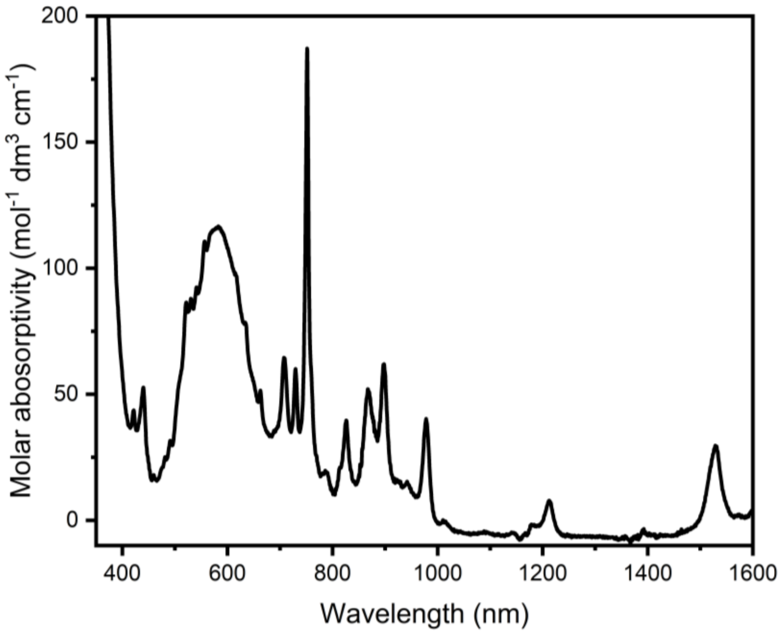

Figure S25. UV-Vis-NIR spectrum of 1 mM $(TBA)_2[Np\{W_4O_{13}(OMe)_4MoNO\}_2]$ (**6-Np(W$_4$Mo)$_2$**) in MeCN. Spectrum recorded at 21 °C.

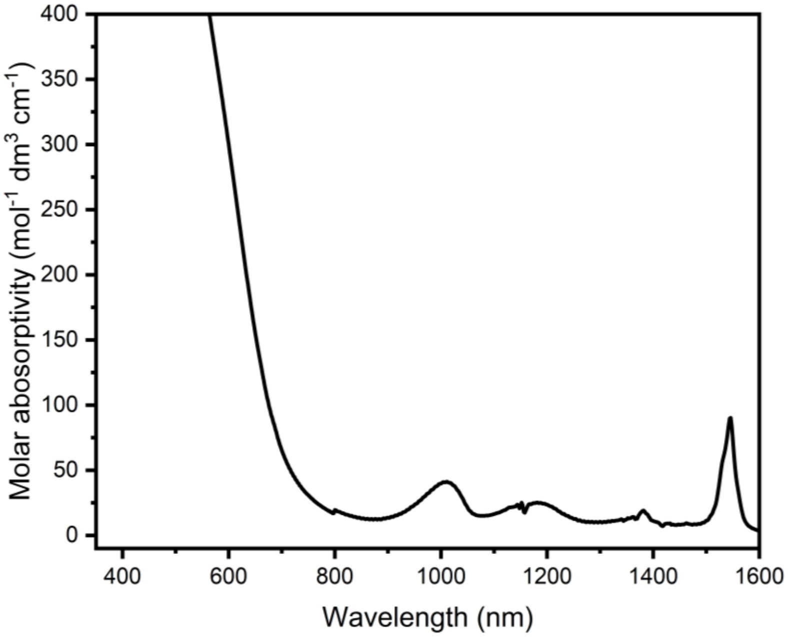

Figure S26. UV-Vis-NIR spectrum of 1 mM $(TBA)[U\{W_4O_{13}(OMe)_4MoNO\}_2]$ (**7-U(W$_4$Mo)$_2$**) in DCM. Spectrum recorded at 21 °C.

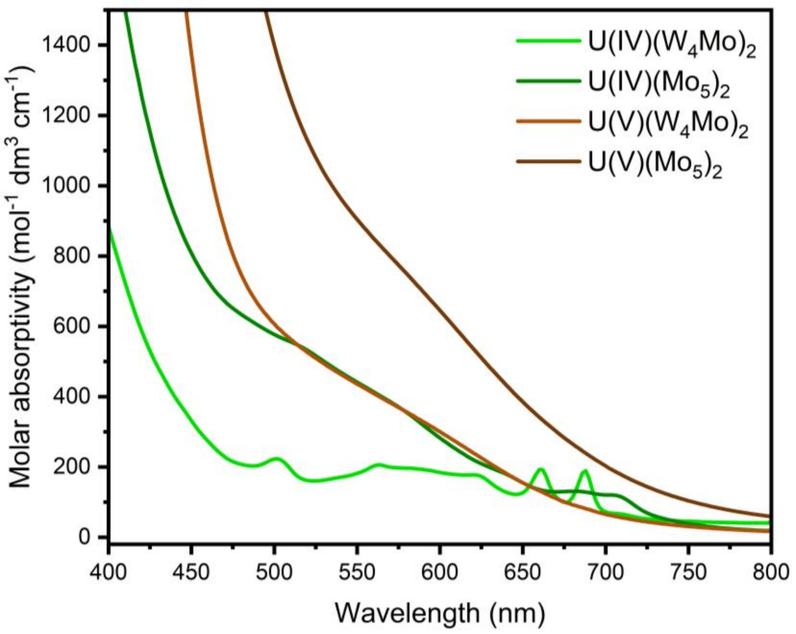

Figure S27. Stack of the UV-Vis-NIR spectra of **5-U(W$_4$Mo)$_2$**, **5-U(Mo$_5$)$_2$**, **7-U(W$_4$Mo)$_2$**, and **7-U(Mo$_5$)$_2$**.

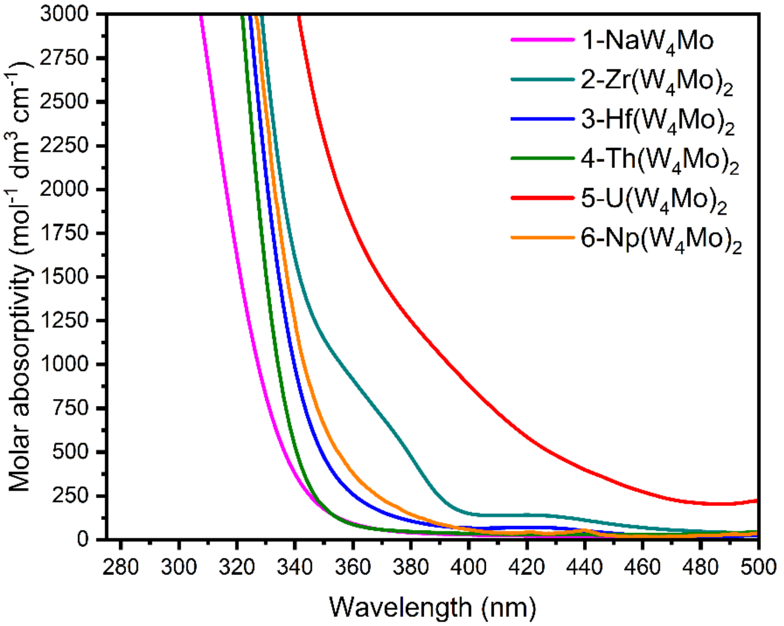

Figure S28. UV-Vis-NIR spectra of **1-NaW$_4$Mo**, **2-Zr(W$_4$Mo)$_2$**, **3-Hf(W$_4$Mo)$_2$**, **4-Th(W$_4$Mo)$_2$**, **5-U(W$_4$Mo)$_2$** and **6-Np(W$_4$Mo)$_2$** focusing on the 275-500 nm region.

S4. Cyclic voltammetry

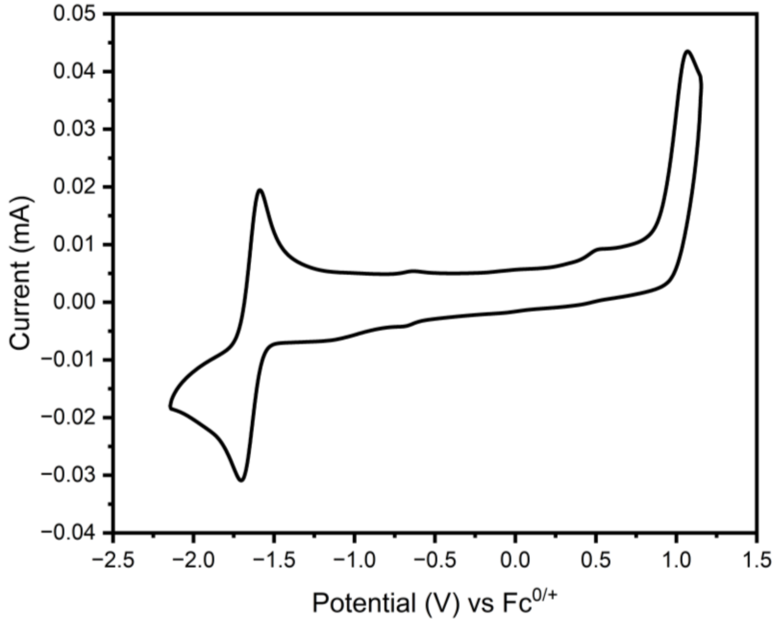

Figure S29. Cyclic voltammogram of **1-NaW$_4$Mo**. The data was acquired in MeCN with 0.1 M TBA(PF_6) supporting electrolyte, 1 mM of cluster, and a scan rate of 200 mV s^{-1}. The open circuit potential (OCP) was −1.10 V.

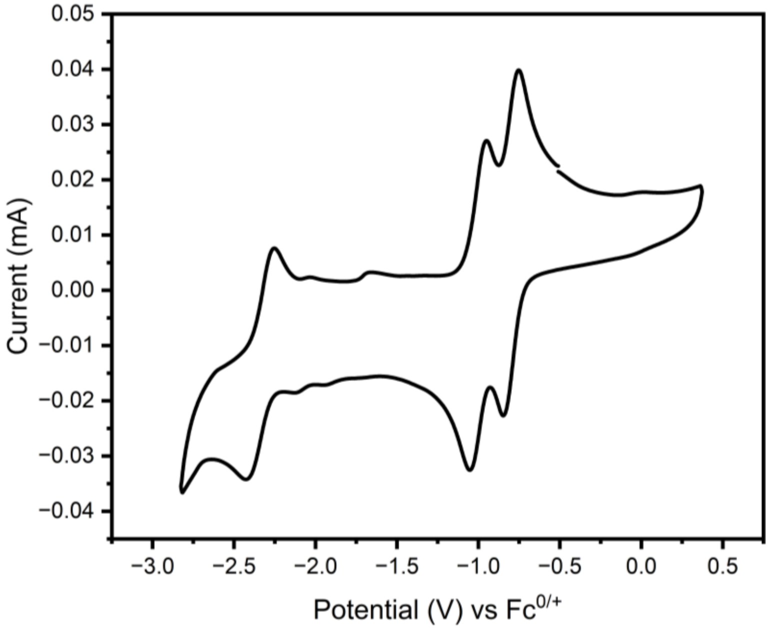

Figure S30. Cyclic voltammogram of **2-Zr(W$_4$Mo)$_2$**. The data was acquired in MeCN with 0.1 M TBA(PF_6) supporting electrolyte, 1 mM of cluster, and a scan rate of 200 mV s^{-1}. The open circuit potential (OCP) was −0.51 V.

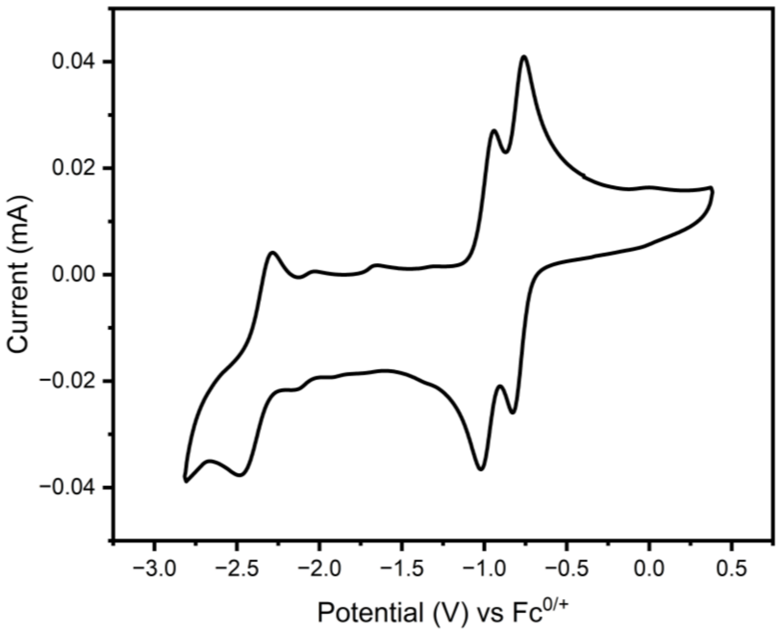

Figure S31. Cyclic voltammogram of **3-Hf(W_4Mo)$_2$**. The data was acquired in MeCN with 0.1 M TBA(PF_6) supporting electrolyte, 1 mM of cluster, and a scan rate of 200 mV s^{-1}. The open circuit potential (OCP) was −0.40 V.

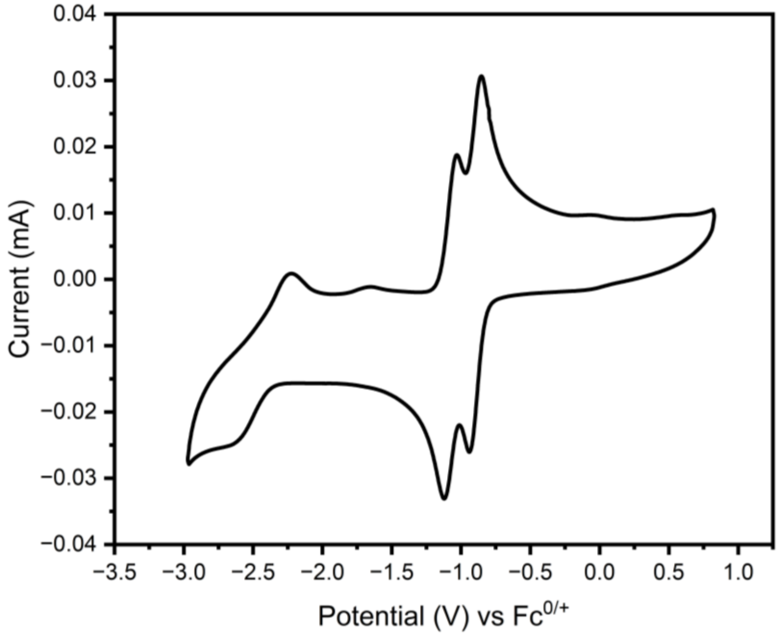

Figure S32. Cyclic voltammogram of **4-Th(W_4Mo)$_2$**. The data was acquired in MeCN with 0.1 M TBA(PF_6) supporting electrolyte, 1 mM of cluster, and a scan rate of 200 mV s^{-1}. The open circuit potential (OCP) was −0.80 V.

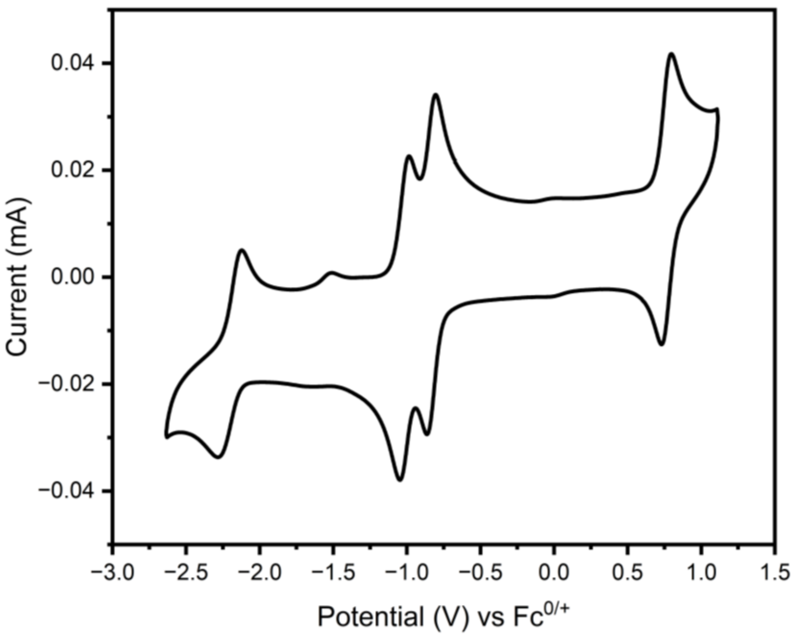

Figure S33. Cyclic voltammogram of **5-U(W$_4$Mo)$_2$**. The data was acquired in MeCN with 0.1 M TBA(PF$_6$) supporting electrolyte, 1 mM of cluster, and a scan rate of 200 mV s^{-1}. The open circuit potential (OCP) was −0.67 V.

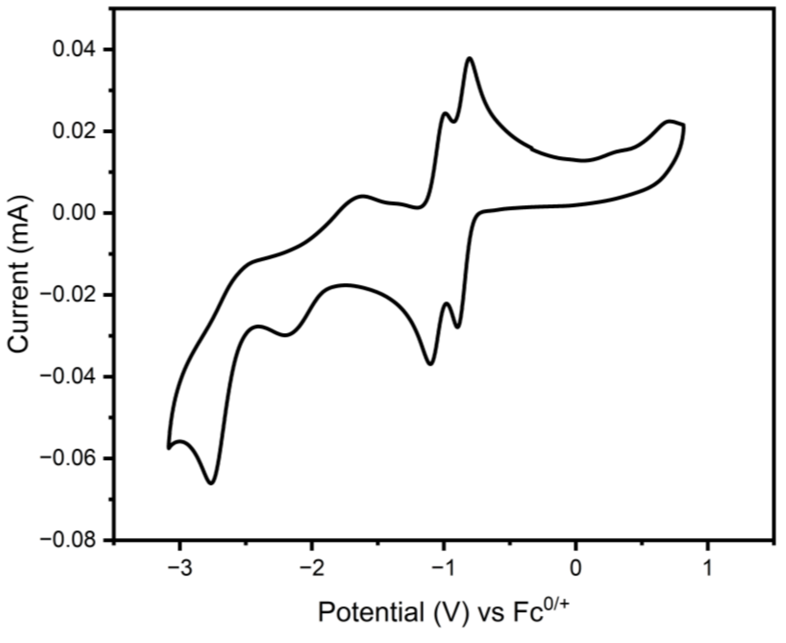

Figure S34. Cyclic voltammogram of **6-Np(W$_4$Mo)$_2$**. The data was acquired in MeCN with 0.1 M TBA(PF$_6$) supporting electrolyte, 1 mM of cluster, and a scan rate of 200 mV s^{-1}. The open circuit potential (OCP) was −0.33 V.

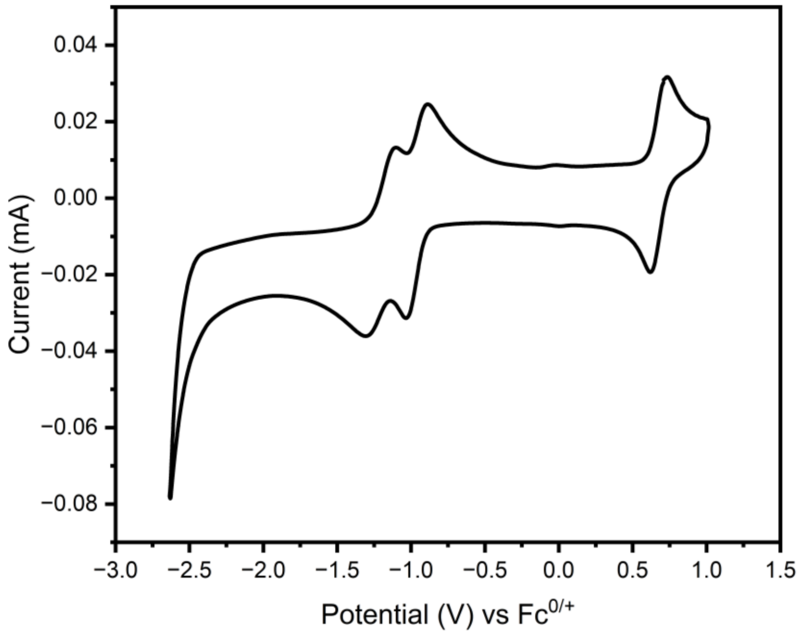

Figure S35. Cyclic voltammogram of **7-U(W$_4$Mo)$_2$**. The data was acquired in DCM with 0.1 M TBA(PF$_6$) supporting electrolyte, 1 mM of cluster, and a scan rate of 200 mV s^{-1}. The open circuit potential (OCP) was 0.71 V (the $E_{1/2}$ of the oxidation event is 0.68 V).

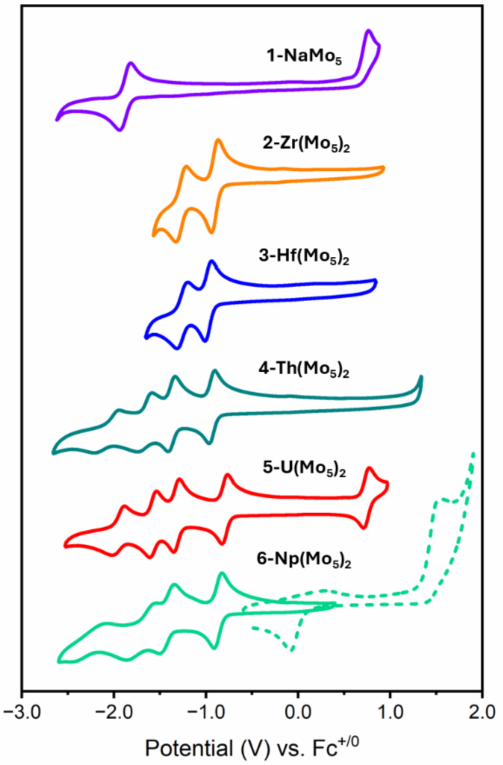

Figure S36. Cyclic voltammograms of the series of sandwich complexes with formula (TBA)$_2$[M{Mo$_5$O$_{13}$(OMe)$_4$NO}$_2$] (M = Zr(IV), Hf(IV), Th(IV), U(IV), and Np(IV)), and (TBA)$_2$[Mo$_5$O$_{13}$(OMe)$_4$NO][Na(MeOH)], previously reported by our group.[1, 5]

S6 Single Crystal X-ray Diffraction

Table S1. Crystallographic parameters for **4-Th(W_4Mo)$_2$** and **5-U(W_4Mo)$_2$**.

	**4-Th(W_4Mo)$_2$**	**5-U(W_4Mo)$_2$**
Empirical formula	$C_{45.01}H_{106.53}Mo_2N_5O_{36.75}ThW_8$	$C_{44}H_{104}Mo_2N_5O_{36.50}UW_8$
Formula weight	3200.75	3188.03
Temperature	100.00(10) K	100.00(10) K
Wavelength	1.54184 Å	0.56087 Å
Crystal system	Monoclinic	Monoclinic
Space group	$P2_{1/c}$	$P2_{1/c}$
Unit cell dimensions	*a* = 21.91739(10) Å<br>*b* = 15.05553(7) Å<br>*c* = 24.14831(10) Å<br>α = 90°<br>β = 92.7883(4)°<br>γ = 90°	a = 21.8158(3) Å<br>b = 15.0520(2) Å<br>c = 24.2167(4) Å<br>α = 90°<br>β = 92.787(2)°<br>γ = 90°
Volume	7958.98(6) $Å^3$	7942.7(2) $Å^3$
Z	4	4
Independent reflections	16256	19681
Goodness-of-fit on F^2	1.157	1.111
Final R indices<br>[*I*>2sigma(*I*)]	*R1 = 0.0377*<br>*wR2 = 0.0780*	*R*1 = 0.0317<br>*wR*2 = 0.0650

Table S2. Crystallographic parameters for **6-Np(W$_4$Mo)$_2$** and **7-U(W$_4$Mo)$_2$**.

	**6-Np(W$_4$Mo)$_2$**	**7-U(W$_4$Mo)$_2$**
Empirical formula	$C_{44.65}H_{105.62}Mo_2N_5O_{36.66}NpW_8$	$C_{26.64}H_{65.29}Cl_{5.29}Mo_2N_3O_{36}UW_8$
Formula weight	3198.98	3091.87
Temperature	150.00(10) K	100.00(10) K
Wavelength	0.71073 Å	0.71073 Å
Crystal system	Monoclinic	Monoclinic
Space group	$P2_{1/c}$	$P2_{1/n}$
Unit cell dimensions	*a* = 21.8013(13) Å<br>*b* = 15.0773(9) Å<br>*c* = 24.1394(12) Å<br>α = 90°<br>β = 92.832(2)°<br>γ = 90°	a = 16.4186(3) Å<br>b = 21.1179(3) Å<br>c = 19.0748(3) Å<br>α = 90°<br>β = 103.2084(17)°<br>γ = 90°
Volume	7925.0(8) Å^3	6438.75(19) Å^3
Z	4	4
Independent reflections	26329	21174
Goodness-of-fit on F^2	1.025	1.071
Final R indices [*I*>2sigma(*I*)]	*R1 = 0.0479*<br>*wR2 = 0.1044*	*R*1 = 0.0383<br>*wR*2 = 0.0739

Table S3: Average bond length data for the structures discussed. All values in Å. A schematic is given below to highlight bond assignments.

	4-Th(W_4Mo)$_2$	5-U(W_4Mo)$_2$	6-Np(W_4Mo)$_2$	7-U(W_4Mo)$_2$
**M-O**	2.409	2.364	2.353	2.282
**W-O-W**	1.916	1.913	1.918	1.912
**W=O**	1.692	1.706	1.708	1.707
**W-O-M**	1.789	1.786	1.784	1.800
**W-O(μ$_5$)**	2.345	2.332	2.335	2.331
**Mo-O(μ$_5$)**	2.125	2.116	2.120	2.101
**Mo-O (eq)**	2.005	2.011	2.013	2.014
**W-O (ax)**	2.191	2.195	2.186	2.156
**M$_5$O- μ$_5$O**	6.924	6.870	6.831	6.749
**O-O**	3.093	3.019	2.987	2.894
**Mo-NO**	1.764	1.769	1.756	1.766

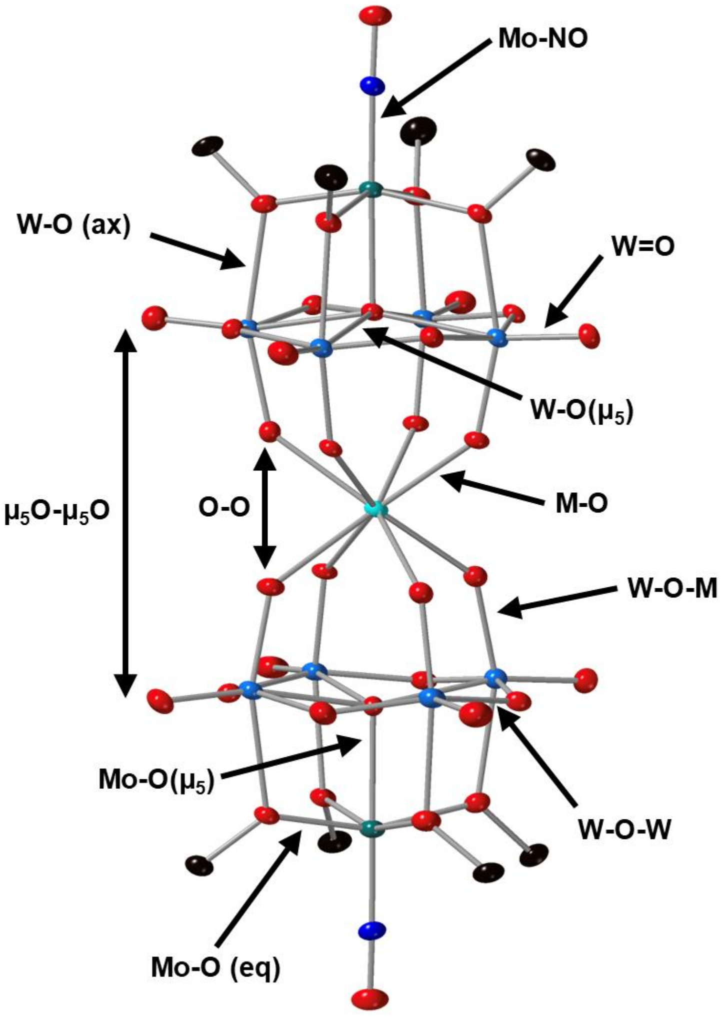

S7. Computational calculations

7.1 Additional Computational Details

Individual bands predicted by TD-DFT calculations were convoluted assuming a gaussian shape:

$$\varepsilon(\tilde{\nu}) = \sum_{i=1}^{N} \frac{\sqrt{\pi} \cdot e^2 \cdot N_A}{\ln(10) \cdot c^2 \cdot m_e} \frac{f_i}{\sigma} \exp\left[-\left(\frac{\tilde{\nu} - \tilde{\nu}_i}{\sigma}\right)^2\right] \qquad \text{S1}$$

where f_i is the oscillation strength of the excitation *i* (dimensionless), sigma is the half-width of the Gaussian band, and $\tilde{\nu}_i$ is the exitation energy (in wavenumbers) od the electronic excitation *i*. e^2, N_A, *c*, and m_e account for the elementary charge, Avogadro's number, speed of light, and electron's mass, respectively. **Figures S53-S61** show the simulated UV-Vis spectra and **Tables S6-S16** show the relevant transitions obtained from TD-DFT calculations. Table S4 collects σ utilized for each system investigated.

Table S4. Values of the half-width of the Gaussian band (σ) utilized for each system.

	**1-NaMo$_5$**	**2-Zr(Mo$_5$)$_2$**	**3-Hf(Mo$_5$)$_2$**	**4-Th(Mo$_5$)$_2$**
σ	0.180 eV	0.165 eV	0.165 eV	0.130 eV
	**1-NaW$_4$Mo**	**2-Zr(W$_4$Mo)$_2$**	**3-Hf(W$_4$Mo)$_2$**	**4-Th(W$_4$Mo)$_2$**
σ	0.180 eV	0.180 eV	0.180 eV	0.160 eV

The reduction (ionization) potentials of **1-NaMo$_5$** and **1-NaW$_4$Mo** were evaluated using DFT. To do so, geometry optimizations on the one-electron reduces species $\{[Mo_5O_{13}(OMe)_4NO][Na(MeOH)]\}^{3-}$ and $\{[W_4O_{13}(OMe)_4MoNO][Na(MeOH)]\}^{3-}$. The reduction potential (using ferrocene as reference electrode) was calculated via the following protocol [6-9]

$$E^{\circ}_{\frac{1}{2}}(\text{vs. ref}) = \frac{G(ox) - G(red)}{F} - E^{\circ}_{abs}(\text{ref}) \qquad \text{S2}$$

where G(ox) and G(red) are the Gibbs free energies of oxidated and reduced species, respectively, and F is the faraday constant. Reduction potentials were calculated with respect to the ferrocene electrode, Fc/Fc^+, henceforth the last term in eq. S2 (E(ref) = 4.809 V).

G is evaluated through DFT via

$$G = \varepsilon_{DFT} + \varepsilon_{ZPE} + G_{th}$$

where ε_{DFT} is the final SCF energy, ε_{ZPE} is the zero point correction, and G_{th} is the thermal corrections (to 298 K) for the Gibbs free energy.

7.2 Benchmark Calculations

Prior to the production calculations, we performed an extensive test varying the DFT functional and basis sets (for the lighter elements C, O, N, and H) on **3-Hf(Mo$_5$)$_2$**. We kept the Stuttgart pseudopotential and basis set for Hf, as well as the convergence parameters mentioned in the main text. **Table S5** depicts structural parameters obtained with the various theoretical models investigated.

Table S5. Structural results obtained with the various basis sets and DFT functionals tested. Averaged results for the equivalent Mo-N, N-O, and Metal-O bonds are reported, alongside the percentual error with regard to the experimental values [5]

Basis (C,N,O,H)	DFT	Mo-N (Exp.)	Mo-N (avg.)		Metal-O (Exp.)	Metal-O (avg.)		N-O (Exp.)	N-O (avg.)	
			r	Error		r	Error		r	Error
6-311g(2d,2p)	PBE0	1.773	1.764	-0.496%	2.191	2.217	1.191%	1.201	1.182	-1.624%
6-311g(2d,2p)	PBE0-D	1.773	1.763	-0.564%	2.191	2.216	1.118%	1.201	1.182	-1.599%
6-311g(2d,2p)	wB97X-D	1.773	1.755	-1.043%	2.191	2.225	1.565%	1.201	1.182	-1.549%
6-311g(2d,2p)	BP86	1.773	1.770	-0.164%	2.191	2.216	1.127%	1.201	1.185	-1.299%
6-311g(2d,2p)	M06L	1.773	1.780	0.395%	2.191	2.230	1.771%	1.201	1.195	-0.475%
6-311g(2d,2p)	B3LYP	1.773	1.780	0.372%	2.191	2.234	1.963%	1.201	1.190	-0.908%
6-311g(2d,2p)	PBE	1.773	1.791	0.993%	2.191	2.231	1.835%	1.201	1.205	0.366%
def2-tzvp	PBE0	1.773	1.760	-0.711%	2.191	2.214	1.063%	1.201	1.180	-1.740%
def2-tzvp	PBE0-D	1.773	1.759	-0.767%	2.191	2.213	0.986%	1.201	1.180	-1.715%
def2-tzvp	wB97X-D	1.773	1.751	-1.224%	2.191	2.222	1.424%	1.201	1.180	-1.732%
def2-tzvp	BP86	1.773	1.767	-0.361%	2.191	2.213	1.000%	1.201	1.183	-1.474%
def2-tzvp	M06L	1.773	1.778	0.259%	2.191	2.224	1.520%	1.201	1.189	-0.991%
def2-tzvp	PBE	1.773	1.787	0.807%	2.191	2.230	1.775%	1.201	1.203	0.200%

7.3 Optimized XYZ Coordinates from the DFT Calculations

The external file Na-Th.xyz gives the optimized coordinates collected from the calculations reported in the main text of this manuscript, in the xyz format. These were obtained under the B3LYP level, paired with the Stuttgart relativistic small-core pseudopotential alongside its associated basis set (transition metals and thorium), and the 6-311G(2d,2f) basis set for the lighter elements. Optimized coordinates in the .xyz file follow the labelling used in this manuscript. The reduced species of **1-NaMo$_5$** and **1-NaW$_4$Mo** are provided and labeled as $\{[Mo_5O_{13}(OMe)_4NO][Na(MeOH)]\}^{3-}$ and $\{[W_4O_{13}(OMe)_4MoNO][Na(MeOH)]\}^{3-}$, respectively. The three electron reduced species of **4-Th(W$_4$Mo)$_2$** is provided in duplet and quartet spin multiplicities and are labeled $[Th\{W_4O_{13}(OMe)_4MoNO\}_2]^{5-}$ (duplet) and $[Th\{W_4O_{13}(OMe)_4MoNO\}_2]^{5-}$ (quartet), respectively. For additional details, we refer the reader to the Computational Methods section in the main text.

7.4 Selected Molecular Orbitals and Molecular Orbital Diagram

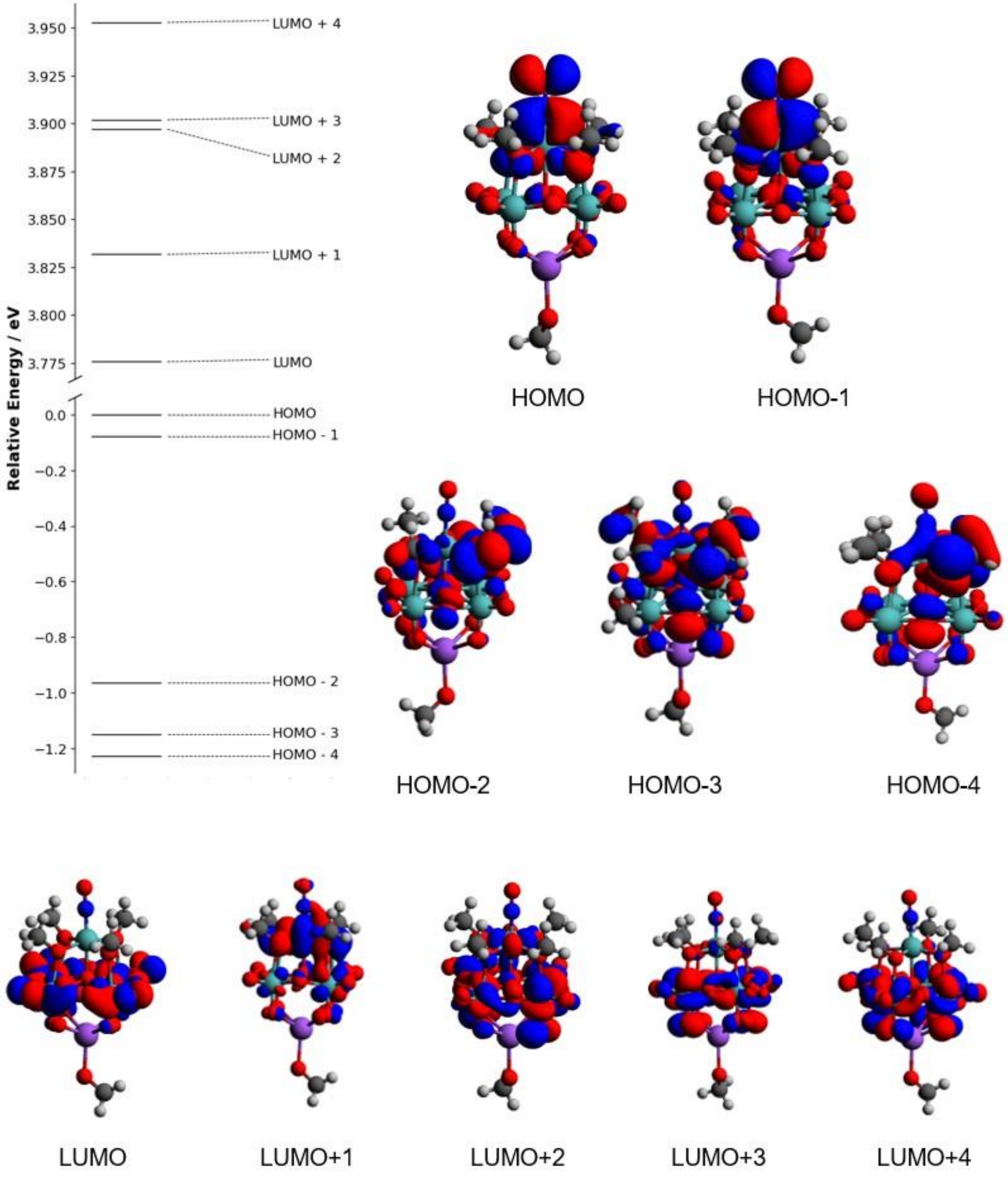

Figure S37. Energies and orbital diagrams for **1-NaMo₅**.

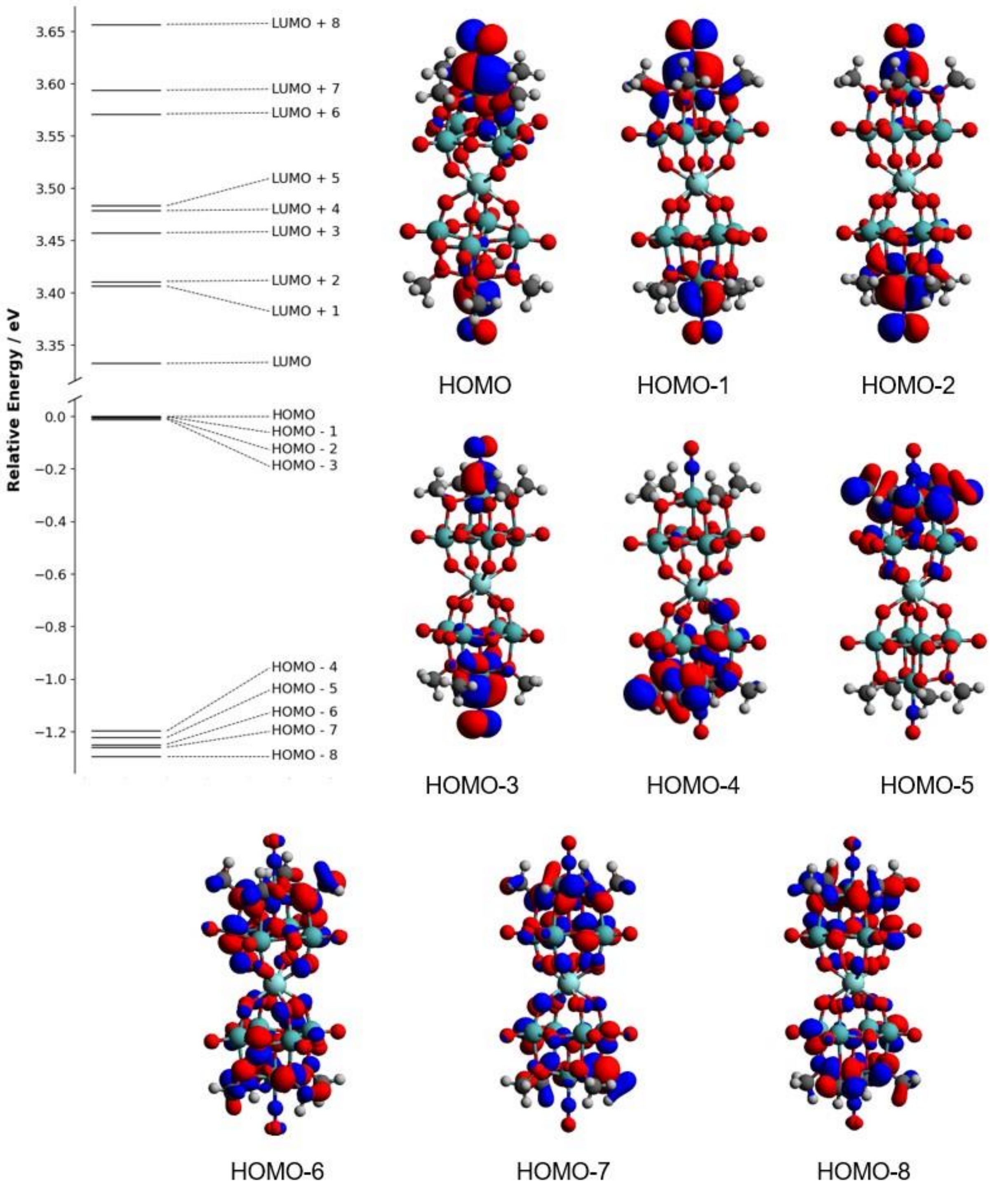

Figure S38. Energies and orbital diagrams of the HOMO to HOMO-8 of **2-Zr(Mo_5)$_2$**.

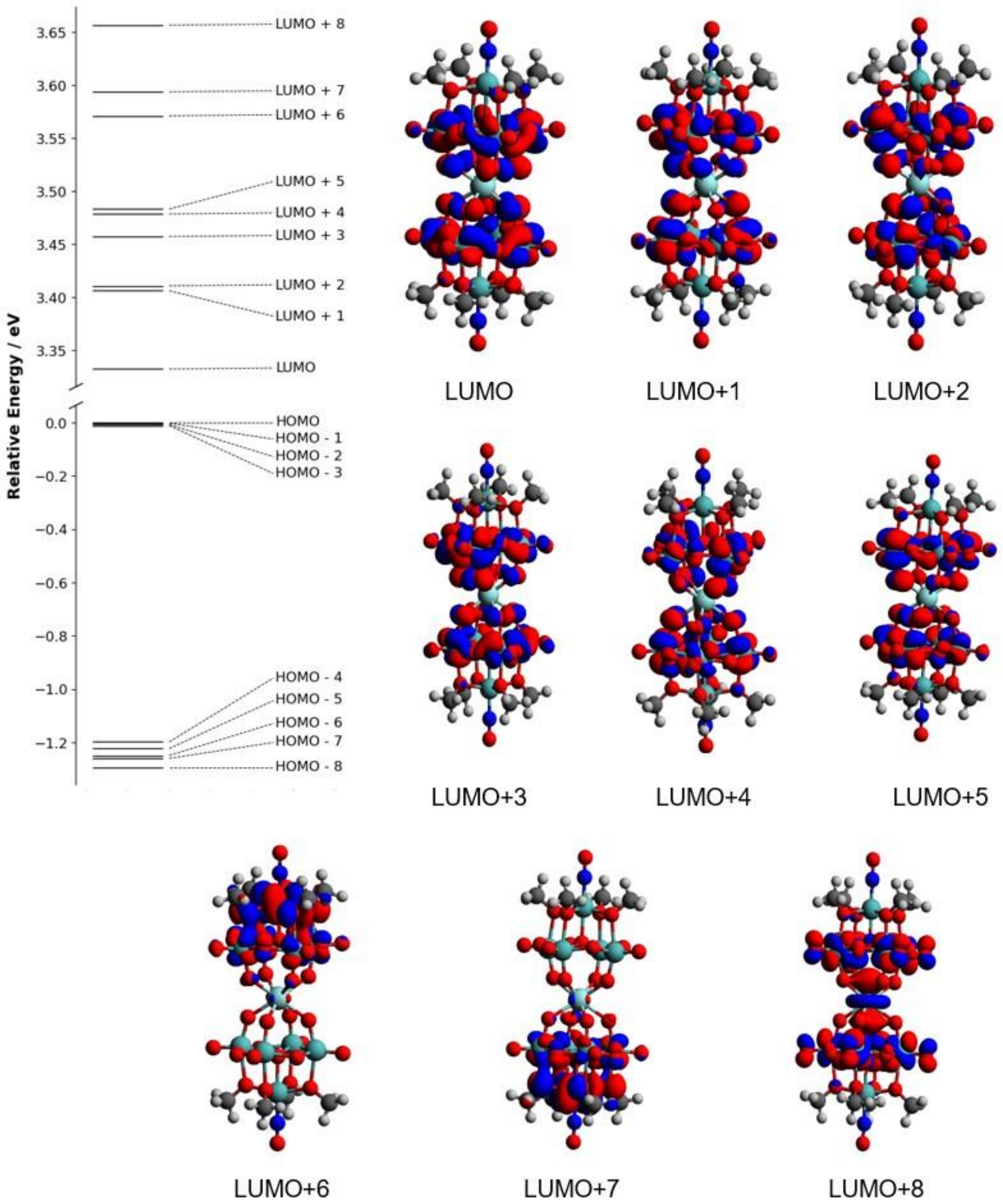

Figure S39. Energies and orbital diagrams of the LUMO to LUMO+8 of **2-Zr(Mo$_5$)$_2$**.

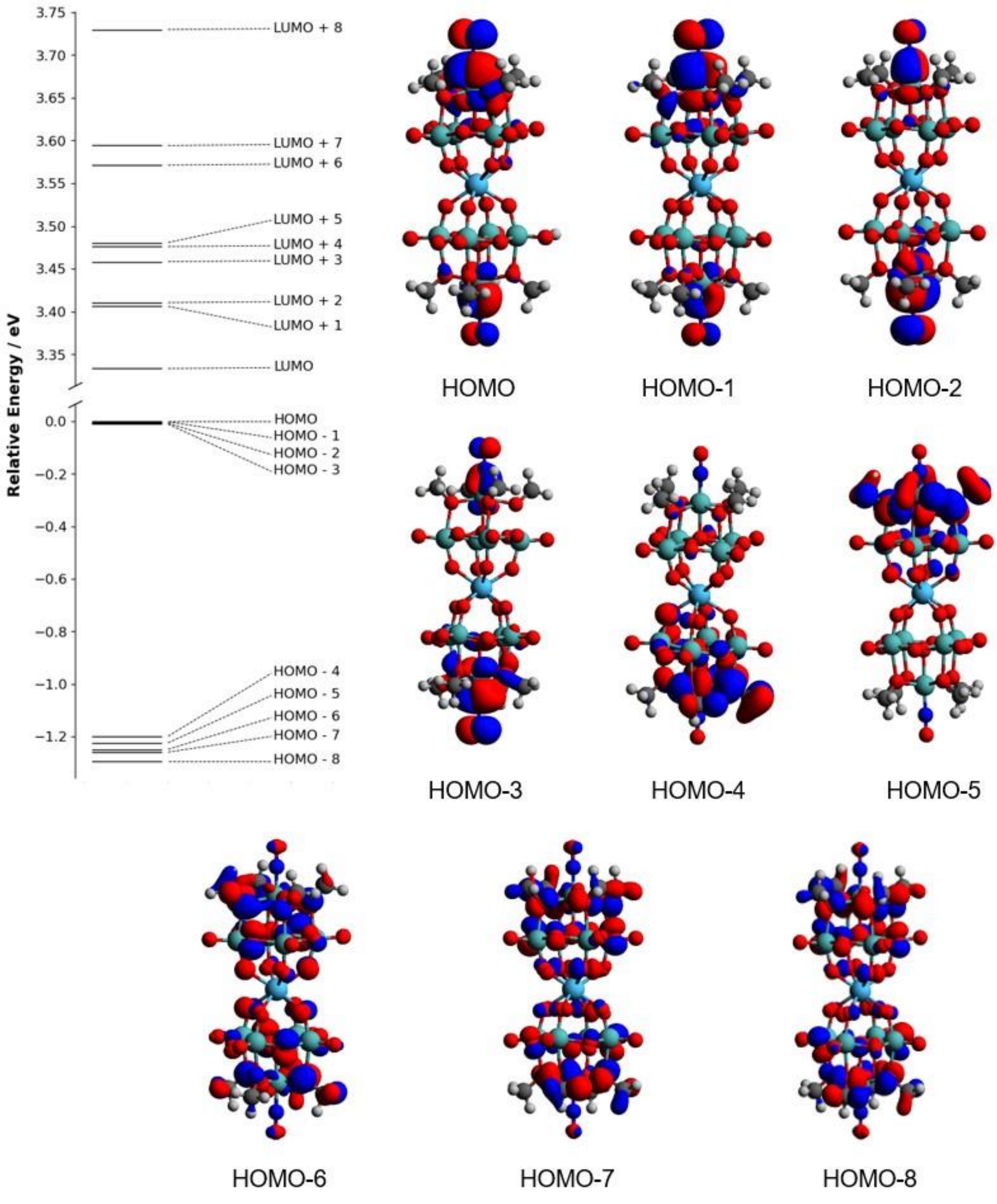

Figure S40. Energies and orbital diagrams of the HOMO to HOMO-8 of **3-Hf(Mo$_5$)$_2$**.

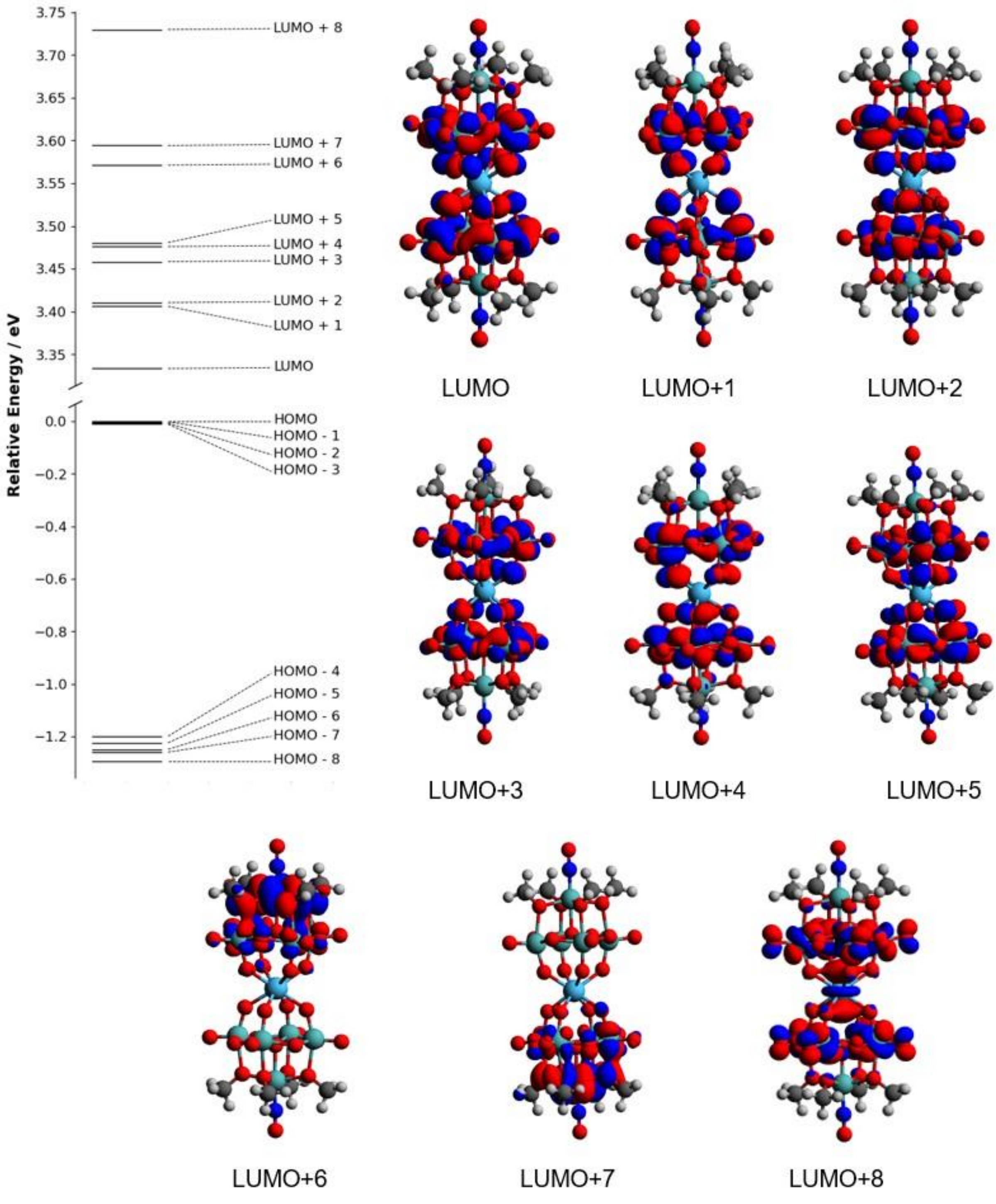

Figure S41. Energies and orbital diagrams of the LUMO to LUMO+8 of **3-Hf(Mo$_5$)$_2$**.

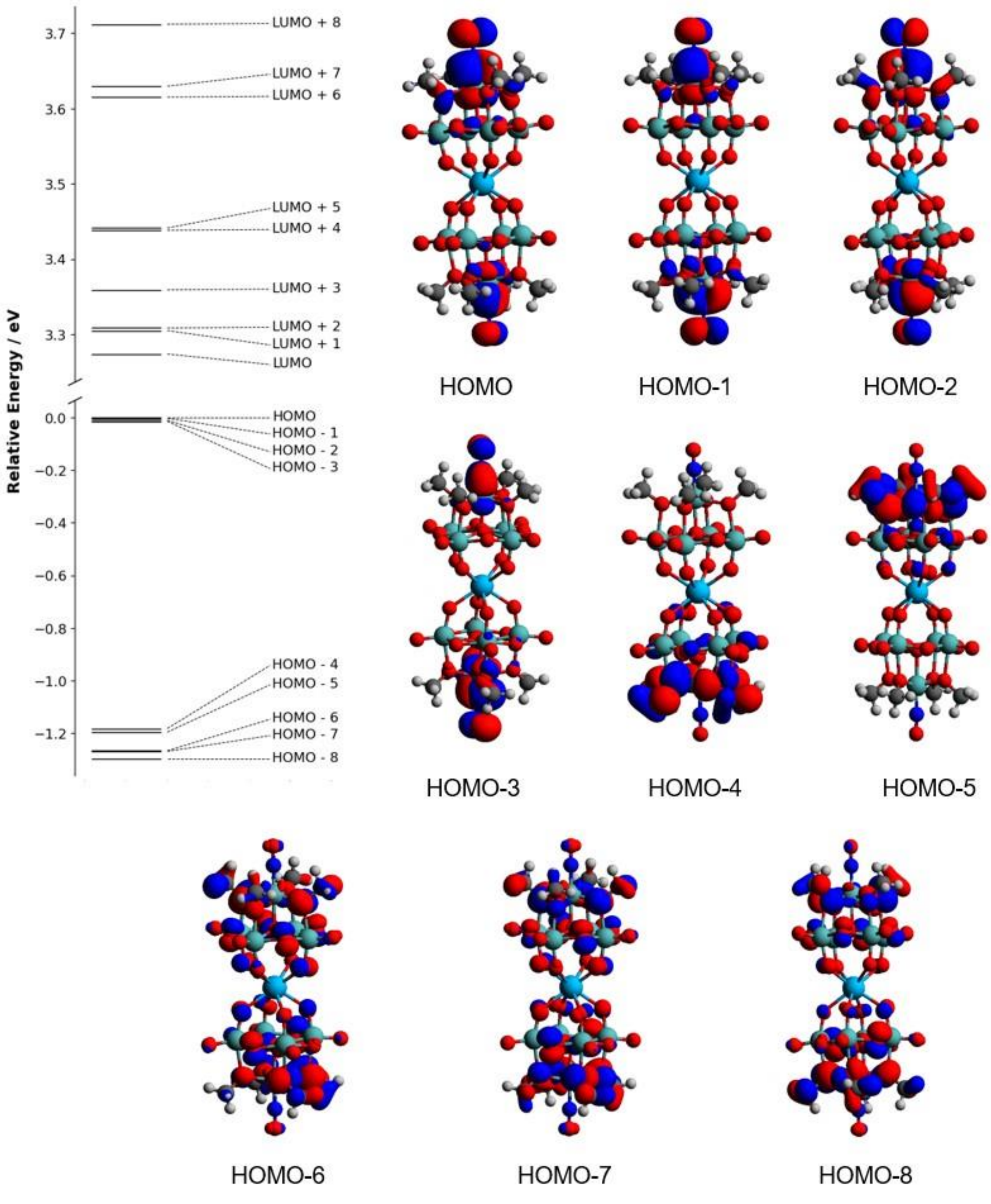

Figure S42. Energies and orbital diagrams of the HOMO to HOMO-8 of **4-Th(Mo$_5$)$_2$**.

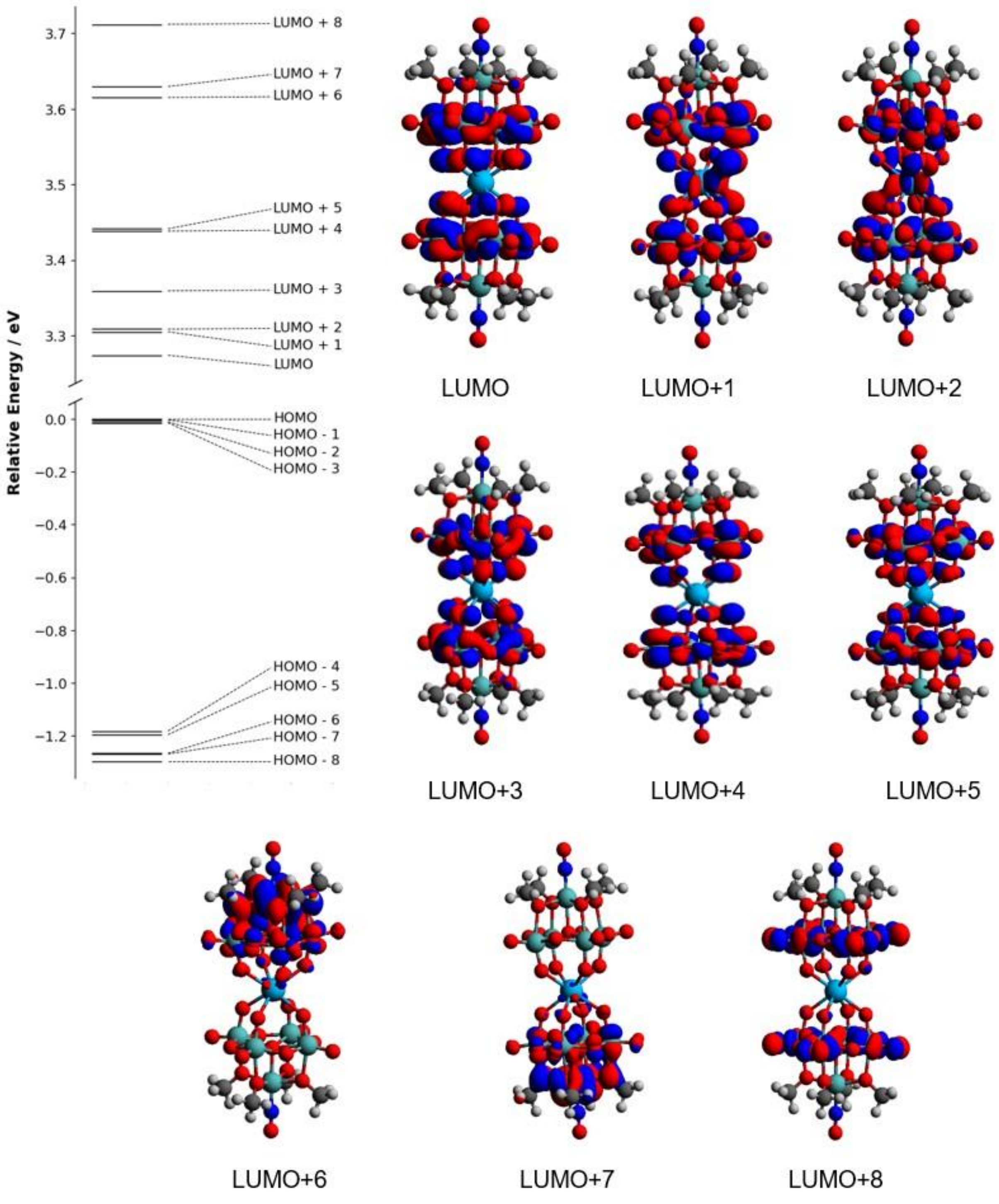

Figure S43. Energies and orbital diagrams of the LUMO to LUMO+8 of **4-Th(Mo$_5$)$_2$**.

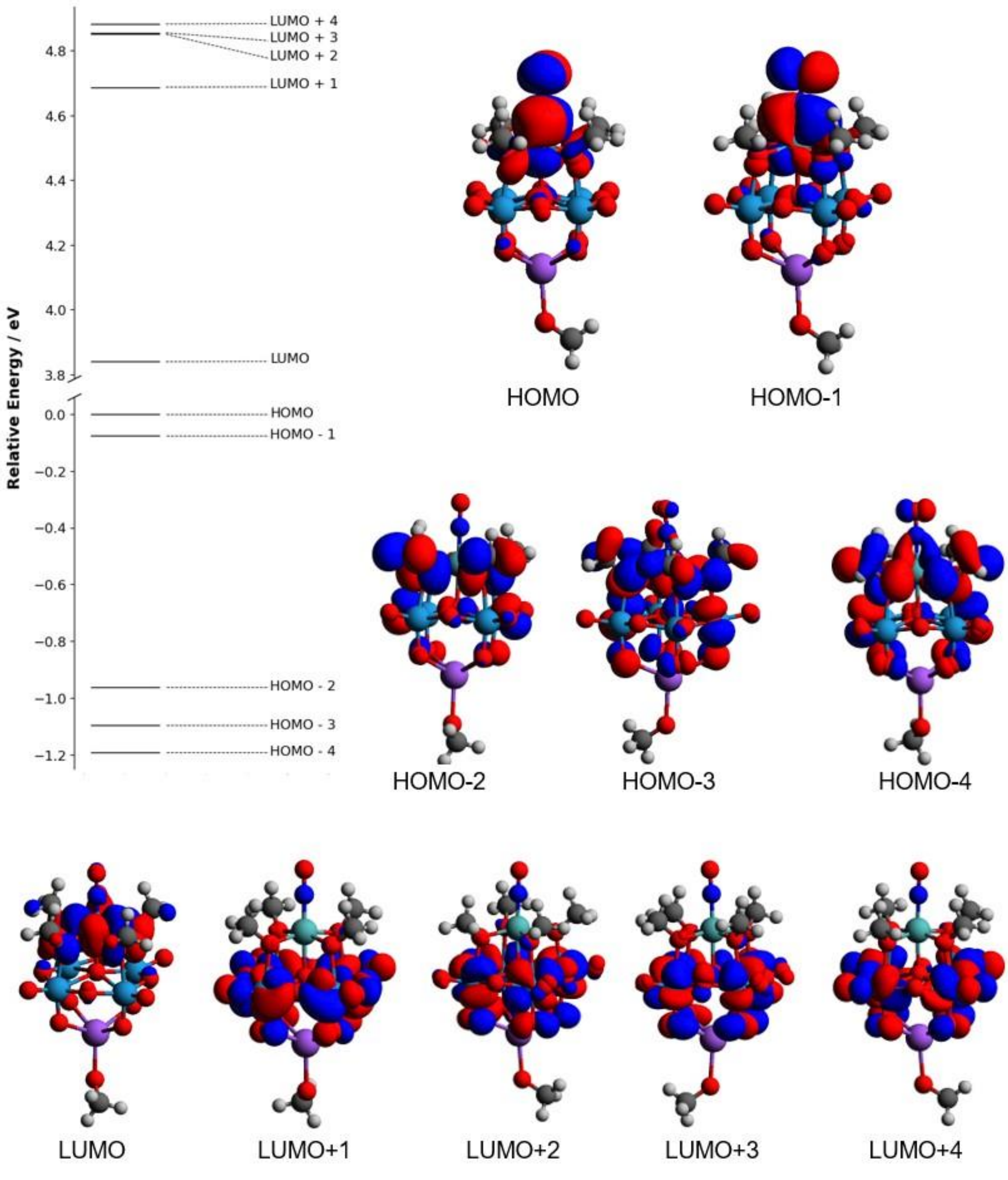

Figure S44. Energies and orbital diagrams for **1-NaW$_4$Mo**.

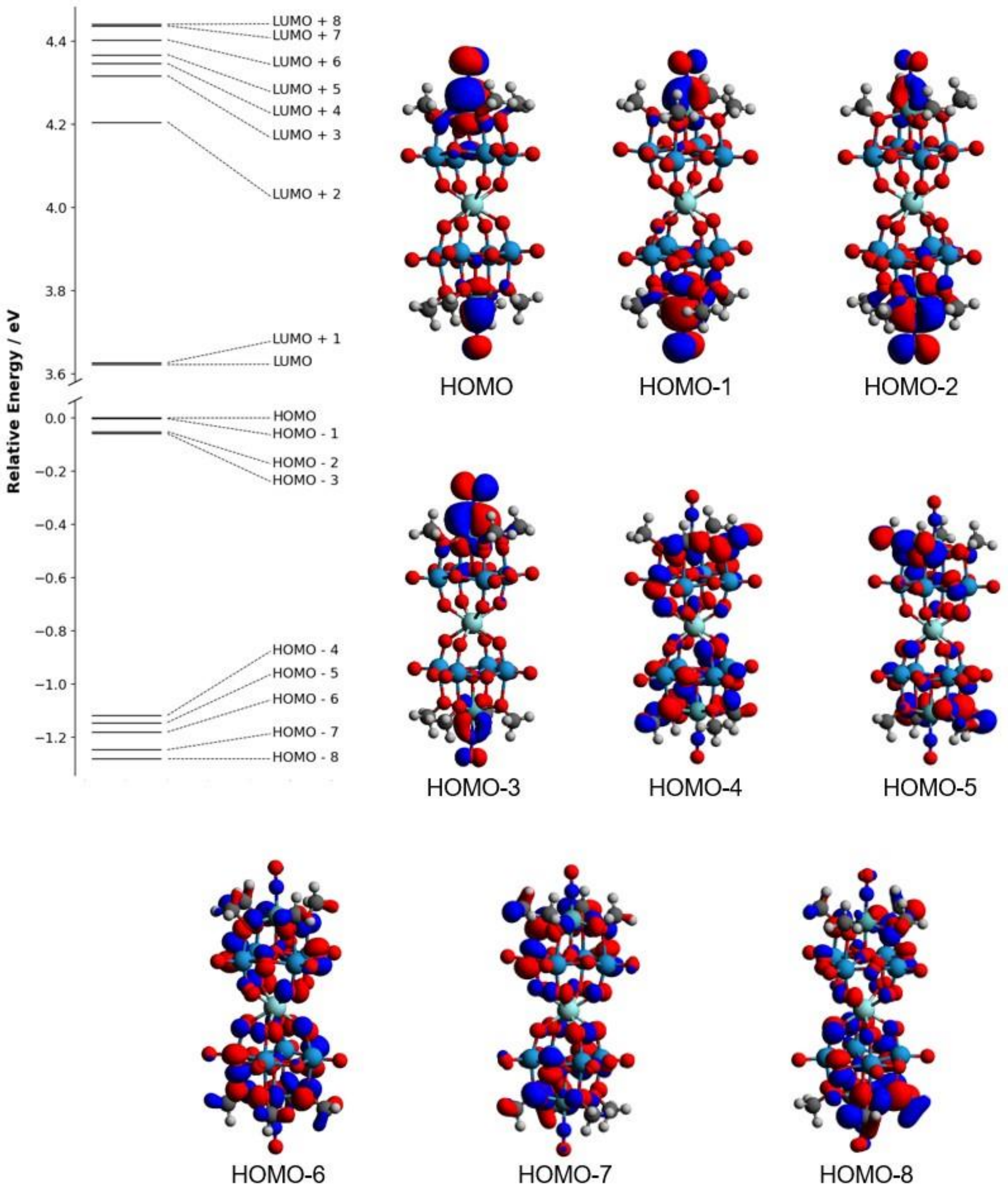

Figure S45. Energies and orbital diagrams of the HOMO to HOMO-8 of **2-Zr(W_4Mo)$_2$**.

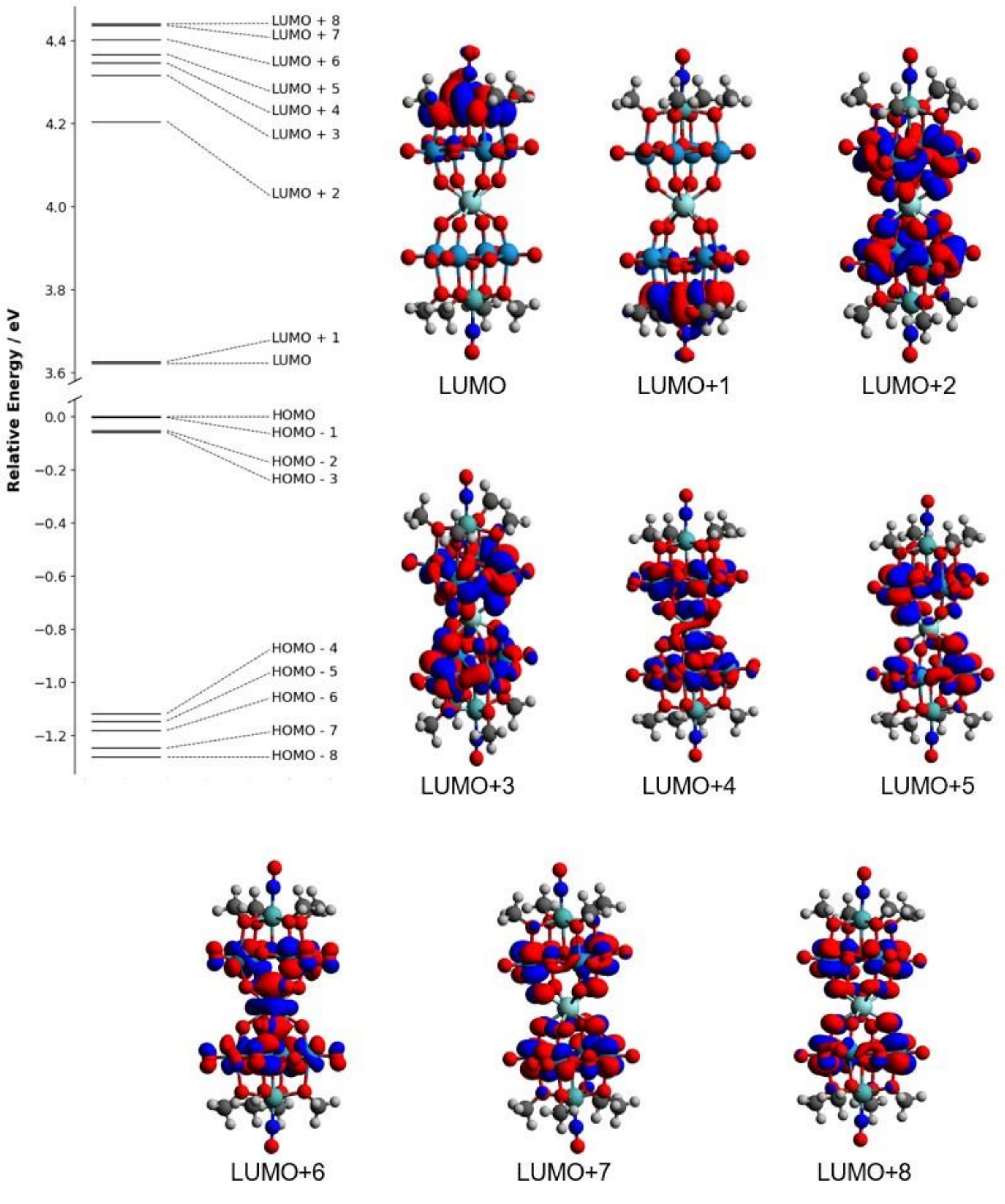

Figure S46. Energies and orbital diagrams of the LUMO to LUMO+8 of **2-Zr(W$_4$Mo)$_2$**.

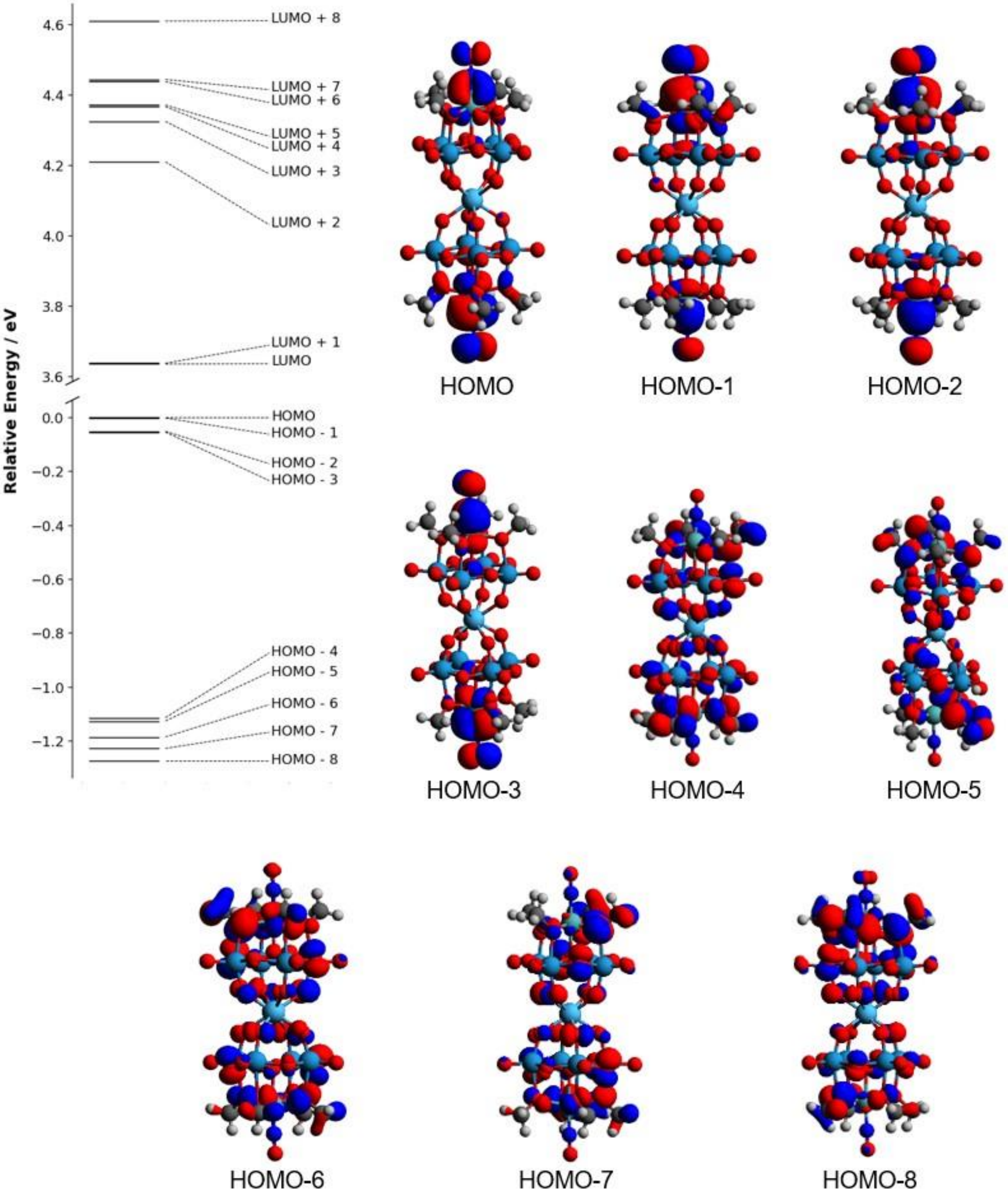

Figure S47. Energies and orbital diagrams of the HOMO to HOMO-8 of **3-Hf(W$_4$Mo)$_2$**.

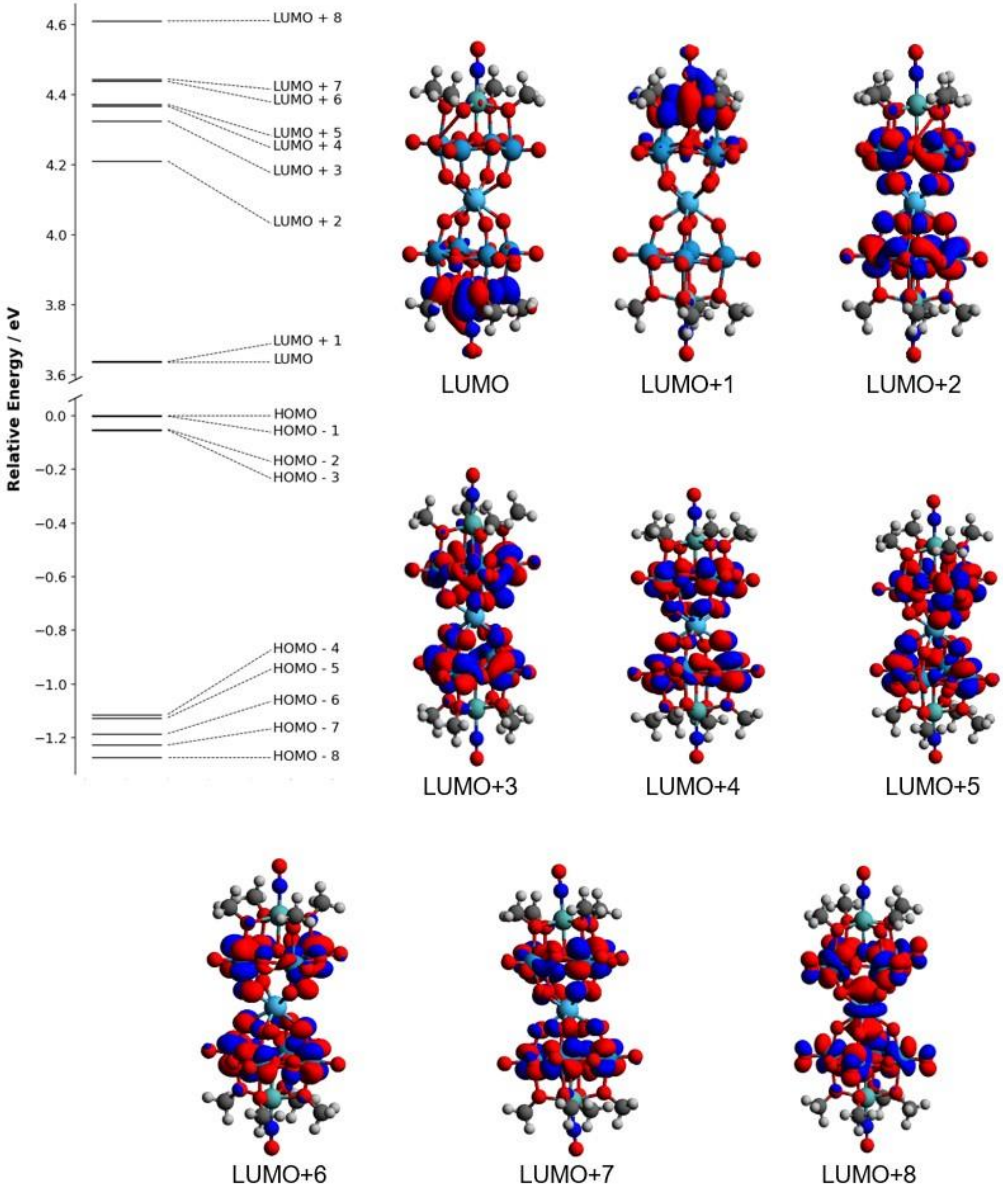

Figure S48. Energies and orbital diagrams of the LUMO to LUMO+8 of **3-Hf(W$_4$Mo)$_2$**.

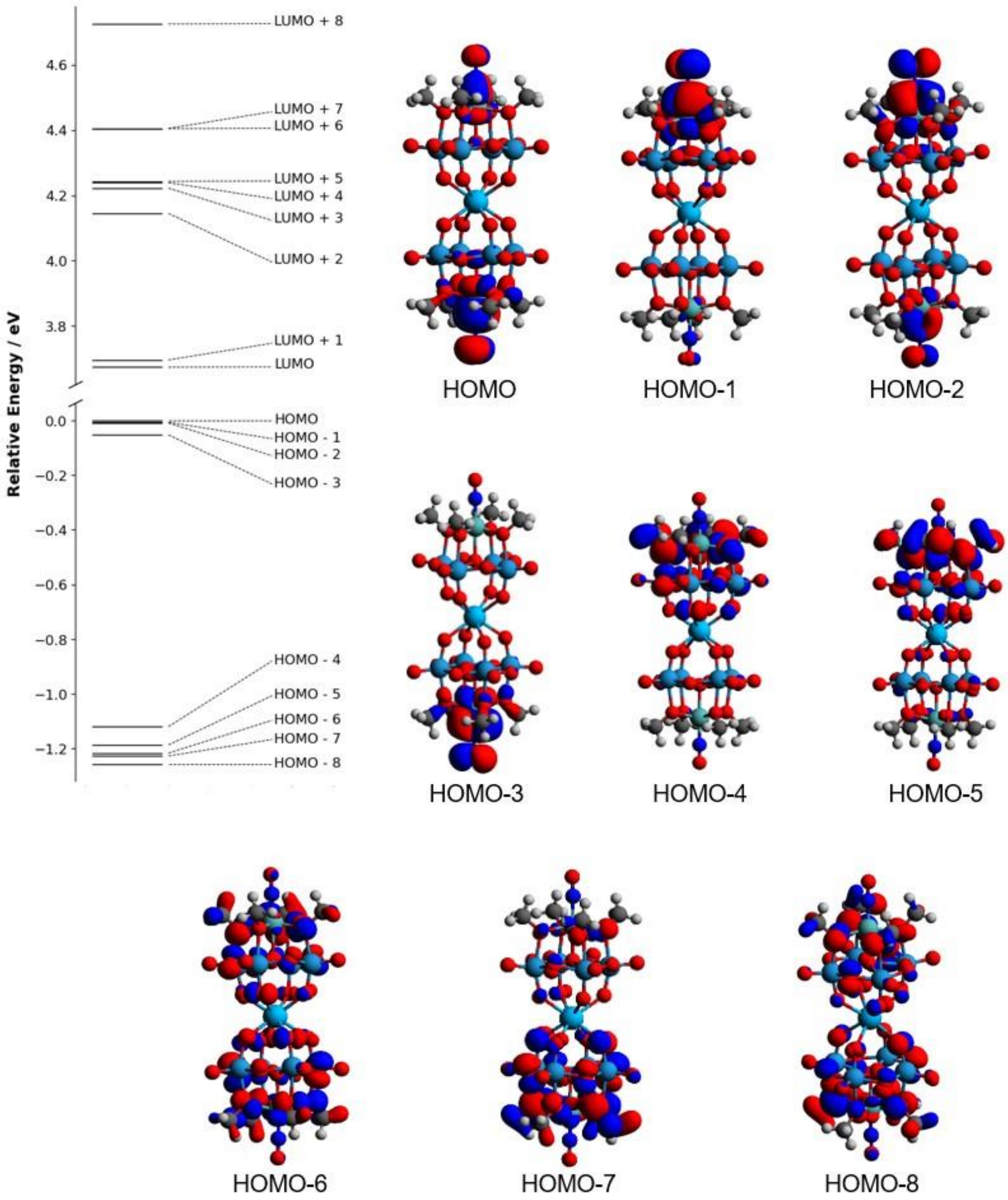

Figure S49. Energies and orbital diagrams of the HOMO to HOMO-8 of **4-Th(W$_4$Mo)$_2$**.

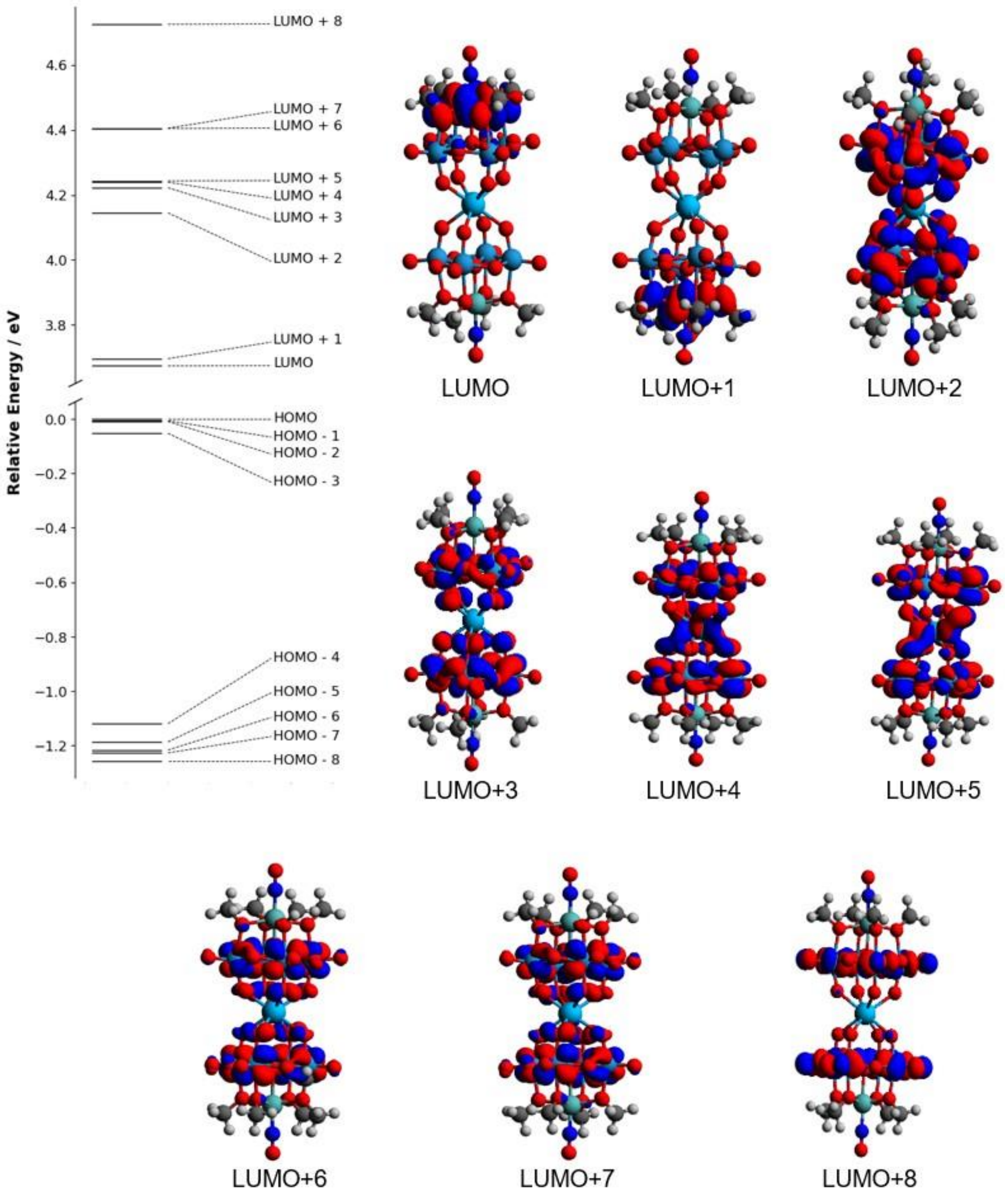

Figure S50. Energies and orbital diagrams of the LUMO to LUMO+8 of **4-Th(W$_4$Mo)$_2$**.

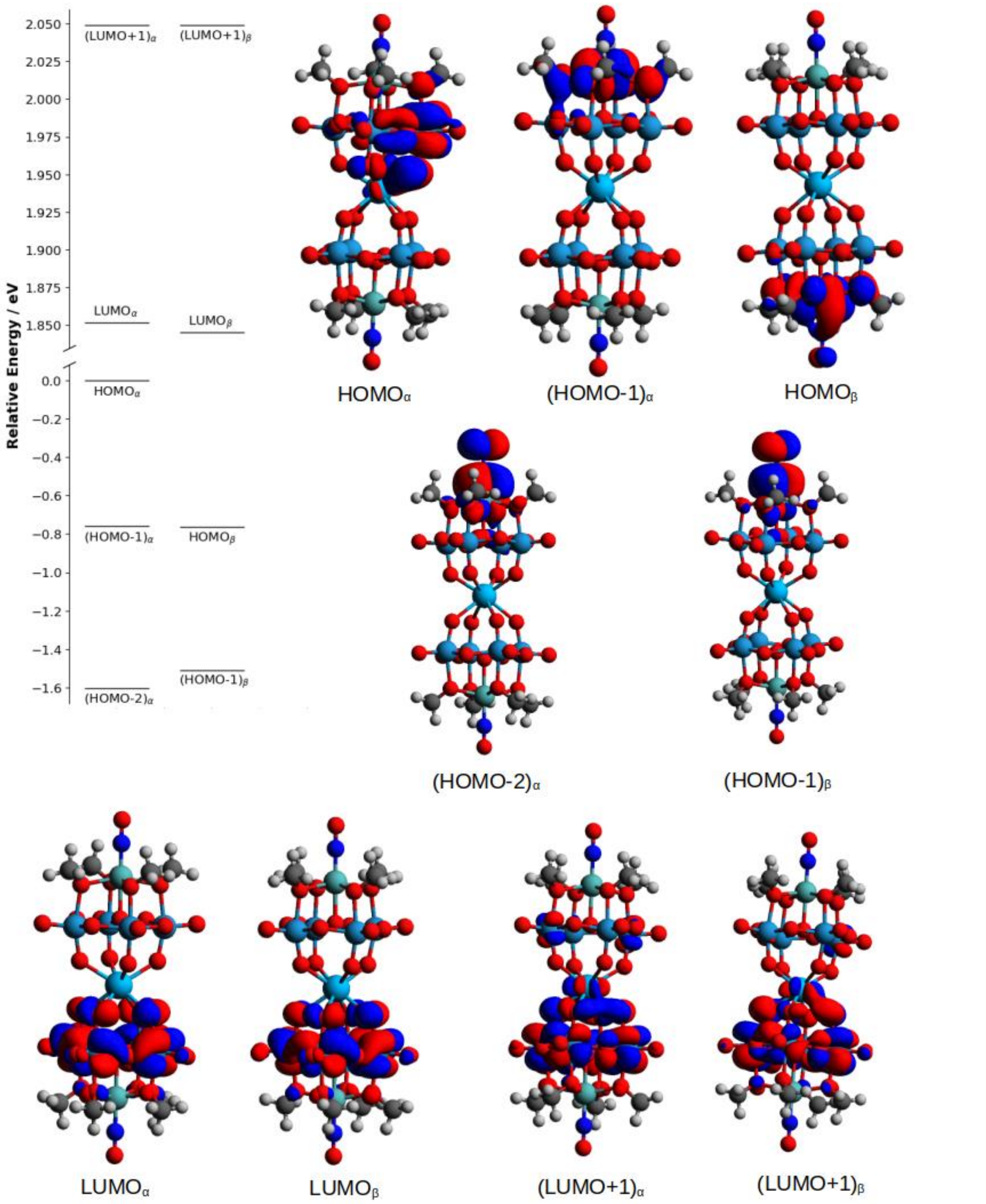

Figure S51. Energies and orbital diagrams of the 3rd reduced state of **4-Th(W_4Mo)$_2$** in the duplet spin multiplicity. As this reduced species is an open-shell system, α and β orbitals are indicated.

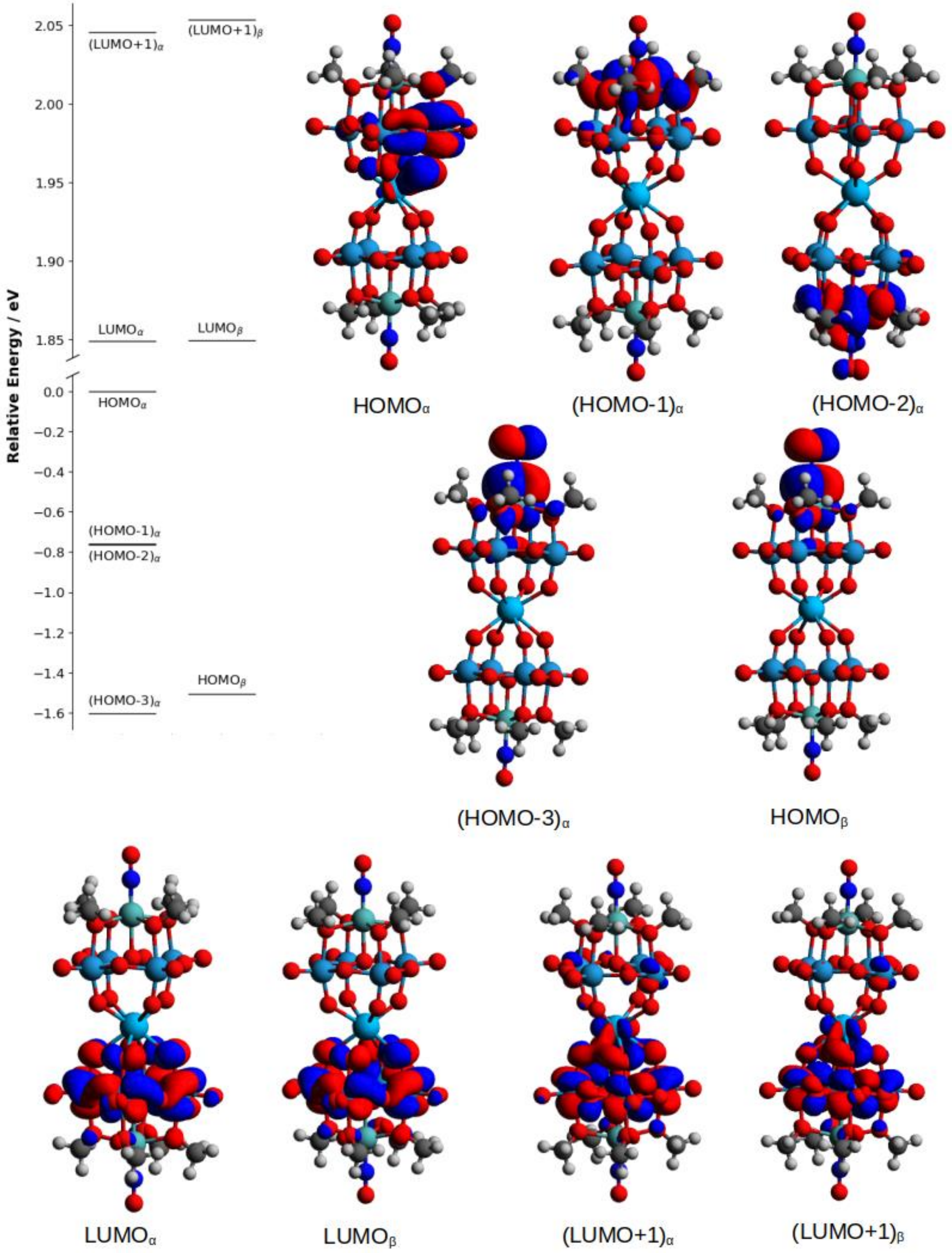

Figure S52. Energies and orbital diagrams of the 3rd reduced state of **4-Th(W$_4$Mo)$_2$** in the quartet spin multiplicity. As this reduced species is an open-shell system, α and β orbitals are indicated

7.5 TD-DFT Simulated UV-Spectra

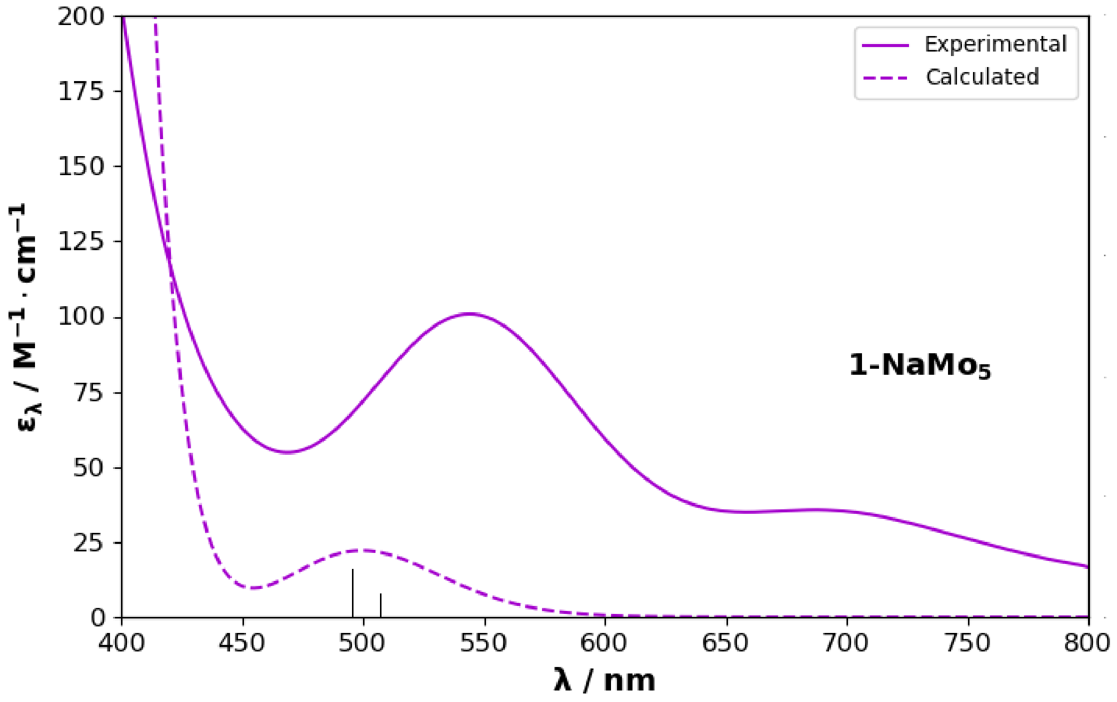

Figure S53. Simulated UV-Vis spectrum of **1-NaMo$_5$**.

Table S6. Orbitals that are involved in the relevant transitions observed in the simulated UV-Vis spectrum of **1-NaMo$_5$**.

Transition Wavelength	Initial State	Final State	Contribution
495.90 nm	HOMO-1	LUMO+1	88.35 %
	HOMO-1	LUMO+2	6.06 %
507.41 nm	HOMO	LUMO+1	87.89 %
	HOMO	LUMO+2	6.16 %

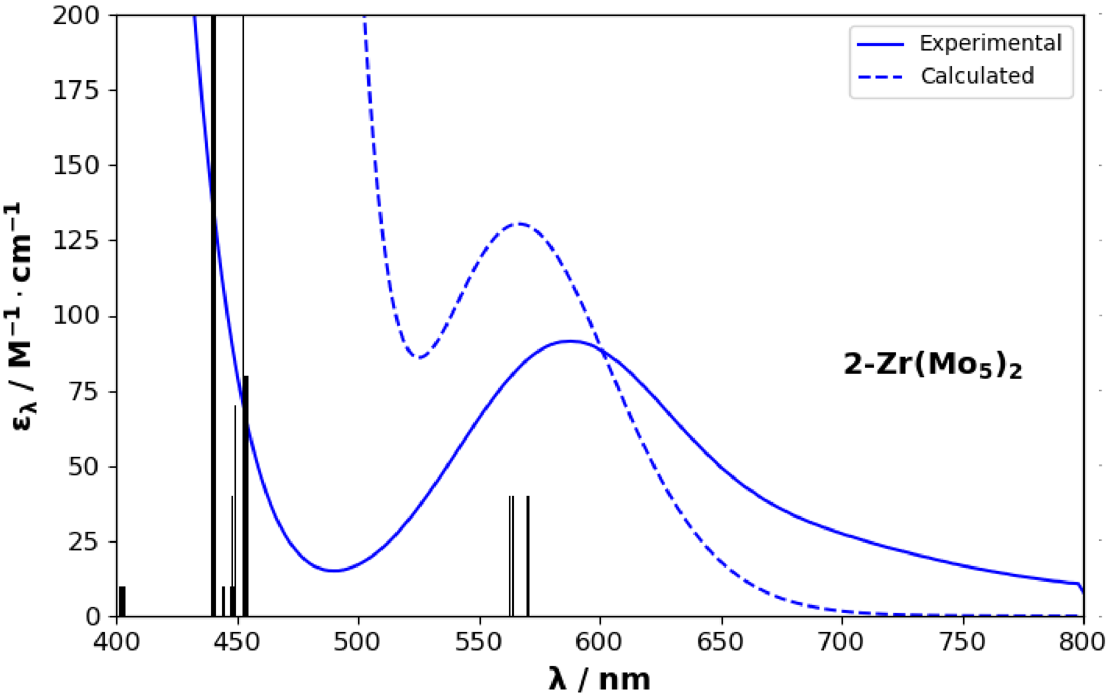

Figure S54. Simulated UV-Vis spectrum of **2-Zr(Mo$_5$)$_2$**.

Table S7. Orbitals that are involved in the relevant transitions observed in the simulated UV-Vis spectrum of **2-Zr(Mo_5)$_2$**.

Transition Wavelength	Initial State	Final State	Contribution
563.07 nm	HOMO-3	LUMO+7	72.06 %
	HOMO-3	LUMO+15	2.79 %
	HOMO-3	LUMO+16	2.08 %
	HOMO-2	LUMO+7	2.41 %
	HOMO-1	LUMO+7	15.72 %
564.04 nm	HOMO-3	LUMO+7	4.29 %
	HOMO-2	LUMO+7	68.21 %
	HOMO-2	LUMO+15	2.65 %
	HOMO	LUMO+7	16.74 %
570.27 nm	HOMO-3	LUMO+6	12.44 %
	HOMO-2	LUMO+6	5.79 %
	HOMO-1	LUMO+6	73.07 %
	HOMO-1	LUMO+14	2.03 %
	HOMO-1	LUMO+17	2.24 %
570.66 nm	HOMO-2	LUMO+6	13.99 %
	HOMO-1	LUMO+6	2.31 %
	HOMO	LUMO+6	74.49 %
	HOMO	LUMO+14	2.07 %
	HOMO	LUMO+17	2.27 %

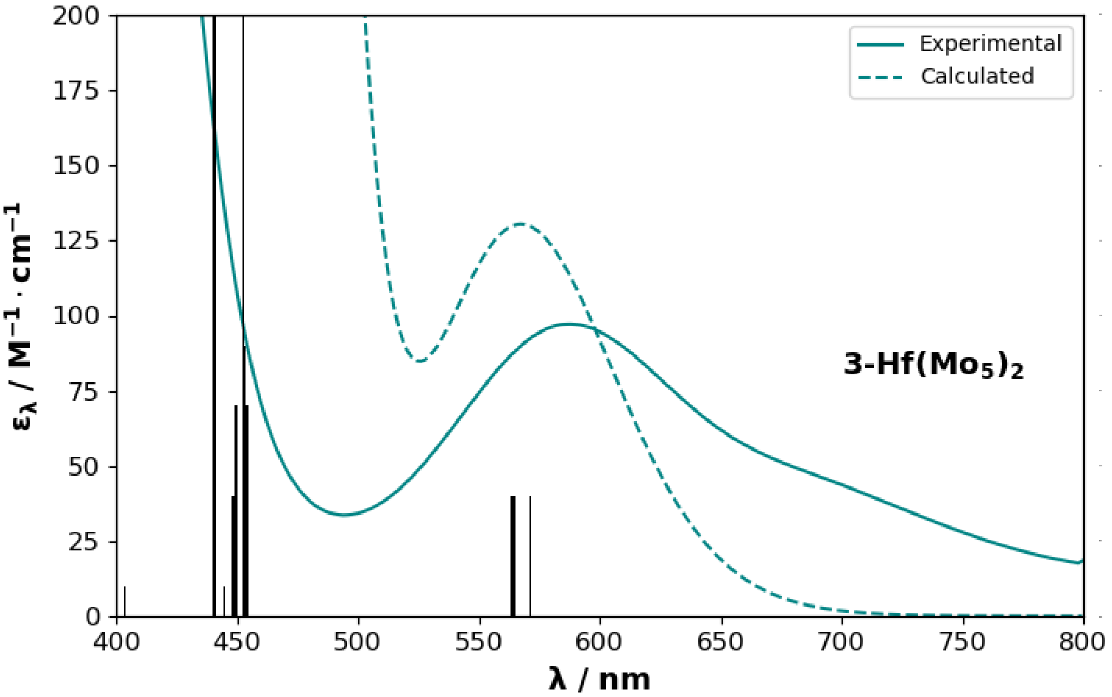

Figure S55. Simulated UV-Vis spectrum of **3-Hf(Mo_5)$_2$**.

Table S8. Orbitals that are involved in the relevant transitions observed in the simulated UV-Vis spectrum of **3-Hf(Mo$_5$)$_2$**.

Transition Wavelength	Initial State	Final State	Contribution
563.83 nm	HOMO-3	LUMO+7	74.02 %
	HOMO-3	LUMO+13	2.25 %
	HOMO-3	LUMO+16	2.04 %
	HOMO-2	LUMO+7	3.31 %
	HOMO-1	LUMO+7	13.83 %
564.77 nm	HOMO-3	LUMO+7	5.10 %
	HOMO-2	LUMO+7	69.26 %
	HOMO-2	LUMO+13	2.10 %
	HOMO	LUMO+7	15.98 %
571.08 nm	HOMO-3	LUMO+6	10.69 %
	HOMO-2	LUMO+6	5.29 %
	HOMO-1	LUMO+6	76.31 %
	HOMO-1	LUMO+12	2.00 %
	HOMO-1	LUMO+17	2.29 %
571.39 nm	HOMO-2	LUMO+6	13.49 %
	HOMO	LUMO+6	76.23 %
	HOMO	LUMO+12	2.00 %
	HOMO	LUMO+17	2.28 %

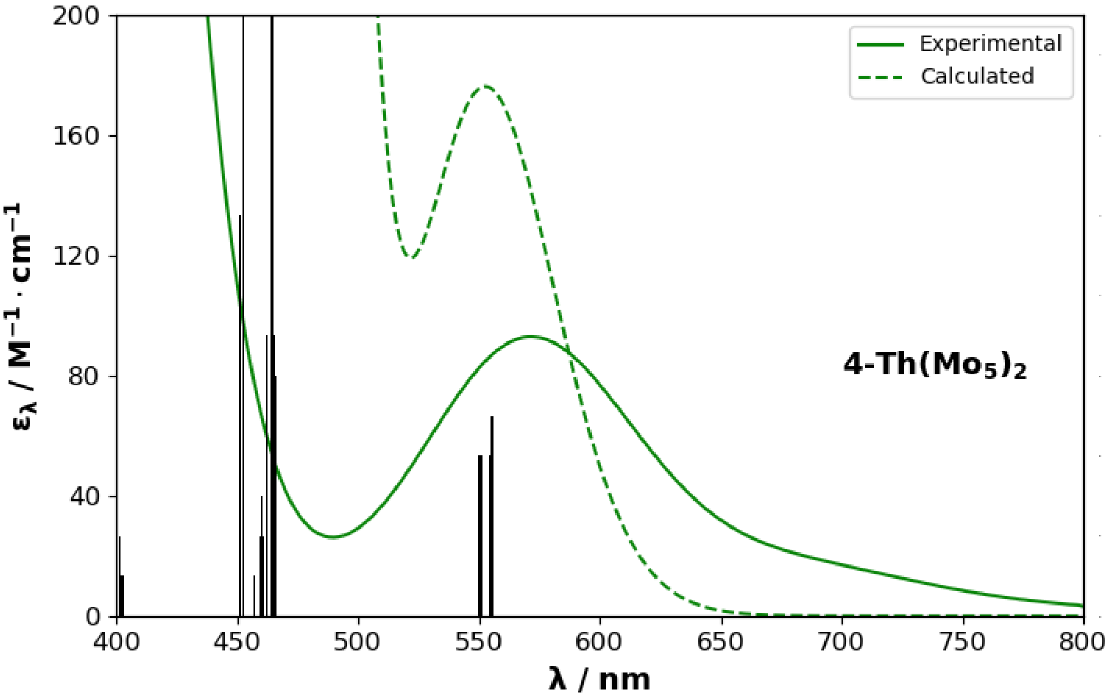

Figure S56. Simulated UV-Vis spectrum of **4-Th(Mo_5)$_2$**.

Table S9. Orbitals that are involved in the relevant transitions observed in the simulated UV-Vis spectrum of **4-Th(Mo$_5$)$_2$**.

Transition Wavelength	Initial State	Final State	Contribution
550.25 nm	HOMO-3	LUMO+7	69.22 %
	HOMO-3	LUMO+12	3.12 %
	HOMO-3	LUMO+19	3.59 %
	HOMO-2	LUMO+7	8.79 %
	HOMO-1	LUMO+7	6.35 %
	HOMO	LUMO+7	4.26 %
551.34 nm	HOMO-2	LUMO+7	25.55 %
	HOMO-1	LUMO+7	43.95%
	HOMO-1	LUMO+19	2.26 %
	HOMO	LUMO+7	18.35 %
554.56 nm	HOMO-3	LUMO+6	12.73 %
	HOMO-2	LUMO+6	50.35 %
	HOMO-2	LUMO+13	2.04 %
	HOMO-2	LUMO+18	2.52 %
	HOMO-1	LUMO+6	23.80 %
	HOMO	LUMO+6	2.73 %
555.44 nm	HOMO-3	LUMO+6	5.74 %
	HOMO-2	LUMO+6	4.12 %
	HOMO-1	LUMO+6	15.32 %
	HOMO	LUMO+6	64.44 %
	HOMO	LUMO+13	2.59 %
	HOMO	LUMO+18	3.20 %

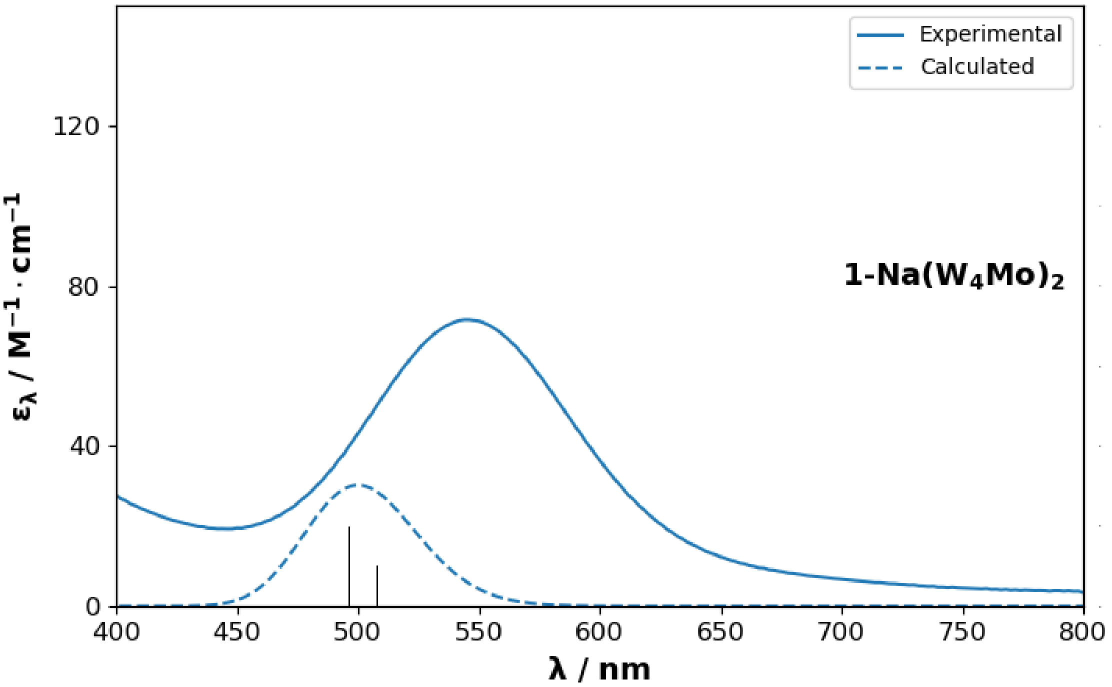

Figure S57. Simulated UV-Vis spectrum of **1-NaW_4Mo**.

Table S10. Orbitals that are involved in the relevant transitions observed in the simulated UV-Vis spectrum of **1-NaW$_4$Mo**.

Transition Wavelength	Initial State	Final State	Contribution
496.43 nm	HOMO-1	LUMO	97.90 %
507.90 nm	HOMO	LUMO	97.35 %

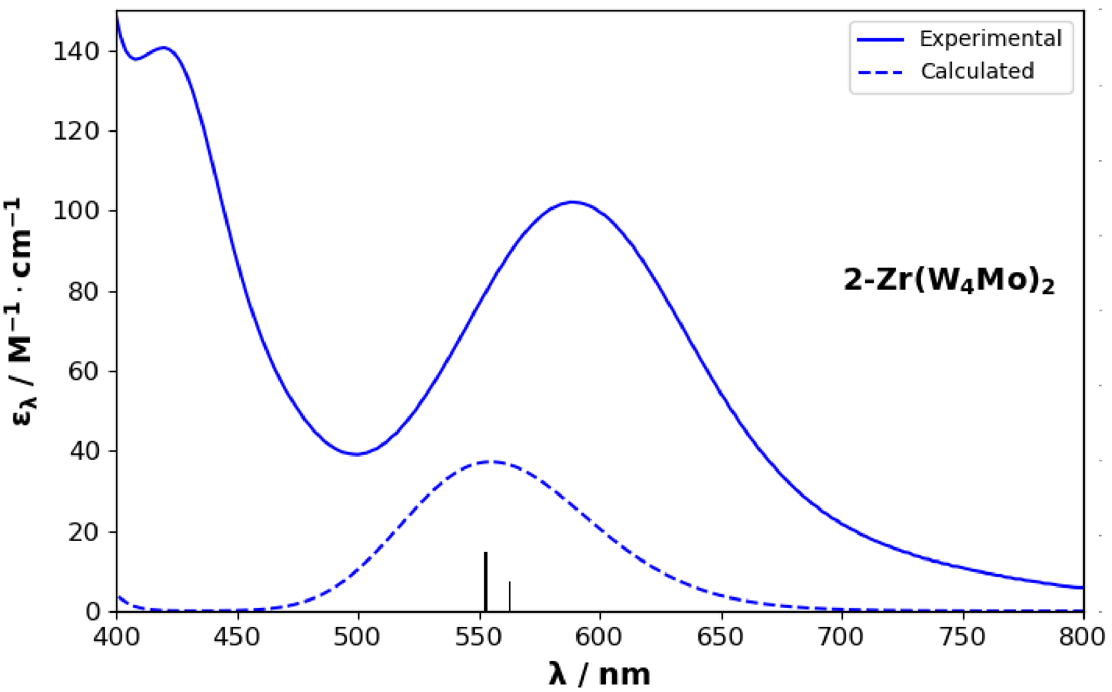

Figure S58. Simulated UV-Vis spectrum of **2-Zr(W_4Mo)$_2$**.

Table S11. Orbitals that are involved in the relevant transitions observed in the simulated UV-Vis spectrum of **2-Zr(W_4Mo)$_2$**.

Transition Wavelength	Initial State	Final State	Contribution
552.90 nm	HOMO-3	LUMO	89.04 %
	HOMO-2	LUMO	9.20 %
553.45 nm	HOMO-3	LUMO+1	8.75 %
	HOMO-2	LUMO+1	88.22 %
562.67 nm	HOMO-1	LUMO+1	75.23 %
	HOMO	LUMO+1	21.54 %

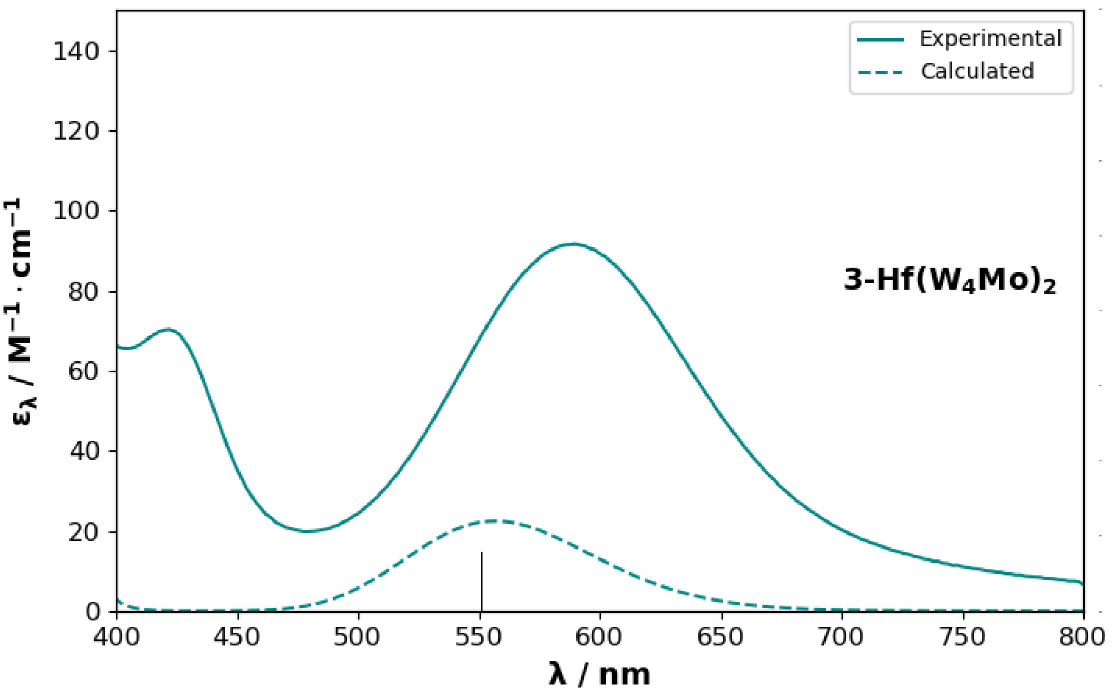

Figure S59. Simulated UV-Vis spectrum of **3-Hf(W_4Mo)$_2$**.

Table S12. Orbitals that are involved in the relevant transitions observed in the simulated UV-Vis spectrum of **3-Hf(W_4Mo)$_2$**.

Transition Wavelength	Initial State	Final State	Contribution
556.70 nm	HOMO-3	LUMO+1	39.48 %
	HOMO-2	LUMO+1	56.25 %
	HOMO-1	LUMO+1	2.02 %
556.87 nm	HOMO-3	LUMO	56.75 %
	HOMO-2	LUMO	39.96 %

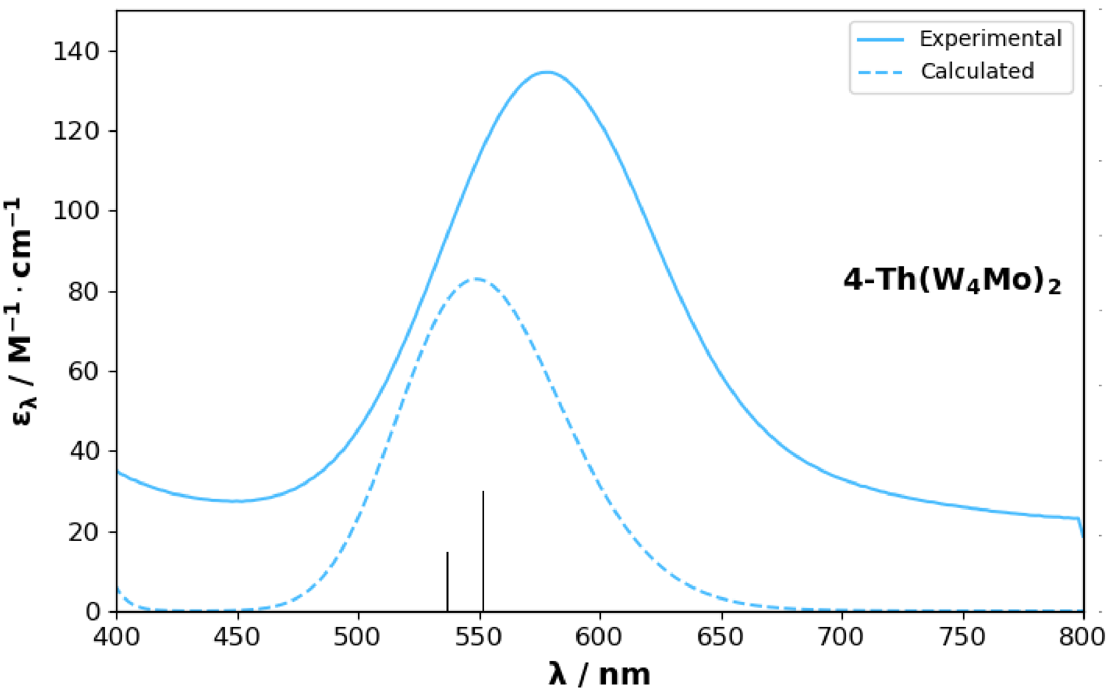

Figure S60. Simulated UV-Vis spectrum of **4-Th(W$_4$Mo)$_2$**.

Table S13. Orbitals that are involved in the relevant transitions observed in the simulated UV-Vis spectrum of **4-Th(W_4Mo)$_2$**.

Transition Wavelength	Initial State	Final State	Contribution
536.91 nm	HOMO-3	LUMO+1	96.83 %
551.76 nm	HOMO-2	LUMO	77.82 %
	HOMO-1	LUMO	11.50 %
	HOMO	LUMO	9.03 %
552.07 nm	HOMO-2	LUMO	8.18 %
	HOMO-1	LUMO	85.47 %
	HOMO	LUMO	4.47 %

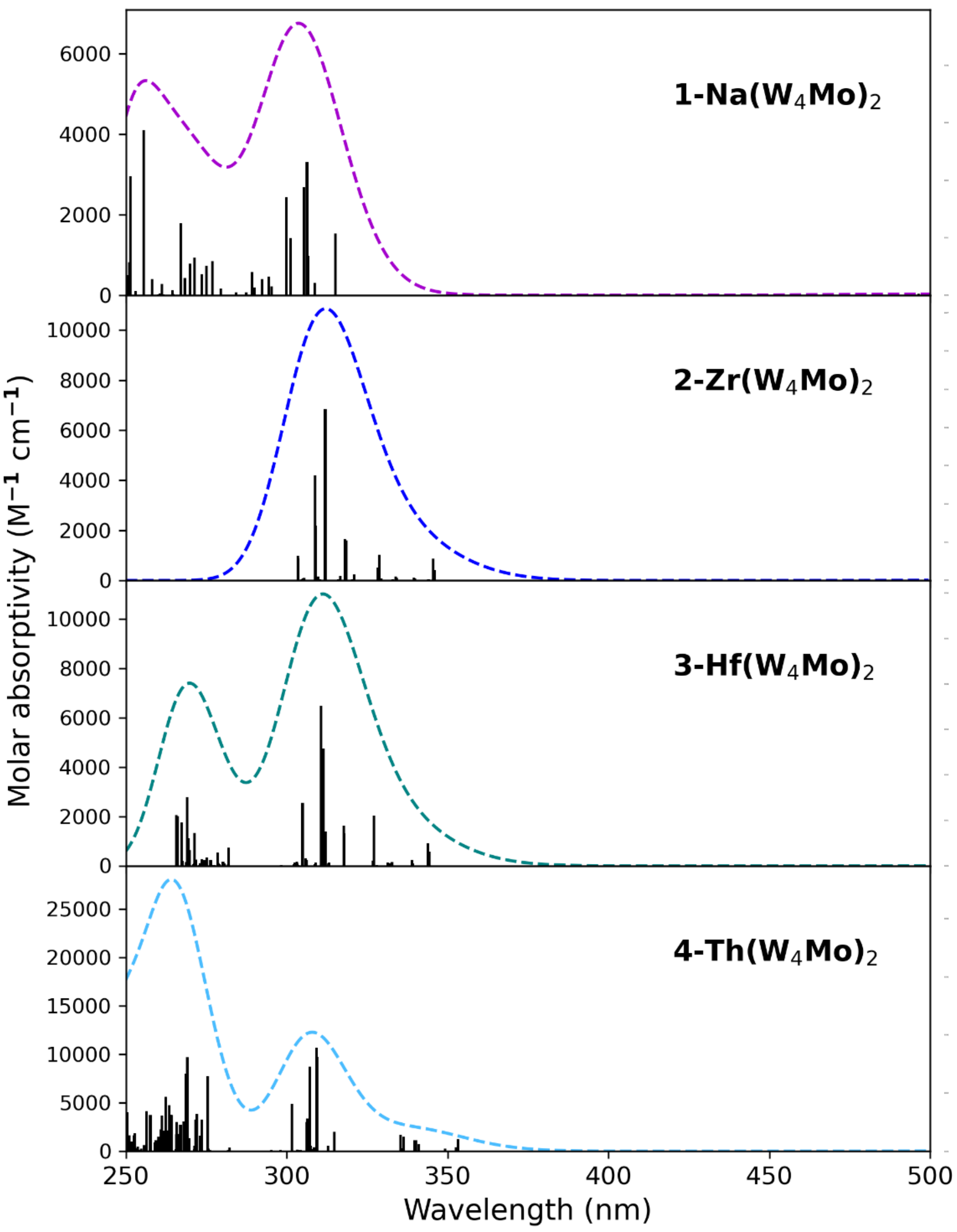

Figure S61. Higer energy region of simulated spectra obtained from TD-DFT calculations. The vertical bars correspond to the dimensionless oscillator strength from which the spectra were convoluted, assuming a gaussian shape (see section S7 for the convolution details).

Table S14. Orbitals that are involved in high-energy transitions observed in the simulated UV-Vis spectrum of **2-Zr(W_4Mo)$_2$**.

Transition Wavelength	Initial State	Final State	Contribution
312.05 nm	HOMO-6	LUMO	44.93 %
	HOMO-7	LUMO	28.49 %
	HOMO-7	LUMO+1	7.14 %
	HOMO-8	LUMO	3.45 %
311.99 nm	HOMO-6	LUMO+1	39.38 %
	HOMO-7	LUMO+1	33.47 %
	HOMO-6	LUMO	8.37 %
	HOMO-7	LUMO	5.94 %
	HOMO-4	LUMO+1	2.02 %

Table S15. Orbitals that are involved in high-energy transitions observed in the simulated UV-Vis spectrum of **3-Hf(W_4Mo)$_2$**.

Transition Wavelength	Initial State	Final State	Contribution
311.50 nm	HOMO-4	LUMO	7.84 %
	HOMO-3	LUMO+22	6.72 %
	HOMO-9	LUMO	6.15 %
	HOMO-2	LUMO+20	5.99 %
	HOMO-3	LUMO+23	5.07 %
	HOMO-8	LUMO+1	4.82 %
	HOMO	LUMO+21	4.56 %
	HOMO-1	LUMO+23	4.33 %
	HOMO-4	LUMO+1	4.24 %
	HOMO-5	LUMO+1	3.94 %
	HOMO-1	LUMO+22	3.75 %
	HOMO-2	LUMO+21	3.13 %
	HOMO-2	LUMO+25	2.88 %
	HOMO	LUMO+20	2.58 %
	HOMO-5	LUMO	2.55 %
310.89 nm	HOMO-6	LUMO	31.32 %
	HOMO-7	LUMO	25.91 %
	HOMO-8	LUMO	6.57 %
	HOMO-5	LUMO	5.51 %
	HOMO-4	LUMO+22	2.82 %
	HOMO-3	LUMO+21	2.13 %
310.72 nm	HOMO-6	LUMO+1	35.57 %
	HOMO-7	LUMO+1	29.73 %
	HOMO-9	LUMO+1	5.64 %
	HOMO-5	LUMO+1	2.90 %
	HOMO-1	LUMO+22	2.29 %
	HOMO-6	LUMO	2.04 %

Table S16. Orbitals that are involved in high-energy transitions observed in the simulated UV-Vis spectrum of **4-Th(W_4Mo)$_2$**.

Transition Wavelength	Initial State	Final State	Contribution
309.48 nm	HOMO-7	LUMO	36.63 %
	HOMO-8	LUMO	24.39 %
	HOMO-6	LUMO	18.91 %
	HOMO-9	LUMO	2.73 %
309.32 nm	HOMO-7	LUMO	41.33 %
	HOMO-8	LUMO	24.62 %
	HOMO-6	LUMO	14.61 %
	HOMO-5	LUMO	6.21 %
	HOMO-9	LUMO	4.27 %

S8. References

(1) Valerio, L. R.; Shiels, D.; Lopez, L. M.; Mitchell, A. W.; Zeller, M.; Bart, S. C.; Matson, E. M. Venturing Past Uranium: Synthesis of a Np(IV) Polyoxomolybdate–Alkoxide Sandwich Complex. *Inorg. Chem.* **2024**, *63*, 22639-22649.

(2) Filowitz, M.; Ho, R. K. C.; Klemperer, W. G.; Shum, W. Oxygen-17 nuclear magnetic resonance spectroscopy of polyoxometalates. 1. Sensitivity and resolution. *Inorg. Chem.* **1979**, *18*, 93-103.

(3) Pascual-Borràs, M.; López, X.; Rodríguez-Fortea, A.; Errington, R. J.; Poblet, J. M. ^{17}O NMR chemical shifts in oxometalates: from the simplest monometallic species to mixed-metal polyoxometalates. *Chem. Sci.* **2014**, *5*, 2031-2042.

(4) Kandasamy, B.; Bruce, P. G.; Clegg, W.; Harrington, R. W.; Rodríguez-Fortea, A.; Pascual-Borrás, M.; Errington, R. J. Bonding Insights from Structural and Spectroscopic Comparisons of {SnW5} and {TiW5} Alkoxido- and Aryloxido-Substituted Lindqvist Polyoxometalates. *Chem. Eur. J.* **2018**, *24*, 2750-2757.

(5) Shiels, D.; Brennessel, W. W.; Crawley, M. R.; Matson, E. M. Leveraging a reduced polyoxomolybdate-alkoxide cluster for the formation of a stable U(V) sandwich complex. *Chem.l Sci.* **2024**, *15*, 11072-11083.

(6) Yan, L.; Lu, Y.; Li, X. A density functional theory protocol for the calculation of redox potentials of copper complexes. *Phys. Chem. Chem. Phys.* **2016**, *18*, 5529-5536.

(7) Rahbani, N.; de Silva, P.; Baudrin, E. Density Functional Theory-Based Protocol to Calculate the Redox Potentials of First-row Transition Metal Complexes for Aqueous Redox Targeting Flow Batteries. *ChemSusChem* **2023**, *16*, e202300482.

(8) Pavlishchuk, V. V.; Addison, A. W. Conversion constants for redox potentials measured versus different reference electrodes in acetonitrile solutions at 25°C. *Inorg. Chim. Acta* **2000**, *298*, 97-102.

(9) Isse, A. A.; Gennaro, A. Absolute Potential of the Standard Hydrogen Electrode and the Problem of Interconversion of Potentials in Different Solvents. *J. Phys. Chem. B* **2010**, *114*, 7894-7899.
